# Retrospective assessment of the quality of diabetes care in a rural diabetes clinic in Western Kenya

**DOI:** 10.1186/s12902-018-0324-5

**Published:** 2018-12-27

**Authors:** Sonak D. Pastakia, Bernardo Nuche-Berenguer, Chelsea Regina Pekny, Benson Njuguna, Elizabeth Guinevere O’Hara, Stephanie Y. Cheng, Jeremiah Laktabai, Victor Buckwalter, Nicholas Kirui, Patrick Chege

**Affiliations:** 1 Moi University School of Medicine, Nandi Hills Road, Eldoret, 30100 Kenya; 2 USAID-Academic Model Providing Access to Healthcare (AMPATH) /Moi Teaching and Referral Hospital, Nandi Hills Road, Eldoret, 30100 Kenya; 3 Purdue Kenya Partnership, Purdue University College of Pharmacy, PO Box 5760, Eldoret, 30100 Kenya; 4 Webuye District Hospital, PO Box 25, Webuye Road, Webuye, 50205 Kenya; 5 National Institute of Diabetes and Digestive and Kidney Diseases, National Institutes of Health, 31 Center Dr, Bethesda, MD 20892 USA; 6 Ohio State University, College of Pharmacy, 500 W 12th Ave, Parks Hall, Columbus, OH 43210 USA; 7 New Mexico VA Health Care System, 1501 San Pedro SE, Albuquerque, NM USA

**Keywords:** Diabetes, Diabetes care, Rural, Sub-saharan Africa, Non-communicable disease

## Abstract

**Background:**

Sub-Saharan Africa continues to face the highest rate of mortality from diabetes in the world due to limited access to quality diabetes care. We assessed the quality of diabetes care in a rural diabetes clinic in western Kenya.

**Methods:**

To provide a comprehensive assessment, a set of clinical outcomes, process, and structure metrics were evaluated to assess the quality of diabetes care provided in the outpatient clinic at Webuye District Hospital. The primary clinical outcome measures were the change in HbA1c and point of care blood glucose. In assessing process metrics, the primary measure was the percentage of patients who were lost to follow up. The structure metrics were assessed by evaluating different facets of the operation of the clinic and their accordance with the International Diabetes Federation (IDF) guidelines.

**Results:**

A total of 524 patients were enrolled into the diabetes clinic during the predefined period of evaluation. The overall clinic population demonstrated a statistically significant reduction in HbA1c and point of care blood glucose at all time points of evaluation after baseline. Patients had a mean baseline HbA1C of 10.2% which decreased to 8.4% amongst the patients who remained in care after 18 months. In terms of process measures, 38 patients (7.3%) were characterized as being lost to follow up as they missed clinic visits for more than 6 months. Through the assessment of structural metrics, the clinic met at least the minimal standards of care for 14 out of the 19 domains recommended by the IDF.

**Conclusion:**

This analysis illustrates the gains made in various elements of diabetes care quality which can be used by other programs to guide diabetes care scale up across the region.

**Electronic supplementary material:**

The online version of this article (10.1186/s12902-018-0324-5) contains supplementary material, which is available to authorized users.

## Background

Diabetes mellitus (DM) is a leading cause for cardiovascular disease (CVD), death and disability in sub-Saharan Africa (SSA). Prevalence of DM in SSA is 7.1% [1], while awareness, treatment and control rates are low [2], with 75% of diabetes related deaths in SSA occurring before the age of 60, a significant blow to a productive population [3]. These dire statistics reflect a sub-optimal infrastructure for DM care in SSA, which needs to be urgently strengthened, particularly given estimates that the prevalence of DM in SSA is expected to more than double by 2045 [3].

Effective diabetes care requires reliable access to recommended diagnosis, treatment, and monitoring for hyperglycemia. There is limited availability of recommended tests for hyperglycemia such as fasting blood glucose [3] measurement, while simpler less, accurate methods for diagnosis such as point-of-care capillary testing are hampered by low availability of glucose strips [2]. Availability of the basic drugs for treatment of diabetes such as metformin, sulfonylureas, and insulin is reportedly low, while treatment monitoring is sub-optimal given the low access to fasting glucose testing and glycated hemoglobin (HbA1C) testing. Additionally, there are potential inaccuracies associated with HbA1C testing in SSA populations. While emphasis on the rapid growth and high mortality burden of diabetes has focused on densely populated urban areas where prevalence is highest [4], these inadequacies are likely compounded in rural SSA populations where even less human and supply chain resources exist [2, 5, 6]. For instance, insulin is available less than 8% of the time in many SSA rural regions. Clinical outcomes are therefore worse, as exemplified by reports of an average life expectancy in rural areas of less than 1 year for children diagnosed with type 1 diabetes [4, 7, 8]. With 61% of the SSA population residing in rural areas, there is an urgent need to rapidly scale up access to high quality diabetes care in these settings. [6, 8–10]

The International Diabetes Federation (IDF) has provided global guidelines for diabetes care which include a set of minimum standards of care that recognise the dearth of resources available in low resource settings [11]. This guideline provides recommendations for the minimal, basic, and comprehensive standards of care which programs should strive for when developing responsive and effective diabetes care programs. These domains include screening, care delivery, education, psychological care, lifestyle management, glucose control targets, clinical monitoring, glucose control with oral therapy, glucose control with insulin therapy, blood pressure control, cardiovascular risk protection, eye screening, kidney damage, foot care, nerve damage, pregnancy, children, and in-patient care. Additional details can be found in the Additional file 1: Figure S1.

In order to respond to these comprehensive diabetes care needs, stakeholders have collaborated to improve diabetes care in rural western Kenya. The Academic Model Providing Access to Healthcare (AMPATH) has established a comprehensive care model for diabetes and other non-communicable diseases (NCDs) that leverages robust existing human immunodeficiency virus (HIV) care infrastructure developed in collaboration with The United States Agency for International Development (USAID). AMPATH is a partnership between Moi University, Moi Teaching and Referral Hospital (Eldoret, Western Kenya) and a consortium of North American universities led by the Indiana University School of Medicine [12]. In collaboration with the Kenyan Ministry of Health (MOH) and Moi Teaching and Referral Hospital, AMPATH has addressed fundamental supply chain and human resource barriers to access for diabetes care in rural Western Kenya [5, 13].

We established a diabetes clinic in Western Kenya, with care standardized in accordance to the IDF global guidelines, and adapted for rural diabetes care through protocols aimed at achieving the minimum recommended standards of care [11]. The aim of this article is to determine the quality of diabetes care by describing relevant clinical, process, and structural outcomes in a rural diabetes clinic in SSA.

## Methods

### Study design, and setting

We conducted a retrospective study in the diabetes clinic of Webuye District Hospital (WDH), a secondary care hospital in rural Western Kenya. The clinic was established in 2009 and serves a low-income population that primarily relies on an agriculture-based economy and job market. The clinic is regularly staffed by clinical officers (diploma level providers equivalent to physician assistants in other settings), nutrition counselors, and a social work team. Clinical officers manage stable patients, defined as patients with well controlled diabetes and low risk for complications, while family medicine residents and consultant physicians review newly enrolled patients. In addition, the physicians, family medicine residents and clinical pharmacists provide care decision support for complicated cases. Patients are scheduled for follow up anytime between one week and three months depending on the patient’s clinical needs. The clinic is able to provide both insulin therapy, oral hypoglycemic agents (sulfonylureas), and metformin, as well as routine tests such as point-of-care blood glucose (Abbott Optimum Xceed**®**), and glycated hemoglobin (HbA1C, Siemens DCA Vantage**®**) at the clinic. The hospital lab provides urea, creatinine, electrolyte measurements, urinalysis, and complete blood count testing, when needed. Insulin dependent patients and/or those at increased risk of diabetes related complications are eligible for inclusion within an intensive self-monitored blood glucose (SMBG) program where patients are provided with glucose monitoring devices and test strips for twice daily SMBG checks and relay them to the clinic once a week via phone calls for dose adjustments by the clinic staff [14]. Patients with additional diagnostic tests or treatment requirements beyond the hospital’s capacity are referred to higher levels of care or private facilities. Clinic and phone based patient encounters are recorded using paper based forms that are transcribed into an electronic database. [11].

This study was approved by the Moi Teaching and Referral Hospital/Moi University School of Medicine Institutional Research and Ethics Committee and the Institutional Review Board of the Indiana University Purdue University Indianapolis (IUPUI). A waiver of informed consent was obtained from both of the institutions as all data being assessed was collected through routine clinical care and was de-identified before being retrospectively assessed.

### Participants and data collection

We included data from all patients with type 1 or type 2 diabetes who received care in the WDH diabetes clinic between July 2009 and September 2011. Data was abstracted from the clinic’s electronic database using a predesigned data collection tool by a trained data manager who also checked the information for completeness and discrepancies by periodically comparing the information to the paper-based records.

Baseline data collected for all patients included demographic and socio-economic data. We collected the following information available for each clinical encounter: random blood sugar (RBS), HbA1C, body mass index (BMI), systolic and diastolic blood pressure, self-reported signs and symptoms of hyperglycemia or hypoglycemia (polyuria, polydipsia, polyphagia, change in vision, loss of consciousness, confusion, diaphoresis, weakness, shaking, dizziness, and headache), self-reported symptoms of peripheral neuropathy (skin numbness, tingling, hot/cold or burning sensation) or peripheral neuropathy detected by use of a 10 g monofilament test on each foot, ocular problems (blurred or cloudy vision, eye itchiness, myopia, and hypermetropia), and prescribed medications. For male patients, data on symptoms of impotence were also collected. Where available, data points were collected for each 3-month clinic visit, up to a period of 18 months after enrollment for each individual patient.

### Outcomes

We considered clinical outcomes, process, and structure metrics when assessing the quality of diabetes care at WDH. The primary clinical outcome of interest was change in HbA1C from baseline to 18 months. Secondary clinical outcomes included the change in HbA1c, blood glucose, BMI, blood pressure, and the frequency of diabetes complications at quarterly intervals. Diabetes complications specifically tracked within routine clinical care included symptomatic hypoglycemia/hyperglycemia, presence of peripheral neuropathy, and ocular complications.

The primary process metric of interest was loss-to-follow up, defined as absence of clinical encounter data for more than six consecutive months, for an individual patient. Secondary process metrics included patterns of medication use and frequency of documentation of recommended diabetes care services. In addition, we compared service delivery at WDH against 19 different domains in the minimal, standard, and comprehensive care recommendations from the IDF Global Guideline for diabetes care [11].

### Statistical analysis

Results were presented descriptively as percentages, means, and medians, with corresponding standard deviation and range, where appropriate. Means and standard deviations were presented for normally distributed data while medians and interquartile ranges were utilized for data with a skewed distribution. Comparison between baseline clinical data and subsequent data was made using the paired t-test, with statistical significance being set to a *p* value < 0.05. STATA 13.1(College Station, TX, USA) was used for all analyses.

## Results

524 patients were enrolled in the WDH diabetes clinic between July 2009 and September 2011. Median age was 58 years (IQR = 19) with only 14 patients (3% of the population) having an age ≤ 20 years and 56% being female. The median diabetes duration prior to enrolment was 4 years. Baseline mean BMI of the male and female participants were 26.0 kg/m^2^ (SD = 9.0) and 27.6 (SD = 6.5) kg/m^2^, respectively. 27% (*n* = 95) of the evaluable population were obese (BMI ≥30 kg/m^2^) with significantly more women (31%, *n* = 63) being obese than men (21%, *n* = 32). Of these 524 patients, 85 were enrolled into the SMBG program for intensive follow-up. Additional baseline characteristics are shown in Table 1 with more detailed descriptions available in a prior publication [15]. Within the 18-month period of database evaluation, 60% of the patients had follow up data for at least 6 months while 31% of patients had follow up data for at least 18 months (Table 2).

**Table 1:** - Baseline demographic and clinical values

Number of Patients	524
Median Age (IQR)	58 (19)
Median diabetes duration (IQR)	4 (18)
Age category on enrollment in the clinic (years)	
11–15, n (%)	7 (1.5%)
16–20	7 (1.5%)
21–25	10 (2.1%)
26–30	6 (1.2%)
31–40	24 (5.0%)
41–50	91 (18.8%)
51–60	130 (26.9%)
61–70	120 (24.8%)
71–80	71 (14.67%)
> 80	18 (3.7%)
Female, n (%)	295 (56.3%)
Male, n (%)	229 (43.7%)
HBA1C mean ± SEM, %	10.2 ± 2.8
< 7%, n (%)	63 (15.0%)
7–10%	150 (35.6%)
> 10%	208 (49.4%)
Systolic blood pressure, mean ± SD mmHg	134 ± 24
Diastolic blood pressure, mean ± SD mmHg	83 ± 12
< 120/80 (Normal) n (%)	98 (19.1%)
120–139/80–89 (Prehypertension)	277 (53.9%)
140–159/90–99 (Hypertension, Stage 1)	112 (21.8%)
> 160/100 (Hypertension, Stage 2)	27 (5.3%)
Mean BMI ± SD (Kg/m2)	27.15 ± 6.55
Male, Mean BMI ± SD (n)	26.0 ± 9.0 (150)
Female	27.6 ± 6.5 (202)
< 18.50, n (%)	23 (6.7%)
18.50–24.99	108 (31.1%)
25–29.99	125 (36.0%)
Obese class I range of 30.00–34.99	52 (15.0%)
Obese class II range of 35.00–39.99	26 (7.5%)
≥ 40.00	13 (3.8%)

*SEM* Standard Error of the Mean, *SD* Standard Deviation, *IQR* Interquartile range

**Table 2:** - Duration of Follow-up for Diabetes Clinic Patients

Duration of Follow-up	Total (n = 524)	SMBG (n = 85)	Non-SMBG (n = 436)
Baseline encounter only n (%)	101 (19.2%)	6 (7.1%)	95 (21.7%)
At least 1 month	418 (79.7%)	78 (91.7%)	340 (77.9%)
3 months	378 (72.1%)	75 (88.2%)	303 (69.4%)
6 months	316 (60.3%)	66 (77.6%)	250 (57.3%)
9 months	282 (53.8%)	62 (72.9%)	220 (50.4%)
12 months	235 (44.8%)	58 (68.2%)	177 (40.6%)
15 months	195 (37.2%)	51 (60.0%)	144 (33.0%)
> 18 months	161 (30.7%)	42 (49.4%)	119 (27.2%)

*SMBG* Self-Monitored Blood Glucose

### Clinical outcomes

Mean HbA1c decreased from a baseline of 10.2% by 10% for each subsequent clinic visit compared to the enrolment HbA1c. This reduction was statistically significant for each interval comparison for the SMBG group, the non-SMBG group (usual clinic care), and for the overall group. Significance was primarily driven by patients with higher baseline HbA1c (> 10%) who showed a statistically significant mean decrease of 31.6% at 12 months, while patients with an HbA1c between 7 and 10% showed a modest and non-significant mean decrease in HbA1c of 2.7%. Participants in the SMBG group had the highest baseline HbA1C at 12.4% and showed the largest drop after 3 months of care to 9.6% (Table 3).

**Table 3:** - Mean HbA1c at quarterly intervals within the overall, self-monitored blood glucose and regular clinic populations

Time	Mean HbA1c (%) ± SD Overall (evaluable patients)	Mean HbA1c (%) ± SD Self monitored blood glucose (evaluable patients)	Mean HbA1c (%) ± SD Regular care (evaluable patients)
0 months	10.2 ± 2.8 (n = 421)	12.4 ± 0.3 (n = 69)	9.9 ± 0.1 (n = 353)
3 months	8.9 ± 2.3 (n = 130)*	9.6 ± 0.4 (n = 34)*	8.6 ± 0.2 (n = 96)*
6 months	8.7 ± 2.5 (n = 123)*	9.7 ± 0.5 (n = 26)*	8.5 ± 0.2 (n = 101)*
9 months	9.2 ± 2.6 (n = 129)*	10.1 ± 0.4 (n = 41)*	8.8 ± 0.3 (n = 85)*
12 months	8.5 ± 2.1 (n = 87)*	9.6 ± 0.4 (n = 25)*	8.1 ± 0.2 (n = 62)*
15 months	8.3 ± 1.9 (n = 83)*	8.9 ± 0.4 (n = 25)*	8.0 ± 0.2 (n = 58)*
18 months	8.5 ± 2.2 (n = 69)*	8.5 ± 0.4 (n = 27)*	8.4 ± 0.4 (n = 42)*

*SD* Standard Deviation

*P < 0.05 via paired t test comparing baseline values in each column to subsequent values

Mean blood sugar (based on random blood sugar obtained during the clinic visit) decreased from a baseline value of 10.7 mmol/l by ≥10% for each subsequent clinic visit compared to enrolment RBS. This trend, however, was only statistically significant when comparing months 3, 6, and 12 to the baseline RBS **(**Table 4**)**.

**Table 4:** - Change in Random Blood Sugar, Systolic Blood Pressure, Diastolic Blood Pressure and Body Mass Index

Time	RBS (mmol/l) (mean ± SD, evaluable population)	Systolic Blood pressure (mmHg) (mean ± SD, n = evaluable population)	Diastolic Blood pressure (mmHg) (mean ± SD, n = evaluable population)	BMI Males (kg/m^2^) (mean ± SD, n = evaluable population)	BMI Females (kg/m^2^) (mean ± SD, n = evaluable population)
0 months	10.7 ± 4.9 (n = 512)	134 ± 24 (n = 514)	83 ± 12 (n = 514)	26.0 ± 9.0 (n = 150)	27.6 ± 6.5 (n = 202)
3 months	8.9 ± 3.6 (n = 364)*	136 ± 21 (n = 364)	83 ± 11 (n = 364)	26.3 ± 6.2 (n = 125)	27.7 ± 6.3 (n = 179)
6 months	9.2 ± 4.0 (n = 284)*	136 ± 21 (n = 289)	82 ± 11 (n = 289)	25.9 ± 5.9 (n = 102)	27.6 ± 5.6 (n = 159)
9 months	9.6 ± 4.2 (n = 260)	135 ± 20 (n = 266)	82 ± 11 (n = 266)	26.3 ± 6.1 (n = 92)	27.5 ± 6.4 (n = 143)
12 months	8.9 ± 3.8 (n = 217)*	137 ± 21 (n = 225)	83 ± 13 (n = 225)	26.4 ± 6.3 (n = 80)	27.7 ± 6.3 (n = 117)
15 months	9.0 ± 3.5 (n = 183)	134 ± 22 (n = 188)	81 ± 12 (n = 188)	26.8 ± 6.1 (n = 75)	28.3 ± 6.8 (n = 92)
18 months	9.1 ± 4.1 (n = 157)	136 ± 22 (n = 157)	83 ± 11 (n = 157)	26.8 ± 6.7 (n = 68)	28.0 ± 5.7 (n = 74)

*RBS* Random Blood Sugar, *BMI* Body Mass Index

*P < 0.05 via paired t test comparing baseline values in each column to subsequent values

Statistically significant reductions were not seen with systolic or diastolic blood pressure from the baseline mean blood pressure of 134/83 mmHg. Minimal and non-significant changes in the mean BMI were seen throughout the periods of evaluation. Among individuals with a baseline BMI < 18.5 kg/m^2^, BMI increased over time to a mean BMI of 20 kg/m^2^ by month 18 (Table 5).

**Table 5:** - Progression of BMI based on initial BMI classification

BMI basal group (kg/m^2^)	0 months (mean ± SD, n = evaluable population)	3 months (mean ± SD, n = evaluable population)	6 months (mean ± SD, n = evaluable population)	9 months (mean ± SD, n = evaluable population)	12 months (mean ± SD, n = evaluable population)	15 months (mean ± SD, n = evaluable population)	18 months (mean ± SD, n = evaluable population)
< 18.5	16.6 ± 0.3 n = 33	18.2 ± 0.5* n = 25	18.4 ± 0.5* n = 22	18.7 ± 0.6* n = 21	19.4 ± 0.6* n = 20	19.5 ± 0.7* n = 17	20.0 ± 0.6* n = 17
18.5–24.99	22.2 ± 0.2 n = 108	23.0 ± 0.3 n = 87	22.6 ± 0.3 n = 71	22.9 ± 0.3 n = 67	22.9 ± 0.3 n = 54	23.0 ± 0.3 n = 38	23.2 ± 0.4 n = 32
25–29.99	27.2 ± 0.1 n = 116	27.4 ± 0.2 n = 97	27.6 ± 0.2 n = 89	27.2 ± 0.3 n = 72	27.5 ± 0.3 n = 61	27.2 ± 0.3 n = 50	27.6 ± 0.3 n = 43
30–34.99	32.1 ± 0.2 n = 60	32.1 ± 0.3 n = 52	32.2 ± 0.4 n = 44	32.8 ± 0.6 n = 43	32.2 ± 0.6 n = 34	32.6 ± 0.5 n = 36	32.9 ± 0.6 n = 32
35–39.99	36.4 ± 0.3 n = 21	35.3 ± 0.7 n = 18	35.3 ± 0.8 n = 16	35.3 ± 0.9 n = 14	35.5 ± 1.1 n = 11	34.9 ± 1.3 n = 11	36.1 ± 1.5 n = 7
> 40	49.4 ± 4.5 n = 14	45.5 ± 1.8 n = 10	44.0 ± 1.9 n = 6	44.4 ± 3.4 n = 6	44.9 ± 3.0 n = 7	45.7 ± 3.5 n = 6	46.7 ± 3.8 n = 3
Total	26.9 ± 0.4 n = 352	27.2 ± 0.4 n = 289	27.1 ± 0.4 n = 248	27.1 ± 0.4 n = 223	27.3 ± 0.5 n = 187	27.8 ± 0.5 n = 158	27.7 ± 0.5 n = 134

*BMI* body mass index

*P < 0.05 via paired t test comparing baseline values in each column to subsequent values

In terms of diabetes complications, participants in both the SMBG and regular care groups demonstrated high initial frequencies at enrolment of peripheral neuropathy manifested primarily through foot symptoms, ocular complications, hypoglycemia, and hyperglycemia, which declined over time as illustrated in Table 6.

**Table 6:** - Diabetes associated complications in the self-monitored blood glucose vs regular care groups

Time	Self-monitored blood glucose					Regular Care				
	N	Peripheral neuropathy events (n, %)	Ocular events (n, %)	Hypo-glycemic episodes (n, %)	Hyper-glycemic episodes (n, %)	N	Peripheral neuropathy events (n, %)	Ocular events (n, %)	Hypo-glycemic episodes (n, %)	Hyper-glycemic episodes (n, %)
0 months	85	46 (54%)	45 (53%)	43 (51%)	68 (80%)	440	232 (53%)	243 (55%)	98 (22%)	162 (37%)
3 months	71	42 (59%)	39 (55%)	50 (70%)*	25 (35%)*	296	170 (57%)*	156 (53%)*	84 (28%)*	44 (15%)*
6 months	59	33 (56%)	31 (53%)	30 (51%)	27 (46%)*	230	125 (54%)*	124 (54%)	69 (30%)*	55 (24%)*
9 months	58	33 (57%)	29 (50%)	36 (62%)*	32 (55%)*	208	106 (51%)*	87 (42%)*	66 (32%)*	41 (20%)*
12 months	55	27 (49%)	27 (49%)	29 (53%)*	10 (18%)*	170	78 (46%)	76 (45%)*	33 (19%)*	25 (15%)*
15 months	50	23 (46%)	27 (54%)	16 (32%)*	20 (40%)*	138	64 (46%)*	52 (38%)*	24 (17%)*	27 (20%)*
18 months	43	17 (40%)*	17 (40%)*	17 (40%)*	5 (12%)*	114	44 (39%)*	39 (34%)*	16 (14%)*	17 (15%)*

*P < 0.05 as compared to its own basal value (0 months) (calculated with a binomial test)

### Process outcomes

38 (7.3%) patients in the overall clinical population were lost to follow-up, with their distribution being 6 (7.1%) patients in the SMBG program and 32 patients (7.3%) in usual clinic care. In terms of documentation of recommended diabetes care procedures, assessment for hypoglycemia and hyperglycemia, blood pressure, ophthalmological symptoms, and symptoms of foot disorders were documented for more than 90% of the clinic visits. 85.1% of the evaluable population had an HbA1c result documented twice per year, 2.7% documented having their serum lipids checked at any time point, 1.5% had a creatinine result documented, and nobody had a documented microalbumin test.

A total of 284 patients (58%) were on oral medication, 95 (19%) were only taking insulin, 88 (18%) were taking oral medications and insulin, 16 (3%) were taking mostly oral medication but also insulin at some point in their care, and 6 (1%) were taking mostly insulin but also received trials of oral medications. Patients with diabetes and hypertension were started on various hypertension medications with angiotensin converting enzyme inhibitors (enalapril) being prescribed for 73% of diabetes patients, followed by thiazide-type diuretics (hydrochlorothiazide) for 58% of patients, calcium channel blockers (nifedipine 30.5% and amlodipine 1%), angiotensin receptor blockers (11%), beta blockers (atenolol 11%, carvedilol 2%, and metoprolol 1.3%), loop diuretics (furosemide 7.4%), and aldosterone antagonists (spironolactone 1.1%). Low dose aspirin (75–100 mg) was prescribed for 30% of the clinic population for primary and secondary prevention of cardiovascular events.

In comparing the services available at WDH to the IDF global guideline recommendations, the WDH diabetes clinic provided care that was below the minimal recommendations for care in 5 domains, met the minimal care recommendations in 7 domains, met the standard care recommendations in 7 domains, and didn’t meet the comprehensive care recommendations in any of the domains **(**Table 7**)**. For the domains where the clinic did not meet minimal recommendations, the primary limitations were related to limited lab infrastructure within the hospital where laboratory tests were infrequently available to patients.

**Table 7:** - Grading of the WDH clinic structure metrics against the International Diabetes Federation Guidelines

IDF Domains	Below minimal care	Minimal Care	Standard Care	Comprehensive Care
Screening		X		
Care delivery			X	
Education			X	
Psychological Care	X – Limited access to mental health care referral and medications			
Lifestyle management		X		
Glucose control Targets		X		
Clinical Monitoring			X	
Self-monitoring			X	
Glucose control: oral therapy		X		
Glucose control: insulin therapy			X	
Blood pressure control			X	
Cardiovascular risk protection	X-Statins infrequently used/available			
Eye Screening		X		
Kidney damage	X-limited assessment for proteinuria or routine creatinine			
Foot care		X		
Nerve damage		X		
Pregnancy	X-Approved methods for diabetes screening not routinely done			
Children			X	
In-patient care	X-Limited access to intensive care and limited biochemistry capacity			

## Discussion

This multifaceted evaluation of the quality of care within the WDH rural diabetes clinic provides insight into the unique aspects of implementing diabetes care in rural SSA. Our investigation revealed a statistically significant 10% decrease in HbA1c among enrolled diabetes patients, reflecting improved glycemic control. In addition, we noted a high adherence to recommended diabetes care procedures in this resource constrained setting.

HbA1c is a good marker for glycemic control over 3 months and is recommended regularly for patients with DM because of its unique ability to predict complications [16–18]. HbA1c fell significantly over time for the overall clinic population and in particular, among patients with higher baseline HbA1cs. This may have been a result of their preferential enrolment into the intensive SMBG program. While access to SMBG programs are largely unavailable in the public sector in SSA, this service demonstrated a dramatic reduction in HbA1c for enrollees and is a feasible strategy to stave off costly and difficult to manage diabetes complications in high risk patients [19].

Statistically significant reductions were not seen with BMI in the overall population, an unsurprising finding considering the initial baseline BMI of the diabetes population revealed a lower frequency of obesity in our population than seen in resource-rich settings [15]. In contrast, patients with BMI < 18.5 kg/m^2^ showed a statistically significant increase in BMI over the course of care, a reflection of the unique pathophysiologic presentation of diabetes in this rural setting [20]. With the atypical low BMI presentation exhibited by these patients, providers were generally more inclined to initiate insulin therapy or insulin secretagogues over metformin therapy as the primary mechanism of action and weight loss associated with metformin would not be desirable amongst these patients [21]. Both insulin and insulin secretagogues are known to trigger weight gain and thus may have contributed to the increase among the patients with a low BMI [22].

Use of standardized encounter forms and point of care glucose and HbA1c testing ensured regular assessments of recommended diabetes tests and services, with more than 85% of patients receiving regular HbA1c testing. In contrast, services which could not be performed within the clinic were frequently not provided due to lack of availability of reagents and non-functioning equipment in the hospital lab. These findings highlight a frequently observed trend seen with diabetes care in low-resource settings where logistical challenges have a dramatic negative impact on the care of diabetes patients [2, 23–25].

Our investigation has several limitations which are important to highlight. First, multiple points of documentation were required during clinic visits in a somewhat fragmented process that involved documentation in a MOH diary and a MOH prescription form, in addition to the standardized encounter form. These cumbersome and time-consuming requirements anecdotally led to documentation fatigue among providers and potentially incomplete documentation in the electronic data collection tool. Despite the challenges associated with documentation, the standardized clinical encounter form was crucial to ensuring that the clinic met many of the recommended minimum standards set forth in the IDF guidelines. Second, use of a retrospective assessment over a fixed time period led to varying periods of follow-up for the evaluated patients. 60% of the patients had follow up data for at least 6 months while only 31% of patients had follow up data for at least 18 months. While this approach had multiple challenges with missing data, this approach gives readers a realistic view of the initial observations seen when establishing a specialty diabetes clinic in a rural setting. While it would have been ideal to have 18 months of data on all patients, we were only able to follow a small subset of patients for the entire duration because of the varying dates of enrollment of participants. It is also possible that this follow-up approach could have introduced selection bias as patients who remained engaged in care for 18 months might be more likely to have better outcomes than patients who contributed data over a shorter duration of time. Fortunately, this limitation is mitigated by the low rates of patients lost to follow-up. The clinic was not structured to track the reasons for patients being lost to follow up. Third, the WDH clinic received philanthropic support from several sources which was used to offset the costs of vital laboratory tests and expensive medications such as HbA1c and insulin. This limits generalizability to diabetes clinics where patients shoulder all costs of care and medicines. Nevertheless, our findings suggest that logistical considerations can be effectively addressed if many of the financial barriers are overcome or partially subsidized. Another limitation is that patients with type 1 and type 2 diabetes were analysed together in the same cohort. This analysis strategy was utilized to ensure that the process measures analysed in this study reflected the entire population receiving care in the clinic and were representative of the patient population typically seen in SSA. This decision to analyse patients in a combined fashion was influenced by several of the limitations we have observed within this population and the limited infrastructure in this setting. This includes the frequent observation that patients with type 1 diabetes in SSA tend to have delayed onset of type 1 diabetes compared to other settings and have a relatively higher presence of atypical variants of diabetes which require unique treatment regimens incorporating intermittent insulin [20, 26–28]. Furthermore, the WDH clinic, like most public sector diabetes clinics in SSA, does not have the capacity to perform C-peptide or antibody testing to help confirm the presence of type 1 diabetes [19]. These factors make it difficult to appropriately categorize patients as having type 1 or type 2 diabetes. While this represents a limitation of this study, it is worth noting that only 3% of the population was < 20 years of age at the time of enrollment.

Despite these limitations, our comprehensive analysis provides an in-depth assessment of the unique aspects of providing care in rural resource-limited settings in SSA. The improvements in the glucose control of the population, highlights how barriers to care can be overcome through collaborative partnerships including local providers, international funders, and academic institutions. This analysis has also provided our overarching diabetes clinic operations team with considerable guidance on how to promote continuous quality improvement within our service delivery. In order to address the domains in which the WDH clinic failed to meet the recommended IDF criteria standards for quality care, we have specifically addressed many of the limitations of the clinic by including programs which have studied the ideal methods for assessing gestational diabetes [29], assessed the reliability of point of care lipid testing [30], incorporated point of care testing for creatinine and potassium, tested various screening approaches [31], incorporated additional medications such as statins within the clinic pharmacy [13, 32, 33], implemented community based diabetes care delivery alongside microfinance services [34], and included a mobile point of care electronic medical record system eliminating the need for duplicative data entry [35–37].

## Conclusion

This comprehensive analysis highlights many unique attributes found in a rural diabetes clinic in western Kenya. With the projected increases in diabetes prevalence across SSA, it is essential that lessons are learned from this analysis and responsive, contextualized infrastructure is created to continue expanding access to high quality diabetes services across the region. The many gaps in care and unique attributes found amongst this patient population create a rich platform for future research efforts geared towards addressing the many pressing challenges patients with diabetes in SSA face. A combined approach which simultaneously focuses on the creation of much needed clinical infrastructure for comprehensive diabetes care with contextualized research will help stave off the many anticipated health and economic consequences associated with diabetes in SSA.

## Additional file

Additional file 1: Global Guideline for Type 2 Diabetes. (PDF 533 kb)

## Abbreviations

AMPATH: Academic Model Providing Access to Healthcare
BMI: Body mass index
CVD: Cardiovascular Disease
DM: Diabetes Mellitus
HbA1c: Glycosylated hemoglobin
HIV: Human immunodeficiency virus
IDF: International Diabetes Federation
IQR: Interquartile range
IUPUI: Indiana University Purdue University Indianapolis
mmHg: Millimeters mercury
MOH: Ministry of Health
NCD: Non-communicable diseases
RBS: Random blood sugar
SD: Standard deviation
SEM: Standard error of the mean
SMBG: Self-monitored blood glucose
SSA: Sub-Saharan Africa
USAID: United States Agency for International Development
VA: Veterans Affairs
WDH: Webuye District Hospital
