# Supplementary material for: Retrospective assessment of the quality of diabetes care in a rural diabetes clinic in Western Kenya
Source: BMC Endocr Disord. 2018 Dec 27;18:97. doi: 10.1186/s12902-018-0324-5 (PMC6307239; doi:10.1186/s12902-018-0324-5)
Supplement: Supplementary file 1 — Global Guideline for Type 2 Diabetes. (PDF 533 kb) [file 12902_2018_324_MOESM1_ESM.pdf]

INTERNATIONAL DIABETES FEDERATION, 2005

Clinical Guidelines Task Force

# Global Guideline

## for Type 2 Diabetes

International Diabetes Federation

# Global Guideline

## for Type 2 Diabetes

International Diabetes Federation

## Website and other versions of this document

This document is also available at www.idf.org

Versions of this document aimed at other audiences are planned, in particular a series of articles in *Diabetes Voice* (2006).

## Correspondence, and related literature from IDF

Correspondence to: Professor Philip Home, SCMS-Diabetes, Medical School, Framlington Place, Newcastle upon Tyne, NE2 4HH, UK. philip.home@newcastle.ac.uk

Other IDF publications, including *Guide for Guidelines*, are available from www.idf.org, or from the IDF Executive Office: International Diabetes Federation, Avenue Emile De Mot 19, B-1000 Brussels, Belgium. communications@idf.org

## Acknowledgements, and sponsors' duality of interest

This activity was supported by unrestricted educational grants from:

Eli Lilly
GlaxoSmithKline
Merck Inc (MSD)
Merck Santé
Novo Nordisk
Pfizer Inc
Roche Diagnostics
Sanofi-Aventis
Takeda.

These companies did not take part in the development of the guideline. However, these and other commercial organizations on IDF's communications list were invited to provide comments on draft versions of the guideline (see *Methodology*).

Sylvia Lion of Eli Lilly is also thanked for providing organizational support for the meeting of the Guidelines Group.

## Citation

IDF Clinical Guidelines Task Force. Global guideline for Type 2 diabetes. Brussels: International Diabetes Federation, 2005.

## Copyright

All rights reserved. No part of this publication may be reproduced or transmitted in any form or by any means without the written prior permission of the International Diabetes Federation (IDF). Requests to reproduce or translate IDF publications should be addressed to IDF Communications, Avenue Emile de Mot 19, B-1000 Brussels, by fax at +32-2-5385114, or by e-mail at communications@idf.org

© International Diabetes Federation, 2005

ISBN 2-930229-43-8

# Preface

There is now extensive evidence on the optimal management of diabetes, offering the opportunity of improving the immediate and long-term quality of life of those with the condition.

Unfortunately such optimal management is not reaching many, perhaps the majority, of the people who could benefit. Reasons include the size and complexity of the evidence-base, and the complexity of diabetes care itself. One result is a lack of proven cost-effective resources for diabetes care. Another result is diversity of standards of clinical practice.

Guidelines are one part of a process that seeks to address those problems. Many guidelines have appeared internationally, nationally, and more locally in recent years, but most of these have not used the rigorous new guideline methodologies for identification and analysis of the evidence.

Increasingly, national organizations have sought to use these new approaches, which are described in the IDF publication *Guide for Guidelines*. It was noted in that document that many countries around the world do not have the resources, either in expertise or financially, that are needed to promote formal guideline development. In any case, such a repetitive approach would be enormously inefficient.

Accordingly the International Diabetes Federation (IDF) has developed a global guideline. For reasons of efficiency the current initiative has chosen to use the evidence analyses of prior national and local efforts. This should also help to ensure a balance of views and interpretation.

A global guideline presents a unique challenge. Many national guidelines address one group of people with diabetes in the context of one health-care system, with one level of national and health-care resources. This is not true in the global context where, although every health-care system seems to be short of resources, the funding and expertise available for health care vary widely between countries and even between localities.

Published national guidelines come from relatively resource-rich countries, and may be of limited practical use in less well resourced countries. Accordingly we have also tried to develop a guideline that is sensitive to resource and cost-effectiveness issues. Despite the challenges, we hope to be found to have been at least partially successful in that endeavour, which has used an approach that we have termed 'Levels of care' (see next page).

Funding is essential to an activity of this kind. IDF is grateful to a diversity of commercial partners for provision of unrestricted educational grants.

# Levels of care

All people with diabetes should have access to cost-effective evidence-based care. It is recognized that in many parts of the world the implementation of particular standards of care is limited by lack of resources. This guideline provides a practical approach to promote the implementation of cost-effective evidence-based care in settings between which resources vary widely.

The approach adopted has been to advise on three levels of care:

## Standard care

Standard care is evidence-based care which is cost-effective in most nations with a well developed service base, and with health-care funding systems consuming a significant part of national wealth.

Standard care should be available to all people with diabetes and the aim of any health-care system should be to achieve this level of care. However, in recognition of the considerable variations in resources throughout the world, other levels of care are described which acknowledge low and high resource situations.

## Minimal care

Minimal care is the lowest level of care that anyone with diabetes should receive. It acknowledges that standard medical resources and fully-trained health professionals are often unavailable in poorly funded health-care systems. Nevertheless this level of care aims to achieve with limited and cost-effective resources a high proportion of what can be achieved by *Standard care*. Only low cost or high cost-effectiveness interventions are included at this level.

## Comprehensive care

Comprehensive care includes the most up-to-date and complete range of health technologies that can be offered to people with diabetes, with the aim of achieving best possible outcomes. However the evidence-base supporting the use of some of these expensive or new technologies is relatively weak.

### Summary of the Levels of Care structure

#### Standard care

Evidence-based care, cost-effective in most nations with a well developed service base and with health-care funding systems consuming a significant part of their national wealth.

#### Minimal care

Care that seeks to achieve the major objectives of diabetes management, but is provided in health-care settings with very limited resources – drugs, personnel, technologies and procedures.

#### Comprehensive care

Care with some evidence-base that is provided in health-care settings with considerable resources.

# Methodology

The methodology used in the development of this guideline is not described in detail here, as it broadly follows the principles described in *Guide for Guidelines*.

In summary:

- The process involved a broadly based group of people, including people with diabetes, health-care professionals from diverse disciplines, and people from non-governmental organizations (see *Members of the Guidelines Group*).

- Within the Group, a number of people had considerable experience of guideline development and health economics, and of health-care administration, as well as of health-care development and delivery, and of living with diabetes.

- Geographical representation was from all the IDF regions, and from countries in very different states of economic development (see *Members of the Guidelines Group*).

- In general the evidence analyses used were published evidence-based reviews and guidelines from the last 5 years; those used are referenced within each section. However, members of the Group were asked to identify any more recent publications relevant to the section of the guideline allotted to them, and encouraged to review details of papers referred to in the published guidelines. Key evidence-based reviews and meta-analyses are also referenced.

- The whole Group met to hear the synthesis of the evidence for each section of diabetes care, to address what recommendations should be made, and to make recommendations over what should be in each *Level of care* for each section.

- The results from the meeting were synthesized into written English by a scientific writer with a knowledge of diabetes, with the assistance of the initiative's chairmen; those drafts were then reviewed by the members of the Group who originally worked on each section, and amendments made according to their suggestions.

- The whole draft guideline was sent out for wider consultation to IDF member associations, IDF elected representatives globally and regionally, interested professionals, industry sponsors (of the guideline and of IDF generally), and others on IDF contact lists, a total of 378 invitations. Each comment received was reviewed by the two chairmen and the scientific writer, and changes were made where the evidence-base confirmed these to be appropriate.

- The revised and final guideline is being made available in paper form, and on the IDF website. The evidence resources used (or links to them) will also be made available. Versions are also being made available in descriptive form (in *Diabetes Voice*), and in language made accessible to people without technical medical training.

- Past experience of international diabetes guidelines is that they have a useful lifespan exceeding 5 years. IDF will consider the need for review of this guideline after 3-5 years.

# Members of the Guidelines Group

Monira Al Arouj - Kuwait City, Kuwait
Pablo Aschner - Bogotá, Colombia
Henning Beck-Nielsen - Odense, Denmark
Peter Bennett - Phoenix, USA
Andrew Boulton - Manchester, UK
Nam Han Cho - Suwon, South Korea
Clive Cockram - Hong Kong, SAR China
Ruth Colagiuri - Sydney, Australia
Stephen Colagiuri (joint chair) - Sydney, Australia
Marion Franz - Minneapolis, USA
Roger Gadsby - Coventry, UK
Juan José Gagliardino - La Plata, Argentina
Philip Home (joint chair) - Newcastle upon Tyne, UK
Nigishi Hotta - Nagoya, Japan
Lois Jovanovic - Santa Barbara, USA
Francine Kaufman - Los Angeles, USA
Thomas Kunt - Berlin, Germany / Dubai, UAE
Dinky Levitt - Cape Town, South Africa
Marg McGill - Sydney, Australia
Susan Manley - Birmingham, UK
Sally Marshall - Newcastle upon Tyne, UK
Jean-Claude Mbanya - Yaoundé, Cameroon
Diane Munday - St Albans, UK
Andrew Neil - Oxford, UK
Hermelinda Pedrosa - Brasilia, Brazil
Ambady Ramachandran - Chennai, India
Kaushik Ramaiya - Dar es Salaam, Tanzania
Gayle Reiber - Seattle, USA
Gojka Roglic - Geneva, Switzerland
Nicolaas Schaper - Maastricht, The Netherlands
Maria Inês Schmidt - Porto Alegre, Brazil
Martin Silink - Sydney, Australia
Linda Siminerio - Pittsburgh, USA
Frank Snoek - Amsterdam, The Netherlands
Paul Van Crombrugge - Aalst, Belgium
Paul Vergeer - Utrecht, The Netherlands
Vijay Viswanathan - Chennai, India

## Medical writer

Elizabeth Home - Newcastle upon Tyne, UK

## IDF Secretariat

Catherine Regniers - Brussels, Belgium

## Consultees:

Comments on the draft were received from all IDF regions, coming from national associations, individuals, industry, non-governmental organizations, and IDF officers. All are thanked for their time and valuable input.

## Duality of interest:

Members of the Guidelines Group and consultees are acknowledged as having dualities of interest in respect of medical conditions, and in relationships with commercial enterprises, governments, and non-governmental organizations. No fees were paid to Group members in connection with the current activity. A fee commensurate with the editorial work was however paid to the spouse of one of the chairmen.

# Contents


| | | Page |
|---|---|---|
| 01 | Screening and diagnosis | 8 |
| 02 | Care delivery | 12 |
| 03 | Education | 16 |
| 04 | Psychological care | 19 |
| 05 | Lifestyle management | 22 |
| 06 | Glucose control levels | 26 |
| 07 | Clinical monitoring | 29 |
| 08 | Self-monitoring | 32 |
| 09 | Glucose control: oral therapy | 35 |
| 10 | Glucose control: insulin therapy | 39 |
| 11 | Blood pressure control | 43 |
| 12 | Cardiovascular risk protection | 46 |
| 13 | Eye screening | 51 |
| 14 | Kidney damage | 55 |
| 15 | Foot care | 59 |
| 16 | Nerve damage | 63 |
| 17 | Pregnancy | 66 |
| 18 | Children | 71 |
| 19 | In-patient care | 74 |
| | Acronyms and abbreviations | 78 |

# Screening and diagnosis

01

## Recommendations

### Standard care

SD1 Each health service should decide whether to have a programme to detect people with undiagnosed diabetes.

- This decision should be based on the prevalence of undiagnosed diabetes and on the resources available to conduct the detection programme and treat those who are detected.
- Universal screening for undiagnosed diabetes is not recommended.
- Detection programmes should target high-risk people identified by assessment of risk factors.

SD2 Detection programmes should use measurement of plasma glucose, preferably fasting.

For diagnosis, an oral glucose tolerance test (OGTT) should be performed in people with a fasting plasma glucose ≥5.6 mmol/l (≥100 mg/dl) and <7.0 mmol/l (<126 mg/dl).

SD3 Where a random plasma glucose level ≥5.6 mmol/l (≥100 mg/dl) and <11.1 mmol/l (<200 mg/dl) is detected on opportunistic screening, it should be repeated fasting, or an OGTT performed.

SD4 The WHO 1999 criteria [1] should be used to diagnose diabetes; these include the importance of not diagnosing diabetes on the basis of a single laboratory measurement in the absence of symptoms.

SD5 People with screen-detected diabetes should be offered treatment and care.

This guideline does not deal with lesser degrees of hyperglycaemia detected on screening.

## ■ Comprehensive care

$SD_C1$ Resources should be available for diabetes detection programmes.

$SD_C2$ Investigations to classify type of diabetes (e.g. islet-cell related antibodies, C-peptide, genotyping) should be available.

## ■ Minimal care

$SD_M1$ Detection programmes should be opportunistic and limited to high-risk individuals.

$SD_M2$ Diagnosis should be based on fasting laboratory plasma glucose (preferred) or capillary plasma glucose.

$SD_M3$ If blood glucose testing is not available, the presence of glycosuria, especially with classical symptoms, may be used to diagnose diabetes.

## Rationale

Screening for Type 2 diabetes has important implications for individual health, day-to-day clinical practice, and public health policy. While the early detection and treatment of diabetes seems logical in terms of minimizing complications, there is currently no direct evidence as to whether or not this is beneficial to individuals. Despite this lack of direct evidence, early detection through screening is taking place and is recommended by a number of organizations throughout the world.

The decision about conducting a detection programme should be based on the following considerations [2]:

- ***epidemiological*** - prevalence of undiagnosed Type 2 diabetes
- ***health systems*** - capacity to carry out the screening, provide care for those who screen positive, and implement prevention programmes in those at high risk of future development of diabetes
- ***population*** - acceptability and likely uptake of the screening programme
- ***economic*** - cost of early detection to the health system and to the individual, and relative cost-effectiveness of early detection compared with improving care for people with known diabetes.

## Evidence-base

Diabetes is associated with a range of serious complications which result in reduced quality of life and premature mortality. Early detection and treatment is one strategy proposed for reducing this burden.

### Screening / early detection

Type 2 diabetes has a long asymptomatic pre-clinical phase which frequently goes undetected. At the time of diagnosis, over half have one or more diabetes complications [3]. Retinopathy rates at the time of diagnosis range from 20 % to 40 % [4,5]. Since the development of retinopathy is related to duration of diabetes, it has been estimated that Type 2 diabetes may have its onset up to 12 years before its clinical diagnosis [4].

Of people with Type 2 diabetes, the proportion who are undiagnosed ranges from 30 % to 90 %. Overall, data from countries as diverse as Mongolia [6] and Australia [7] demonstrate that for every person with diagnosed diabetes there is another who has undiagnosed diabetes. Other countries have even higher rates of undiagnosed diabetes – 80 % in Tonga [8] and 60-90 % in Africa [9-11]. However, in the USA only 30 % are undiagnosed [12].

Although there is considerable evidence supporting the benefits of improved blood glucose, blood pressure and blood lipid control in Type 2 diabetes, no randomized controlled studies have assessed the potential benefits of

early diagnosis on outcomes in screen-detected diabetes. Therefore there is only limited indirect evidence suggesting that early detection may be beneficial.

Schneider et al. [13] performed an analysis of a mass-screening programme based on urinary glucose levels, conducted in the former East Germany in the 1960s and 1970s. It suggested that people found to have diabetes by screening had an improved outcome compared with those presenting spontaneously with diabetes.

Fasting plasma glucose (FPG) at diagnosis might serve as a surrogate for the duration of diabetes. A post-hoc analysis of UKPDS showed that the frequency of subsequent complications was related to FPG at study entry [14]. The group with an initial FPG <7.8 mmol/l (<140 mg/dl) had significantly lower rates of all major end-points compared with the ≥10.0 mmol/l (≥180 mg/dl) group and also had significantly lower diabetes-related death rates and myocardial infarction rates compared with the 7.8 to <10.0 mmol/l (140 to <180 mg/dl) group. These findings suggest a benefit of intervening either at lower FPG levels or earlier in the natural history of diabetes, and may be consistent with a benefit derived from early detection.

Studies in progress which may contribute to the knowledge-base on early detection of diabetes are the 'Inter99' study in Copenhagen county, Denmark [15] and the (Anglo-Danish-Dutch) ADDITION study [16].

Screening for diabetes will also identify individuals with lesser degrees of hyperglycaemia who may benefit from interventions to prevent or delay progression to diabetes, and to prevent cardiovascular disease.

### Screening strategies

There are several options for strategies to screen for undiagnosed diabetes. The ultimate choice is based on available resources and a trade-off between sensitivity (the proportion of people with diabetes who test positive on the screening test), specificity (the proportion of people who do not have diabetes who test negative on the screening test), and the proportion of the population with a positive screening test which needs to proceed to diagnostic testing.

Most screening strategies include risk assessment and measurement of plasma glucose, performed either sequentially or simultaneously. Screening tests are followed by diagnostic tests (fasting plasma glucose (FPG) and/or an oral glucose tolerance test (OGTT)) in order to make the diagnosis. References 2 and 17 provide a detailed review of screening options. Combined screening strategies have a sensitivity and specificity in the order of 75 %, and 25 % of the population require diagnostic testing. People who screen negative will need re-testing after 3-5 years. These people should also be offered lifestyle advice to minimize their risk of developing diabetes.

Although the usefulness of urine glucose as a screening test for undiagnosed diabetes is limited because of low sensitivity (21-64 %) [17], specificity is high (>98 %), so it may have a place in low-resource settings where other procedures are not available.

### Diagnosis

Following a positive screening test, diagnostic testing is required. This may either be a confirmatory FPG (≥7.0 mmol/l, >125 mg/dl) or an OGTT. The diagnostic criteria for diabetes adopted by the WHO [1] and American Diabetes Association (ADA) [18] are accepted internationally.

## Consideration

The place of screening for undiagnosed diabetes as part of an overall strategy to reduce the health burden of diabetes is not established. However, many organizations recommend it. The choice of whether to screen or not, and the screening strategy, must be made locally taking into account local considerations.

## Implementation

A clear and transparent decision should be made about whether or not to endorse a screening strategy. If the decision is in favour of screening, this should be supported by local protocols and guidelines, and public and health-care professional education campaigns.

## Evaluation

Number of health-care professionals and services performing screening, proportion of the population being screened, and detection rate of undiagnosed diabetes should be ascertained. Percentage of diagnosed people entering and continuing in care should be measured.

## References


1. World Health Organization. Definition, Diagnosis and Classification of Diabetes Mellitus and its Complications. Report of a WHO Consultation. Part 1: Diagnosis and Classification of Diabetes Mellitus. Geneva: WHO Department of Noncommunicable Disease Surveillance, 1999: 1-59. http://www.who.int
2. World Health Organization. Screening for Type 2 Diabetes. Report of a World Health Organization and International Diabetes Federation meeting. WHO/NMH/MNC/03.1 Geneva: WHO Department of Noncommunicable Disease Management, 2003. http://www.who.int
3. Manley SM, Meyer LC, Neil HAW, Ross IS, Turner RC, Holman RR. Complications in newly diagnosed type 2 diabetic patients and their association with different clinical and biochemical risk factors. UKPDS 6. Diabetes Res 1990; 13: 1-11.
4. Harris MI, Klein R, Welborn TA, Knuiman MW. Onset of NIDDM occurs at least 4-7 yr before clinical diagnosis. Diabetes Care 1992; 15: 815-19.
5. UKPDS Group. UK Prospective Diabetes Study 30: Diabetic retinopathy at diagnosis of type 2 diabetes and associated risk factors. Arch Ophthalmol 1998; 116: 297-303.
6. Suvd B, Gerel H, Otgooloi D, Purevsuren D, Zolzaya G, Roglic G, et al. Glucose intolerance and associated factors in Mongolia: results of a national survey. Diabet Med 2002; 19: 502-08.
7. Dunstan DW, Zimmet PZ, Welborn TA, De Courten MP, Cameron AJ, Sicree RA, et al. The rising prevalence of diabetes and impaired glucose tolerance: The Australian Diabetes, Obesity and Lifestyle Study. Diabetes Care 2002; 25: 829-34.
8. Colagiuri S, Colagiuri R, Na'ati S, Muimuiheata S, Hussain Z, Palu T. The prevalence of diabetes in the Kingdom of Tonga. Diabetes Care 2002; 25: 1378-83.
9. Aspray TJ, Mugusi F, Rashid S, Whiting D, Edwards R, Alberti KG, et al. Essential Non-Communicable Disease Health Intervention Project. Rural and urban differences in diabetes prevalence in Tanzania: the role of obesity, physical inactivity and urban living. Trans R Soc Trop Med Hyg 2000; 94: 637-44.
10. Amoah AG, Owusu SK, Adjei S. Diabetes in Ghana: a community based prevalence study in Greater Accra. Diabetes Res Clin Pract 2002; 56: 197-205.
11. Mbanya JC, Ngogang J, Salah JN, Minkoulou E, Balkau B. Prevalence of NIDDM and impaired glucose tolerance in a rural and an urban population in Cameroon. Diabetologia 1997; 40: 824-29.
12. Harris MI, Flegal KM, Cowie CC, Eberhardt MS, Goldstein DE, Little RR, et al. Prevalence of diabetes, impaired fasting glucose, and impaired glucose tolerance in U.S. adults. The Third National Health and Nutrition Examination Survey, 1988-1994. Diabetes Care 1998; 21: 518-24.
13. Schneider H, Ehrlich M, Lischinski M, Schneider F. Bewirkte das flächendeckende Glukosurie-Screening der 60er und 70er Jahre im Osten Deutschlands tatsächlich den erhofften Prognosevorteil für die frühzeitig entdeckten Diabetiker? Diabetes und Stoffwechsel 1996; 5: 33-38.
14. Colagiuri S, Cull CA, Holman RR. Are lower fasting plasma glucose levels at diagnosis of type 2 diabetes associated with improved outcomes? UKPDS 61. Diabetes Care 2002; 25: 1410-17.
15. Glümer C, Jørgensen T, Borch-Johnsen K. Prevalences of diabetes and impaired glucose regulation in a Danish population: The Inter99 study. Diabetes Care 2003; 26: 2335-40.
16. Lauritzen T, Griffin S, Borch-Johnsen K, Wareham NJ, Wolffenbuttel BHR, Rutten G, et al. The ADDITION study: proposed trial of the cost-effectiveness of an intensive multifactorial intervention on morbidity and mortality among people with Type 2 diabetes detected by screening. Int J Obes 2000; 24 (Suppl 3): S6-S11.
17. Engelgau MM, Narayan KMV, Herman WH. Screening for Type 2 diabetes. Diabetes Care 2000; 23: 1563-80.
18. The Expert Committee on the diagnosis and classification of diabetes mellitus. Follow-up report on the diagnosis of diabetes mellitus. Diabetes Care 2003; 26: 3160-67.

# Care delivery

02

## Recommendations

### ■ Standard care

CD1 Offer care to all people with diabetes, with sensitivity to cultural wishes and desires.

CD2 Encourage a collaborative relationship, by actively involving the person with diabetes in the consultation, and creating opportunities for them to ask questions and express concerns. Ensure that issues important to the person with diabetes are addressed.

CD3 Offer annual surveillance of all aspects of diabetes control and complications to all people with Type 2 diabetes (see *Table CD1*).

CD4 Agree a care plan with each person with diabetes
- review this annually or more often if appropriate
- modify it according to changes in wishes, circumstances and medical findings.

CD5 Use protocol-driven diabetes care to deliver the care plan between annual reviews, at booked routine reviews.

CD6 Provide urgent access to diabetes health-care advice for unforeseen problems.

CD7 Organize care around the person with diabetes.

CD8 Use a multidisciplinary care team with specific diabetes expertise maintained by continuing professional education.

CD9 Ensure that each person with diabetes is recorded on a list of people with diabetes, to facilitate recall for annual complications surveillance.

CD10 Provide telephone contact between clinic visits.

CD11 Consider how people with diabetes, acting as expert patients, and knowing their limitations, together with local/regional/national associations, might be involved in supporting the care delivery of their local health-care team.

CD12 Use data gathered in routine care to support quality assurance and development activities.

## Comprehensive care

$CD_C1$ In general this would be as *Standard care*.

$CD_C2$ The person with diabetes will have access to their own electronic medical record via secure technology from remote sites. They will be able to give permission for any health-care professional to access that record.

$CD_C3$ Decision support systems might be available to the health-care professional, and perhaps to the person with diabetes.

## Minimal care

$CD_M1$ Offer annual surveillance, agree care plans, deliver protocol-driven care, and ensure that each person with diabetes is recorded on a local list of people with diabetes, as for *Standard care*.

$CD_M2$ Organize care around the person living with diabetes, using an appropriately trained health-care professional to deliver the diverse aspects of that care.

Table CD1

A summary of the assessments to be performed at Annual Review (or annually) for each person with Type 2 diabetes

| Assessment topic | Guideline section |
|---|---|
| Self-care knowledge and beliefs | *Education* |
| Lifestyle adaptation and wishes (including nutrition, physical activity, smoking) | *Lifestyle management* |
| Psychological status | *Psychological care* |
| Self-monitoring skills and equipment | *Self-monitoring* |
| Body weight trends | *Lifestyle management* |
| Blood glucose control | *Glucose control; Clinical monitoring* |
| Blood pressure control | *Blood pressure control* |
| Blood lipid control | *Cardiovascular risk protection* |
| Cardiovascular risk | *Cardiovascular risk protection* |
| Erectile dysfunction, neuropathy | *Nerve damage* |
| Foot condition | *Foot care* |
| Eyes | *Eye screening* |
| Kidneys | *Kidney damage* |
| Pre-pregnancy advice (need for) | *Pregnancy* |
| Medication review | — |

## Rationale

The person diagnosed with Type 2 diabetes requires access to immediate and ongoing care. Who provides this care, and where and when, will depend on local circumstances, but it needs to be organized in a systematic way. General principles include: annual review of control and complications; an agreed and continually updated diabetes care plan; and involvement of the multidisciplinary team in delivering that plan, centred around the person with diabetes.

## Evidence-base

Systems underlying structured organization of care for people with diabetes do not easily lend themselves to comparison by randomized controlled trials (RCTs). Much of the literature in this area is descriptive and interventions are often multifaceted. Some aspects of care organization that do not have a strong evidence-base have been adopted as good practice by a wide range of diabetes services across the world. Systematic review of the evidence was undertaken by the Canadian guideline [1] and the UK National Institute for Clinical Excellence (NICE) guideline on Type 1 diabetes [2].

Both guidelines found support for the multidisciplinary approach, with the Canadian guideline citing a systematic review by Renders et al [3]. Involvement of nurses with training in teaching skills and adult education in a number of aspects of diabetes education, and of formally trained dietitians and podiatrists within the specifically relevant areas of diabetes care, was highlighted [2]. Although there is no RCT evidence for annual review of control and complications, this has become the basis for many quality control structures for diabetes care [2,4]. Some of the rationale for annual surveillance in different areas of care is given in individual sections of the current guideline.

The Canadian guideline advocates organizational interventions that have been shown to improve health-care efficiencies, such as databases to provide patient and physician reminders and transfer of information [1,5], while NICE considers a database-driven recall system to be implicit in recommendations for annual surveillance [2]. Evidence for the usefulness of telemedicine (ranging from the telephone to technology for transmission of images) was reviewed by NICE, who recommended its use to improve process and outcomes [2,6], and drew attention to its potential in rural and remote situations.

Protocol-driven care is not specifically addressed by the guidelines, but Davidson has reviewed studies, including RCTs, in which nurses or pharmacists delivered diabetes care following agreed protocols, and found they achieved improved process and outcomes compared with ‘usual care’ within the US health-care system [7,8].

The literature on care plans and patient-held/accessed records is as yet only descriptive, without useful analysis of patient-related outcomes, but the UK National Service Framework finds that these can help to empower people with diabetes [9].

## Consideration

Given the diversity of health-care systems around the world, recommendations in this part of the guideline are presented in very general terms. Flexibility and adaptability would seem to be important principles. Redeployment of underused resources (such as leprosy clinics) may offer opportunities for improved care in some areas. Where databases are not feasible, lists of people with diabetes can be established in simple book form. Telemedicine can encompass anything from telephones allowing access to health-care professional advice to sophisticated data transfer, but any advance in communications technology, or access to it, may offer opportunities for improved organization of care. Empowering patients to find their way in the system through access to their own data and perhaps through use of decision-support tools would seem to be a logical development.

## Implementation

Organization of care to deliver the above recommendations is largely concerned with:

- putting registration, recall and record systems in place to ensure care delivery occurs for all people with diabetes, and
- having the health-care professionals trained and available to provide the appropriate advice.

Simple communications technologies, and personnel support for those, need to be in place. More sophisticated telemedicine and other IT approaches require not just appropriate software and hardware, but again appropriately trained staff, and continuing maintenance.

## Evaluation

Evaluation will show evidence of structured records being appropriately completed as part of recall and appointment systems driven from a list of people with diabetes. Evaluation of proportions of the managed population receiving defined components of care (such as glucose control, eye screening or blood pressure checks) within a 12-month

period should be made regularly. The staff providing the service should be identified, together with evidence of their continued professional training. The existence of appropriate communications equipment and protocols, and arrangements for their use, can be reviewed.

## References

1. Canadian Diabetes Association Clinical Practice Guidelines Expert Committee. Canadian Diabetes Association 2003 Clinical Practice Guidelines for the Prevention and Management of Diabetes in Canada. Canadian Journal of Diabetes 2003; 27(Suppl 2): S14-S16. http://www.diabetes.ca
2. The National Collaborating Centre for Chronic Conditions. Type 1 Diabetes in Adults. National clinical guideline for diagnosis and management in primary and secondary care. http://www.rcplondon.ac.uk/pubs/books/DIA/index.asp
3. Renders CM, Valk GD, Griffin SJ, Wagner EH, Eijk JThM van, Assendelft WJJ. Interventions to improve the management of diabetes in primary care, outpatient, and community settings: a systematic review. Diabetes Care 2001; 24: 1821-33.
4. European Diabetes Policy Group 1999. A desktop guide to Type 2 diabetes mellitus. Diabet Med 1999; 16: 716-30. http://www.staff.ncl.ac.uk/philip.home/guidelines
5. Griffin S, Kinmonth AL. Diabetes care: the effectiveness of systems for routine surveillance for people with diabetes. Cochrane Database Syst Rev 2000 (2) CD000541
6. Klonoff DC. Diabetes and telemedicine. Is the technology sound, effective, cost-effective and practical? Diabetes Care 2003; 26: 1626-28.
7. Davidson MB. The case for "outsourcing" diabetes care. Diabetes Care 2003; 26: 1608-12.
8. Davidson MB. More evidence to support "outsourcing" of diabetes care. Diabetes Care 2004; 27: 995.
9. Department of Health. National Service Framework for Diabetes: Delivery Strategy. London: Department of Health, 2002. http://www.doh.gov.uk/nsf/diabetes/research

# Education

03

## Recommendations

### ■ Standard care

ED1 Make structured patient education an integral part of the management of all people with Type 2 diabetes:

- from around the time of diagnosis
- on an ongoing basis, based on annual assessment of need
- on request.

ED2 Use an appropriately trained multidisciplinary team to provide education to groups of people with diabetes, or individually if group work is considered unsuitable. Where desired, include a family member or friend.

ED3 Include in education teams a health-care professional with specialist training in diabetes and delivery of education for people with diabetes.

ED4 Ensure that education is accessible to all people with diabetes, taking account of culture, ethnicity, psychosocial, and disability issues, perhaps delivering education in the community or at a local diabetes centre, and in different languages.

ED5 Use techniques of active learning (engagement in the process of learning and with content related to personal experience), adapted to personal choices and learning styles.

ED6 Use modern communications technologies to advance the methods of delivery of diabetes education.

### ■ Comprehensive care

$ED_C1$ This would be as for *Standard care* but would also include the availability on demand of individual advice, through a named key contact.

### ■ Minimal care

$ED_M1$ This would be as for *Standard care* but education would be provided by an appropriately skilled individual rather than a team.

$ED_M2$ Consider how available technologies can best be used to deliver education.

## Rationale

Education in the broadest sense underpins diabetes care, at every contact between the person with diabetes and the health-care team. This has made it difficult to isolate those aspects of education which best contribute to its effectiveness. Recognition that 95 % of diabetes care is provided by people with diabetes themselves, and their families, is reflected in the current terminology of 'diabetes self-management education' (DSME) programmes. With the understanding that knowledge itself is not enough to enable people to change behaviour and improve outcomes [1,2], new approaches emphasizing active learning have been introduced and continue to be developed.

## Evidence-base

Systematic reviews of the evidence are generally critical of the quality of reporting and methodology in many of the studies in this field, and point out the need for further research, and possible strategies for this [3-7]. In the technology report informing its guidance on the use of patient-education models, NICE provided a review, rather than formal meta-analysis, due to differences in design, duration, outcome measures and reporting of studies [4].

NICE excluded foot self-care education but otherwise reviewed the evidence on both general and focused self-management education in Type 2 diabetes. The evidence from eight trials (6 RCTs, 2 CCTs) suggested that general self-management education has a limited impact on clinical outcomes, although few long-term data were available. The evidence from eight trials (7 RCTs, 1 CCT) of focused self-management education (focused on one or two aspects of self-management) suggested that this may have some effect in reducing or maintaining $HbA_{1c}$ levels, although there was little evidence of impact on other clinical outcomes, partly because of short study durations. Also reviewed were four trials (3 RCTs, 1 CCT) that included people with Type 1 or Type 2 diabetes, where there was some evidence that education may improve glycaemic control and quality of life, but little evidence about the longer-term benefits of education. The other reviews painted a similar picture of educational interventions producing modest improvements in glycaemic control [5-7]. The NICE review commented that generally those studies reporting significant results used group interventions [4].

NICE found that costs depended on the type of programme offered, starting with a diabetes centre-based teaching programme spread over three afternoons. Although there is very little evidence regarding the cost-effectiveness of patient education in general, it was concluded that, given the relatively small costs associated with educational programmes, only small improvements in terms of morbidity or health-related quality of life were needed to make educational interventions cost effective [4].

## Consideration

Despite the patchy evidence, certain common principles emerge and are reflected in the recommendations. Assessment of needs is fundamental to tailoring education to the perspective of the person with diabetes, while identified needs of the population served will determine the curriculum. Delivery of advice on nutrition (see *Lifestyle management*) or foot-care (see *Foot care*) or any other aspect of diabetes care would apply the same underlying educational principles outlined in these recommendations. It is noted that diabetes education was an integral part of intensification of care in the DCCT (in Type 1 diabetes), and that nutritional advice made a significant impact in the UKPDS cohort prior to randomization. Accordingly diabetes education is taken as an essential part of diabetes care.

## Implementation

Major components of implementing these recommendations are the recruitment of personnel and their training in the principles of both diabetes education and behaviour change strategies. These staff then need to develop structured education programmes for people with diabetes, supported by suitable education materials matched to the culture of the community served. Attention needs to be given to provision of space in an accessible location, and access to communication tools such as telephones. Levels of literacy and understanding need to be considered.

## Evaluation

NICE suggests measures that could be used, for instance, to audit education for people newly diagnosed with diabetes [4]. These will include the presence of the multidisciplinary team, space and education resources, together with a local curriculum. There will be an entry within individual records of the offering and provision of education around the time of diagnosis, of annual assessment of educational need subsequently, and of provision of such education when the need is identified.

## References


1. Brown SA. Meta-analysis of diabetes patient education research: variations in intervention effects across studies. Res Nurs Health 1992; 15: 409-19.
2. Glasgow RE, Osteen VL. Evaluating diabetes education. Are we measuring the most important outcomes? Diabetes Care 1992; 15: 1423-32.
3. Norris SL, Engelgau MM, Narayan KMV. Effectiveness of self-management training in type 2 diabetes. A systematic review of randomized controlled trials. Diabetes Care 2001; 24: 561-87.
4. NICE. Technology Appraisal 60. Guidance on the use of patient-education models for diabetes. London, National Institute for Clinical Excellence, 2003. http://www.nice.org.uk
5. Piette JD, Glasgow RE. Education and home glucose monitoring. In: Gerstein HC, Haynes RB (eds) Evidence-based Diabetes Care. Hamilton, Ontario: BC Decker, 2001: pp 207-51.
6. Gary TL, Genkinger JM, Gualler E, Peyrot M, Brancati FL. Meta-analysis of randomized educational and behavioral interventions in type 2 diabetes. The Diabetes Educator 2003; 29: 488-501.
7. Warsi A, Wang PS, LaValley MP, Avorn J, Solomon DH. Self-management education programs in chronic disease. A systematic review and methodological critique of the literature. Arch Intern Med 2004; 164: 1641-49.


## Other useful resources

Diabetes patient education is a large topic, and many health-care professionals are unfamiliar with modern educational principles. The following documents are chosen as helpful resources for those wishing to develop materials (curriculum) and skills in this area.

- *IDF Consultative Section on Diabetes Education. International Curriculum for Diabetes Health Professional Education. Brussels: IDF, 2002. www.idf.org*

  This comprehensive document deals with education of the diabetes health-care professionals, and is directed towards (though not solely applicable to) the diabetes educator.

- *European Diabetes Policy Group 1999. A Desktop Guide to Type 2 Diabetes Mellitus. Diabet Med 1999; 16: 716-30. www.staff.ncl.ac.uk/philip.home/guidelines*

  This formal consensus guideline succinctly covers in three pages the appropriate approach to the education of someone with diabetes (initial and ongoing), and some of the content and issues which need to be addressed.

- *Diabetes Education Study Group of the European Association for the Study of Diabetes. Basic Curriculum for Health Professionals on Diabetes Therapeutic Education. 2001. www.desg.org*

  This approachable booklet sets out step by step to address the issues and skills which need to be understood and acquired by anyone seeking to deploy educational techniques in helping people with diabetes.

- *WHO Working Group Report. Therapeutic Patient Education: Continuing education programmes for healthcare providers in the field of prevention of chronic diseases. Copenhagen: WHO Regional Office for Europe, 1998.*

  This document again addresses the competencies needed by those delivering 'therapeutic patient education', and in so doing addresses to some extent the detail of areas to be covered in delivering a comprehensive education programme.

# Psychological care

04

## Recommendations

### ■ Standard care

PS1 In communicating with a person with diabetes, adopt a whole-person approach and respect that person's central role in their care (see also *Education*, *Lifestyle management*).

Communicate non-judgementally and independently of attitudes and beliefs.

PS2 Explore the social situation, attitudes, beliefs and worries related to diabetes and self-care issues.

Assess well-being and psychological status (including cognitive dysfunction), periodically, by questioning or validated measures (e.g. WHO-5 [1]).

Discuss the outcomes and clinical implications with the person with diabetes, and communicate findings to other team members where appropriate.

PS3 Counsel the person with diabetes in the context of ongoing diabetes education and care.

PS4 Refer to a mental health-care professional with a knowledge of diabetes when indicated. Indications may include: adjustment disorder, major depression, anxiety disorder, personality disorder, addiction, cognitive dysfunction.

### ■ Comprehensive care

$PS_C1$ Principles of communication will be as for *Standard care*.

$PS_C2$ A mental health specialist (psychologist) would be included in the multidisciplinary diabetes care team.

$PS_C3$ Periodic assessment and subsequent discussion would be as for *Standard care*, but could use additional measures [2-4] and computer-based automated scoring systems. The mental health specialist in the team would be able to provide a more comprehensive (neuro)psychological assessment, if indicated.

$PS_C4$ Counselling would be as for *Standard care*, but the mental health specialist in the team would be available to offer psychological counselling, to participate in team meetings, and to advise other team members regarding behavioural issues.

## Minimal care

$PS_M1$ Principles of communication will be as for *Standard care*.

$PS_M2$ Be alert to signs of cognitive, emotional, behavioural and social problems which may be complicating self-care, particularly where diabetes outcomes are sub-optimal.

$PS_M3$ Refer for mental health specialist advice according to local availability of such professionals.

## Rationale

Psychological well-being is itself an important goal of medical care, and psychosocial factors are relevant to nearly all aspects of diabetes management. Being diagnosed with diabetes imposes a life-long psychological burden on the person and his/her family. Having diabetes can be seen as an additional risk factor for developing psychological problems, and the prevalence of mental health problems in individuals with diabetes is therefore likely to exceed that found in the general population. Poor psychological functioning causes suffering, can seriously interfere with daily diabetes self-management, and is associated with poor medical outcomes and high costs [5-7]. More serious psychological disorders need to be identified, and referral to a mental health specialist for diagnosis and treatment considered.

Ways in which health-care professionals can directly or indirectly help resolve behavioural and psychological issues, with the aim to protect and promote emotional well-being (quality of life) can be considered in terms of: 1. communication with the patient; 2. assessment or monitoring; and 3. counselling.

## Evidence-base

Psychosocial aspects of diabetes care are included (to varying extents) in the guidelines from the CDA [8], SIGN [9], NICE (Type 1) [10] and ICSI [11] and, for the first time in 2005, in the ADA standards of care [12]. NICE examined evidence from studies including people with Type 2 diabetes, particularly in the area of depression, which is the only topic addressed by ICSI and (for adults) by SIGN. Depression has been found to be twice as prevalent in people with diabetes compared with the general population [13] and is often under-detected [14].

Evidence-based guidelines for psychosocial care in adults with diabetes have been published under the auspices of the German Diabetes Association (DDG), indicating the level of evidence for psychological interventions in different problem areas [15].

There is RCT support for efficacy of antidepressant treatment (in a mixed group of Type 1 and Type 2 diabetes with major depressive disorder), and for cognitive behaviour therapy (in Type 2 diabetes with major depression) [8,14]. There is growing evidence that psychological counselling can contribute to improved adherence and psychological outcomes in people with diabetes [16]. A systematic review and meta-analysis has shown that, overall, psychological interventions are effective in improving glycaemic control in Type 2 diabetes [17].

## Consideration

People coping with diabetes are more likely to be affected by mental health problems, and self-management is likely to be more difficult in the presence of such disorders. Detection of emotional problems in relatively brief consultations with diabetes professionals is likely to be problematic without a formal or structured approach. Lastly there is a clear need for some basic training for diabetes professionals in management issues in this area, and for appropriate referral pathways to mental health specialists with a knowledge of diabetes for people more seriously affected.

If followed by adequate treatment or referral, screening for mental health problems as part of routine diabetes care can help to improve patient satisfaction and psychological outcomes.

## Implementation

Agreement on the importance of psychological factors, and the underpinning philosophy of empowerment of people with diabetes, implies agreement within the care team on the relevance of psychological issues in diabetes. There is then a need for training of diabetes care team members in communication/interview skills, motivational techniques

and counselling. Training of health-care professionals in the recognition of psychological problems will also be needed. Where resources allow, psychological assessment tools should be made available to diabetes teams, and health-care professionals should be trained in applying assessment/monitoring procedures. Collaboration with mental health specialists who already have an interest in diabetes can help to extend the education/training of other mental health specialists in relation to diabetes.

## Evaluation

Evaluate by number of psychological assessments in a given time-period, level of well-being and satisfaction in the managed population over a period of time (overall and by subgroups), and by number of referrals to mental health specialists, indications and outcomes. The training, and continuing education, of diabetes health-care team members can also be evaluated.

## References


1. Henkel V, Mergl R, Kohnen R, Maier W, Möller H-J, Hegerl U. Identifying depression in primary care: a comparison of different methods in a prospective cohort study. BMJ 2003; 326: 200-01. http://www.who-5.org
2. McHorney CA, Ware JE Jr, Raczek AE. The MOS 36-Item Short-Form Health Survey (SF-36): II. Psychometric and clinical tests of validity in measuring physical and mental health constructs. Med Care 1993; 31: 247-63. http://www.SF-36.org
3. Polonsky WH, Anderson BJ, Lohrer PA, Welch GW, Jacobson AM, Aponte JE, et al. Assessment of diabetes-related distress. Diabetes Care 1995; 18: 754-60. http://www.proqolid.org
4. Radloff LS. The CES-D scale: a self-report depression scale for research in the general population. Appl Psychol Meas 1977; 3: 385-401.
5. de Groot M, Anderson R, Freedland KE, Clouse RE, Lustman PJ. Association of depression and diabetes complications: a meta-analysis. Psychosom Med 2001; 63: 619-30.
6. Lin EH, Katon W, Von Korff M, Rutter C, Simon GE, Oliver M, et al. Relationship of depression and diabetes self-care, medication adherence, and preventive care. Diabetes Care 2004; 27: 2154-60.
7. Egede LE, Zheng P, Simpson K. Comorbid depression is associated with increased health care use and expenditures in individuals with diabetes. Diabetes Care 2002; 25: 464-70.
8. Canadian Diabetes Association Clinical Practice Guidelines Expert Committee. Canadian Diabetes Association 2003 Clinical Practice Guidelines for the Prevention and Management of Diabetes in Canada. Canadian Journal of Diabetes 2003; 27(Suppl 2): S50-S52. http://www.diabetes.ca
9. Scottish Intercollegiate Guidelines Network. SIGN 55. Management of Diabetes, 2001. http://www.sign.ac.uk
10. The National Collaborating Centre for Chronic Conditions. Type 1 Diabetes in Adults. National clinical guideline for diagnosis and management in primary and secondary care. http://www.rcplondon.ac.uk/pubs/books/DIA/index.asp
11. Institute for Clinical Systems Improvement (Bloomington, MN, USA). Management of Type 2 Diabetes Mellitus, 2004. http://www.icsi.org/knowledge
12. American Diabetes Association. Standards of Medical Care in Diabetes. Diabetes Care 2005; 28 (Suppl 1): S4-S36.
13. Anderson RJ, Freedland KE, Clouse RE, Lustman PJ. The prevalence of comorbid depression in adults with diabetes. A meta-analysis. Diabetes Care 2001; 24: 1069-78.
14. Rubin RR, Ciechanowski P, Egede LE, Lin EHB, Lustman PJ. Recognizing and treating depression in patients with diabetes. Current Diabetes Reports 2004; 4: 119-25.
15. Herpertz S, Petrak F, Albus C, Hirsch A, Kruse J, Kulzer B. Psychosoziales und Diabetes mellitus. In: Deutsche Diabetes Gesellschaft (DDG) und Deutsches Kollegium Psychosomatische Medizin (DKPM) (eds) Evidenzbasierte Diabetes-Leitlinie DDG. Diabetes und Stoffwechsel 2003; 12 (Suppl 2). http://www.diabetes-psychologie.de/en/guidelines.htm
16. Snoek FJ, Skinner TC. Psychological counselling in problematic diabetes. Does it help? Diabet Med 2004; 19: 265-73.
17. Ismail K, Winkley K, Rabe-Hesketh S. Systematic review and meta-analysis of randomised controlled trials of psychological interventions to improve glycaemic control in patients with type 2 diabetes. Lancet 2004; 363: 1589-97.

# Lifestyle management

05

## Recommendations

### ■ Standard care

LS1 Advise people with Type 2 diabetes that lifestyle modification, by changing patterns of eating and physical activity, can be effective in controlling many of the adverse risk factors found in the condition.

LS2 Provide access to a dietitian (nutritionist) or other health-care professional trained in the principles of nutrition, at or around the time of diagnosis, offering one initial consultation with two or three follow-up sessions, individually or in groups.

LS3 Provide ongoing counselling and assessment yearly as a routine, or more often as required or requested, and when changes in medication are made.

LS4 Individualize advice on food/meals to match needs, preferences, and culture.

LS5 Advise control of foods with high amounts of sugars, fats or alcohol.

LS6 Integrate drug therapy, where needed, into the individual's chosen lifestyle.

LS7 For people choosing to use fixed insulin regimens, advise consistent carbohydrate intake at meals. For these people, as well as those on flexible meal-time + basal insulin regimens, offer education on assessment of carbohydrate content of different types of foods.

LS8 Provide advice on the use of foods in the prevention and management of hypoglycaemia where appropriate.

LS9 Introduce physical activity gradually, based on the individual's willingness and ability, and setting individualized and specific goals.

LS10 Encourage increased duration and frequency of physical activity (where needed), up to 30-45 minutes on 3-5 days per week, or an accumulation of 150 minutes of physical activity per week.

LS11 Provide guidelines for adjusting medications (insulin) and/or adding carbohydrate for physical activity.

LS12 Both nutrition therapy and physical activity training should be incorporated into more broadly based diabetes self-management training programmes (see *Education*).

LS13 For weight reduction in people with Type 2 diabetes who are obese, it may sometimes be appropriate to consider weight loss medications as adjunct therapy.

## Comprehensive care

$LS_C1$ Advice on lifestyle management will in general be as for *Standard care*.

$LS_C2$ Education might also be provided as a routine for special topics such as label reading, restaurant eating, special occasions.

$LS_C3$ Intensive personal counselling might be offered on a regular basis with a health-care professional specifically trained in the principles of nutrition, to facilitate maintenance of lifestyle modifications and support weight loss or weight maintenance.

$LS_C4$ Exercise testing could be available for those considering programmes of physical activity.

$LS_C5$ Aerobic and resistance training sessions might be available, with individualized testing and education by exercise specialists, and continued support from them.

## Minimal care

$LS_M1$ The principles of lifestyle management are as for *Standard care*.

$LS_M2$ Offer basic nutrition guidelines (healthy food choices) for improved glycaemic control.

$LS_M3$ Advise on ways to reduce energy intake (carbohydrate, fat, alcohol as appropriate).

$LS_M4$ Provide nutritional counselling from someone with training in nutrition therapy, around the time of diagnosis, then as assessed as being necessary, or more often as required or requested.

$LS_M5$ Advise and encourage participation in regular physical activity.

## Rationale

People with Type 2 diabetes often have lifestyles (eating and physical activity) which contribute to their problem. It is essential they receive help soon after diagnosis to consider how they may modify lifestyle in ways which enable them to take control of their blood glucose, blood lipid and blood pressure abnormalities, even if they also require drug therapy in the short or longer term (see *Glucose control: therapy*).

## Evidence-base

Evidence supports the effectiveness of nutrition therapy and physical activity in the prevention and management of Type 2 diabetes [1-4]. This is reflected in the current ADA standards of medical care [5] (which draw on a detailed evidence-based technical review on nutrition [6] and a more recent review on physical activity [2]) and in the Canadian guideline [7]. An earlier UK guideline [8] pointed out that

involvement in a lifestyle study, even in the control group, can be beneficial, but that lifestyle modification can be difficult to achieve and maintain. That guideline expressed some concern over methodological problems in trials of complex and multifactorial interventions. Most studies have been short-term (a problem currently being addressed in a US trial), and we do not yet know the ongoing contribution of lifestyle measures once medication has been introduced, or what kind of support is required on a continuing basis. It may be noted that in the UKPDS initial dietary education was very effective in lowering blood glucose after diagnosis, and that some people were then able to maintain target glucose control for many years by diet modification alone [9,10].

Randomized controlled trials and outcome studies of medical nutrition therapy (MNT) in the management of Type 2 diabetes have reported improved glycaemic outcomes ($HbA_{1c}$ decreases of 1.0-2.0 %, depending on the duration of diabetes). MNT in these studies was provided by dietitians (nutritionists) as MNT only or as MNT in combination with diabetes self-management training. Interventions included reduced energy intake and/or reduced carbohydrate/fat intake, and basic nutrition and healthy food choices for improved glycaemic control. Outcomes of the interventions were measurable by 3 months [6,7,11-15].

In a meta-analysis of non-diabetic people, MNT restricting saturated fats to 7-10 % of daily energy and dietary cholesterol to 200-300 mg daily resulted in a 10-13 % decrease in total cholesterol, 12-16 % decrease in LDL cholesterol and 8 % decrease in triglycerides [16]. An expert committee of the American Heart Association documented that MNT typically reduced LDL cholesterol 0.40-0.65 mmol/l (15-25 mg/dl) [17]. Pharmacological therapy should be considered if goals are not achieved between 3 and 6 months after initiating MNT.

A meta-analysis of studies of non-diabetic people reported that reductions in sodium intake to ≤2.4 g/day decreased blood pressure by 5/2 mmHg in hypertensive subjects. Meta-analyses, clinical trials and expert committees support the role of reduced sodium intake, modest weight loss (4.5 kg), increased physical activity, a low-fat diet that includes fruits, vegetables and low-fat dairy products, and moderate alcohol intake, in reducing blood pressure [18].

A meta-analysis of exercise (aerobic and resistance training) reported an $HbA_{1c}$ reduction of 0.66 %, independent of changes in body weight, in people with Type 2 diabetes [19]. In long-term prospective cohort studies of people with Type 2 diabetes, higher physical activity levels predicted lower long-term morbidity and mortality and increases in insulin sensitivity. Interventions included both aerobic exercise (such as walking) and resistance exercise (such as weight-lifting) [2,20,21].

The Canadian guideline has a section on the management of obesity in Type 2 diabetes, which addresses lifestyle measures and also drug and surgical options [7].

## Consideration

It is noted that in general costs of educational initiatives to change lifestyle are low, because unlike drug therapy they are provided on an intermittent rather than continuing basis. From a health-provider perspective many of the costs fall outside their budget, healthier foods and exercise programmes and equipment generally being a cost met directly by the person with diabetes. For these reasons, and because, for glucose control, the gain from lifestyle modification is greater than that from any individual therapy, lifestyle measures are heavily promoted. Lifestyle modification is, however, sometimes difficult for the individual to maintain in the long term, or to develop further after early changes have been made. Where professional nutritionists are unavailable, it was noted that other health-care professionals should be trained in basic nutritional and other lifestyle education.

## Implementation

Recognition of the importance and cost-effectiveness of lifestyle interventions should drive allocation of resources required for care and self-management training. Implementation demands knowledgeable and competent personnel, and dietitians/nutritionists and other health-care professionals may require training to be effective providers of lifestyle interventions. Consistency of approach to lifestyle issues across the diabetes care team is an important principle here. A process is needed to enable people to gain access to services as required.

Self-management counselling in nutrition (for individuals or groups) has four components: 1. assessment; 2. identification of the nutrition problem; 3. intervention that integrates nutrition therapy into overall diabetes management and implementation of self-management training; and 4. nutrition monitoring and evaluation of outcomes. A similar approach needs to be taken for physical activity. Development of educational materials, or adaptation of them from elsewhere, is needed.

## Evaluation

Services should be able to show the availability of appropriately trained personnel, and records that individuals with diabetes have contact with them around the time of diagnosis and at regular intervals thereafter. Educational

support materials should also be demonstrable. Outcomes can be assessed in terms of improvement in appropriate food choices and amounts, and responses to questioning about physical activity levels and, where appropriate, alcohol consumption. Metabolic measures are, however, likely to be confounded by changes in drug therapies.

## References


1. Pastors JG, Warshaw H, Daly A, Franz M, Kulkarni K. The evidence for the effectiveness of medical nutrition therapy in diabetes management. Diabetes Care 2002; 25: 608-13.
2. Sigal RJ, Kenny GP, Wasserman DH, Castaneda-Sceppa C. Physical activity/exercise and type 2 diabetes. Diabetes Care 2004; 27: 2518-39.
3. Herman WH, Hoerger TJ, Brandles M, Hicks K, Sorensen S, Zhang P, et al. Diabetes Prevention Program Research Group. The cost-effectiveness of lifestyle modification or metformin in preventing type 2 diabetes in adults with impaired glucose tolerance. Ann Intern Med 2005; 142: 323-32.
4. The Diabetes Prevention Program Research Group. Impact of intensive lifestyle and metformin therapy on cardiovascular disease risk factors in the Diabetes Prevention Program. Diabetes Care 2005; 28: 888-94.
5. American Diabetes Association. Standards of Medical Care in Diabetes. Diabetes Care 2005; 28 (Suppl 1): S4-S36.
6. Franz MJ, Bantle JP, Beebe CA, Brunzell JD, Chiasson JL, Garg A, et al. Evidence-based nutrition principles and recommendations for the treatment and prevention of diabetes and related complications. Diabetes Care 2002; 25: 148-98.
7. Canadian Diabetes Association Clinical Practice Guidelines Expert Committee. Canadian Diabetes Association 2003 Clinical Practice Guidelines for the Prevention and Management of Diabetes in Canada. Canadian Journal of Diabetes 2003; 27(Suppl 2). http://www.diabetes.ca
8. McIntosh A, Hutchinson A, Home PD, Brown F, Bruce A, Damerell A, et al. Clinical guidelines and evidence review for Type 2 diabetes: management of blood glucose. Sheffield: ScHARR, University of Sheffield, 2001. http://www.nice.org.uk/pdf/NICE_full_blood_glucose.pdf
9. UK Prospective Diabetes Study Group: Response of fasting plasma glucose to diet therapy in newly presenting type II diabetic patients (UKPDS 7). Metabolism 1990; 39: 905-12.
10. Turner RC, Cull CA, Frighi V, Holman RR. Glycemic control with diet, sulfonylurea, metformin, or insulin in patients with type 2 diabetes mellitus: progressive requirement for multiple therapies (UKPDS 49). JAMA 1999; 281: 2005-12.
11. Goldhaber-Fiebert JD, Goldhaber-Fiebert SN, Tristan ML, Nathan DM. Randomized controlled community-based nutrition and exercise intervention improves glycemia and cardiovascular risk factors in type 2 diabetic patients in rural Costa Rica. Diabetes Care 2003; 26: 24-29.
12. Ziemer DC, Berkowitz KJ, Panayioto RM, El-Kebbi IM, Musey VC, Anderson LA, et al. A simple meal plan emphasizing healthy food choices is as effective as an exchange-based meal plan for urban African Americans with type 2 diabetes. Diabetes Care 2003; 26: 1719-24.
13. Lemon CC, Lacey K, Lohse B, Hubacher DO, Klawitter B, Palta M. Outcomes monitoring of health, behavior, and quality of life after nutrition intervention in adults with type 2 diabetes. J Am Diet Assoc 2004; 104: 1805-15.
14. Polonsky WH, Earles J, Smith S, Pease DJ, Macmillan M, Christensen R, et al. Integrating medical management with diabetes self-management training. A randomized control trial of the Diabetes Outpatient Intensive Treatment program. Diabetes Care 2003; 26: 3048-53.
15. Banister NA, Jastrow ST, Hodges V, Loop R, Gillham MB. Diabetes self-management training program in a community clinic improves patient outcomes at modest cost. J Am Diet Assoc 2004; 104: 807-10.
16. Yu-Poth S, Zhao G, Etherton T, Naglak M, Jonnalagadda S, Kris-Etherton PM. Effects of the National Cholesterol Education Program's Step I and Step II dietary intervention programs on cardiovascular risk factors: a meta-analysis. Am J Clin Nutr 1999; 69: 632-46.
17. Grundy SM, Balady GJ, Criqui MH, Fletcher G, Greenland P, Hiratzka LF, et al. When to start cholesterol-lowering therapy in patients with coronary heart disease: a statement for healthcare professionals from the American Heart Association task force on risk reduction. Circulation 1997; 95: 1683-85.
18. Whitworth JA, Chalmers J. World Health Organization-International Society of Hypertension (WHO/ISH) hypertension guidelines. Clin Exp Hypertension 2004; 26: 747-52.
19. Boulé NG, Haddad E, Kenny GP, Wells GA, Sigal RJ. Effects of exercise on glycemic control and body mass in type 2 diabetes mellitus. A meta-analysis of controlled clinical trials. JAMA 2001; 286: 1218-27.
20. Wei M, Gibbons LW, Kampert JB, Nichaman MZ, Blair SN. Low cardiorespiratory fitness and physical activity as predictors of mortality in men with type 2 diabetes. Ann Intern Med 2000; 132: 605-11.
21. Church TS, Cheng YJ, Earnest CP, Barlow CE, Gibbons LW, Priest EL, et al. Exercise capacity and body composition as predictors of mortality among men with diabetes. Diabetes Care 2004; 27: 83-88.

# Glucose control levels

06

## Recommendations

### ■ Standard care

TT1 Advise people with diabetes that maintaining a DCCT-aligned $HbA_{1c}$ below 6.5 % should minimize their risk of developing complications.

TT2 Provide lifestyle and education support, and titrate therapies, to enable people with diabetes to achieve a DCCT-aligned $HbA_{1c}$ below 6.5 % (where feasible and desired), or lower if easily attained.

TT3 Advise those in whom target $HbA_{1c}$ levels cannot be reached that any improvement is beneficial.

TT4 Sometimes raise targets for people on insulin or sulfonylurea therapy in whom attainment of tighter targets may increase the risk of hypoglycaemic episodes, which may present particular problems for people with other physical or mental impairment.

TT5 Equivalent target levels for capillary plasma glucose levels are <6.0 mmol/l (<110 mg/dl) before meals, and <8.0 mmol/l (<145 mg/dl) 1-2 h after meals.

### ■ Comprehensive care

$TT_C1$ The intervention levels are as for *Standard care*, but it may be possible to devote more resources to achieving lower target levels without adverse impact on health.

### ■ Minimal care

$TT_M1$ The intervention levels are as for *Standard care*, but may need to be based on measurement of plasma glucose levels alone.

Plasma glucose is the preferred measure of most modern laboratories. Whole blood gives lower readings due to the volume occupied by haemoglobin. Capillary blood glucose strips measure the glucose in the plasma of the capillary blood sample, but may be calibrated to give results either as plasma or whole blood glucose (check meter instructions).

## Rationale

The UKPDS established the importance of glucose control in prevention of vascular complications in people with Type 2 diabetes. The issue then arises as to the desirable level of glucose control to be achieved. In an ideal world this would be 'normal', but if the available lifestyle and pharmaceutical therapies are less than optimal in terms of efficacy and adverse effects on quality of life, or if these therapies are expensive, then some compromise (varying between individuals and health-care systems) will be needed. The chosen measures of glucose control ($HbA_{1c}$ and self-monitoring) are discussed elsewhere (see *Clinical monitoring*, *Self-monitoring*) – this section deals with target levels.

The concept of targets is open to criticism – they may be unattainable, they may limit what could be attained, and they may be uneconomic to attain. However, without some form of targeted control of an asymptomatic condition it becomes difficult to promote care at all. Targets are often better thought of as 'assessment levels' and 'intervention levels'.

## Evidence-base

The evidence for a target level of control is rarely the subject of an RCT. However, the epidemiological analyses of the UKPDS [1] can be informative in setting targets. Other evidence will usually come from cohort and cross-sectional epidemiological studies [2,3]. While target levels have been set by a number of organizations (including the ADA [4,5] and IDF (Europe) [6]) and in the NICE Type 2 diabetes [7] and Canadian guidelines [8], they are rarely supported by any kind of formal discussion of literature. There is however a high degree of conformity of the recommendations. The NICE Type 1 diabetes guideline does attempt to derive its recommendations with more rigour, and while this is largely directed to microvascular prevention, the argument relating to prevention of arterial disease in people with Type 1 diabetes can be usefully extrapolated to people with Type 2 diabetes in general [9].

The UKPDS shows that good glucose control is attainable at least in the early years; this is consistent with many other intervention studies of different therapies. The issue of whether a microvascular control threshold might or might not exist for glucose control seems not to be relevant to most people with Type 2 diabetes, as the targets for glucose control for prevention of arterial disease are lower when set separately (by NICE [9] and the European Policy Group [6]); thus the issue is primarily that of arterial risk prevention.

Epidemiological evidence shows a relationship between $HbA_{1c}$ and development of cardiovascular disease even within the normal range of $HbA_{1c}$ [10]. This suggests that normal or even low normal is to be preferred, if attainable at reasonable cost and effort. However, this is virtually never attained in clinical studies of therapies. What is clear is that arterial risk in a population with diabetes (UKPDS) decreases down to a DCCT-aligned $HbA_{1c}$ of 5.5 % (compared with normal range of <6.1 %), the lowest level achieved over time for a significant group of people in that study. Use of glucose-lowering therapies was highly cost-effective in UKPDS [11], and accordingly 6.5 % is the target/intervention level recommended in the NICE Type 1 [9] and Type 2 guidelines [7].

Translation of this into self-monitored capillary (whole blood or plasma calibrated) levels is not simple. The upper level of fasting plasma glucose is usually taken as 5.5 mmol/l (100 mg/dl), which might then equate with a DCCT-aligned $HbA_{1c}$ of 6.1 %. Studies with newer insulins achieving pre-breakfast glucose levels of ~6.0 mmol/l (~110 mg/dl) typically return DCCT-aligned $HbA_{1c}$ results of ~7.0 % [12], but glucose profiles in these studies show rising glucose levels through the day, explaining the inconsistency. Regression equations between capillary measured whole blood glucose or plasma glucose and $HbA_{1c}$ referable to the DCCT assay have been published for Type 1 diabetes [13,14], but these combine pre-prandial and post-prandial tests through the day, and reflect the different profiles of glucose control seen in that type of diabetes.

The case for targeting post-prandial blood glucose control can be made on many grounds, none of them RCT-based. Overall the case is compelling, not least by the simple logical observation that the outcome trials have established the utility of lowering blood glucose levels overall, while the highest levels of the day are generally after meals. That post-prandial levels may be particularly pathophysiological for the endothelium is generally based around arguments surrounding 2-h OGTT post-challenge glucose concentrations rather than post-prandial levels. As post-challenge levels seem closely related to the features of the metabolic syndrome the argument for a special relationship to vascular damage is still limited, and the approach adopted in this document is simply to use the average relationship to basal glucose levels in people in good blood glucose control.

## Consideration

The intervention level/assessment level has been taken as a DCCT-aligned $HbA_{1c}$ of 6.5 %, with a target level less than that if easily achieved. This is taken as translating to basal self-

monitored plasma glucose levels <6.0 mmol/l (<110 mg/dl), with post-prandial target levels of <8.0 mmol/l (<145 mg/dl).

## Implementation

These targets should be incorporated in local protocols and guidelines detailing methods for evaluating and advising on lifestyle and pharmaceutical therapies as the natural history of the condition evolves.

## Evaluation

Glucose targets (as given above) should be present in local guidelines and protocols. Audit is of attained glucose control on different types of therapy.

## References


1. Stratton IM, Adler AI, Neil HA, Matthews DR, Manley SE, Cull CA, et al. Association of glycaemia with macrovascular and microvascular complications of type 2 diabetes (UKPDS 35): prospective observational study. BMJ 2000; 321: 405-12.
2. Laakso M, Kuusisto J. Epidemiological evidence for the association of hyperglycaemia and atherosclerotic vascular disease in non-insulin-dependent diabetes mellitus. Ann Med 1996; 28: 415-18.
3. Selvin E, Marinopoulos S, Berkenblit G, Rami T, Brancati FL, Powe NR, et al. Meta-analysis: glycosylated hemoglobin and cardiovascular disease in diabetes mellitus. Ann Intern Med 2004; 141: 421-31.
4. American Diabetes Association Position Statement. Standards of Medical Care in Diabetes. Diabetes Care 2005; 28(Suppl 1): S4-S36.
5. American Diabetes Association Position Statement. Tests of glycemia in diabetes. Diabetes Care 2004; 27(Suppl 1): S91-S93.
6. European Diabetes Policy Group 1999. A desktop guide to Type 2 diabetes mellitus. Diabet Med 1999; 16: 716-30. http://www.staff.ncl.ac.uk/philip.home/guidelines
7. McIntosh A, Hutchinson A, Home PD, Brown F, Bruce A, Damerell A, et al. Clinical guidelines and evidence review for Type 2 diabetes: management of blood glucose. Sheffield: ScHARR, University of Sheffield, 2001. http://www.nice.org.uk/pdf/NICE_full_blood_glucose.pdf
8. Canadian Diabetes Association Clinical Practice Guidelines Expert Committee. Canadian Diabetes Association 2003 Clinical Practice Guidelines for the Prevention and Management of Diabetes in Canada. Canadian Journal of Diabetes 2003; 27(Suppl 2): S18-S23. http://www.diabetes.ca
9. The National Collaborating Centre for Chronic Conditions. Type 1 Diabetes in Adults. National clinical guideline for diagnosis and management in primary and secondary care. http://www.rcplondon.ac.uk/pubs/books/DIA/index.asp
10. Khaw KT, Wareham N, Bingham S, Luben R, Welch A, Day N. Association of hemoglobin $A_{1c}$ with cardiovascular disease and mortality in adults: the European Prospective Investigation into Cancer in Norfolk. Ann Intern Med 2004; 141: 413-20.
11. Gray A, Raikou M, McGuire A, Fenn P, Stevens R, Cull C, et al. Cost effectiveness of an intensive blood glucose control policy in patients with type 2 diabetes: economic analysis alongside randomised controlled trial (UKPDS 41). BMJ 2000; 320: 1373-78.
12. Riddle M, Rosenstock J, Gerich J. The treat-to-target trial. Randomized addition of glargine or human NPH insulin to oral therapy of type 2 diabetic patients. Diabetes Care 2003; 26: 3080-86.
13. Nathan DM, Singer DE, Hurxthal K, Goodson JD. The clinical informational value of glycosylated hemoglobin assay. N Engl J Med 1984; 310: 341-46.
14. Rohlfing CL, Wiedmeyer H-M, Little RR, England JD, Tennill A, Goldstein DE. Defining the relationship between plasma glucose and $HbA_{1c}$. Diabetes Care 2002; 25: 275-78.

# Clinical monitoring

07

## Recommendations

### ■ Standard care

MO1 Monitor blood glucose control by high-precision methods of $HbA_{1c}$ performed every 2 to 6 months depending on level and stability of blood glucose control, and change in therapy.

MO2 Report all $HbA_{1c}$ results DCCT-aligned, pending internationally concerted policy changes.

MO3 Provide site-of-care measurement of $HbA_{1c}$, or laboratory measurement before clinical consultation.

MO4 Communicate the $HbA_{1c}$ result to the person with diabetes. The term 'A1c' may be useful in some populations.

MO5 Use appropriate alternative measures where $HbA_{1c}$ methods are invalidated by haemoglobinopathy or abnormal haemoglobin turnover.

MO6 Do not use fructosamine as a routine substitute for $HbA_{1c}$ measurement; it may be useful where $HbA_{1c}$ is not valid.

MO7 Site-of-care capillary plasma glucose monitoring at random times of day is not generally recommended.

### ■ Comprehensive care

$MO_C1$ This would be as for *Standard care*, but continuous glucose monitoring is an additional option in the assessment of glucose profiles in people with consistent glucose control problems, or with problems of $HbA_{1c}$ estimation.

$MO_C2$ $HbA_{1c}$ estimation would be available at each visit, and provided in electronic or paper diary form to the person with diabetes.

## ■ Minimal care

$MO_M1$ Fasting plasma glucose measurement could be used for monitoring.

$MO_M2$ Site-of-care capillary blood glucose meters should be quality controlled by reference to laboratory methods.

$MO_M3$ Visually read glucose test strips have a role in emergency and remote situations where maintenance of functional meters is not feasible.

## Rationale

Type 2 diabetes shows progression of hyperglycaemia with time, and causes organ damage through controllable hyperglycaemia. Accordingly hyperglycaemia has to be monitored. Some of this will be performed by the person with diabetes, some by site-of-care tests, and some by laboratory methods which can be referenced to studies of control and complications.

## Evidence-base

In general the major national guidelines do not address this area in detail. An exception is the 2004 NICE guideline for Type 1 diabetes [1]. This can be seen as applicable in terms of the methods proposed for clinic and office monitoring, and in particular for people using insulin therapy. Other guidelines and the ADA standards [2] do also centre on the $HbA_{1c}$ assay for clinic/office monitoring of glucose control, while laboratory guidelines address available methods and their quality implementation [3].

The central role for the $HbA_{1c}$ assay largely derives from its position in the reports of the major outcomes studies (the DCCT [4] and the UKPDS [5]). These provide the main method by which clinicians can relate individual blood glucose control to risk of complication development [6], and make $HbA_{1c}$ mandatory where affordable/available. The laboratory and site-of-care assays are precise and accurate if appropriately controlled and aligned with international standards. However, a number of issues still surround the results reported, including problems affecting haemoglobin itself (turnover or structural abnormalities [7]) and the absolute assay standard used. These issues in turn affect the recommendation to use HPLC-based assays where feasible, in order to detect haemoglobin variants. Additionally there are recommendations in the published guidelines on site-of-care testing, and on communication of the result to the person with diabetes.

Random clinic plasma glucose testing is not seen as having a role in quality diabetes care. Where $HbA_{1c}$ is unavailable, timed glucose levels are often recommended as a substitute (see also *Self-monitoring*). Recommendations are then made over the quality control of devices used to make such site-of-care tests. Continuous ambulatory blood glucose monitoring has become available in recent years. There is still no good evidence-base for its use, particularly in people with Type 2 diabetes.

## Consideration

The central role for site-of-care quality-controlled DCCT-aligned $HbA_{1c}$ testing was found to be solid. Blood glucose testing per se, using quality controlled methods, was noted to have a role in certain circumstances. The role of continuous monitoring remains to be established.

## Implementation

There should be access to a laboratory or site-of-care test that participates in a certified quality assurance scheme for measurement of $HbA_{1c}$. People for whom $HbA_{1c}$ measurement is inappropriate must be identified; HPLC can detect haemoglobinopathies. Organization to allow site-of-care or prior-to-visit sampling is also needed. Provision of capillary blood glucose meters and strips needs to be assured (if used). It is essential to establish whether meters report values for plasma or blood and to ensure that schemes for monitoring the quality of their output are in place.

## Evaluation

This is of the presence of records of $HbA_{1c}$ results in patient records, and documented evidence of the quality of performance of the assay system.

## References


1. The National Collaborating Centre for Chronic Conditions. Type 1 Diabetes in Adults. National clinical guideline for diagnosis and management in primary and secondary care. http://www.rcplondon.ac.uk/pubs/books/DIA/index.asp
2. American Diabetes Association Position Statement. Standards of Medical Care in Diabetes. Diabetes Care 2005; 28 (Suppl 1): S4-S36.
3. Sacks DB, Bruns DE, Goldstein DE, Maclaren NK, McDonald JM, Parrott M. Guidelines and recommendations for laboratory analysis in the diagnosis and management of diabetes mellitus. Diabetes Care 2002; 25: 750-86.
4. The Diabetes Control and Complications Trial Research Group. The relationship of glycemic exposure ($HbA_{1c}$) to the risk of development and progression of retinopathy in the Diabetes Control and Complications Trial. Diabetes 1995; 44: 968-83.
5. Stratton IM, Adler AI, Neil HA, Matthews DR, Manley SE, Cull CA, et al. Association of glycaemia with macrovascular and microvascular complications of type 2 diabetes (UKPDS 35): prospective observational study. BMJ 2000; 321: 405-12.
6. Manley S. Haemoglobin $A_{1c}$ – A marker for complications of type 2 diabetes. The experience from the UK Prospective Diabetes Study (UKPDS). Clin Chem Lab Med 2003; 41: 1182-90.
7. Sacks DB. Hemoglobin variants and hemoglobin $A_{1c}$ analysis: Problem solved? Clin Chem 2003; 49: 1245-47.

# Self-monitoring

08

## Recommendations

### ■ Standard care

SM1 Self-monitoring of blood glucose (SMBG) should be available for all newly diagnosed people with Type 2 diabetes, as an integral part of self-management education.

SM2 SMBG (using meter and strips) on an ongoing basis should be available to those on insulin treatment.

SM3 SMBG should be considered on an ongoing basis for people using oral agents, but not insulin, where it is used:

- to provide information on hypoglycaemia
- to assess glucose excursions due to medications and lifestyle changes
- to monitor changes during intercurrent illness.

SM4 SMBG should be considered on an intermittent basis for people not using insulin or oral agents, where it is used:

- to assess glucose excursions due to lifestyle changes
- to monitor changes during intercurrent illness.

SM5 Structured assessment of self-monitoring skills, the quality and use made of the results obtained, and of the equipment used, should be made annually.

### ■ Comprehensive care

$SM_C1$ This would be as *Standard care*, but SMBG (using meter and strips) on an ongoing basis could be offered to all people with Type 2 diabetes on insulin or oral agents.

### ■ Minimal care

$SM_M1$ SMBG using meters with strips, or visually read blood glucose strips, should be considered for those on insulin therapy.

## Rationale

Self-monitoring of glucose is widely used in the care plans of many people with Type 2 diabetes. It is often used to complement $HbA_{1c}$ measurement to assess blood glucose control and, in the case of self-monitoring of blood glucose (SMBG), provides real-time feedback of blood glucose levels. Its use can be considered in relation to:

- outcomes (a decrease in $HbA_{1c}$ with the ultimate aim of decreasing risk of complications)
- safety (identifying hypoglycaemia)
- process (education, self-empowerment, changes in therapy).

Self-monitoring should only be considered when the person with diabetes is prepared to learn the skill, record the findings, understand the data, and act appropriately on the data.

Urine glucose testing is cheap but has limitations. Urine free of glucose is an indication that the blood glucose level is below the renal threshold, which usually corresponds to a blood glucose level of about 10.0 mmol/l (180 mg/dl). Positive results do not distinguish between moderately and grossly elevated levels, and a negative result does not distinguish between normoglycaemia and hypoglycaemia.

## Evidence-base

The rather unsatisfactory evidence-base surrounding self-monitoring is addressed by guidelines from NICE [1,2] and the CDA [3]. Most of the evidence has focused on self-monitoring in relation to outcomes. Studies on self-monitoring in Type 2 diabetes were found to have been limited by small numbers, short duration, inconsistencies in monitoring and in the training of patients in technique or use of data, and failure to stratify by treatment type. A meta-analysis in 2000 found eight randomized trials, but no evidence for clinical effectiveness of this component of care [4]. A large observational study subsequently found evidence for improved glycaemic control with more frequent self-monitoring, regardless of therapy, but there was no stratification of new and ongoing users [5], and the NICE working group drew attention to the problem of separating out the effects of motivation in observational studies [1].

It is generally accepted that SMBG is useful in insulin-treated Type 2 diabetes [1,3,5]. Two recent meta-analyses of RCTs have examined its effect in people with Type 2 diabetes not treated with insulin [6,7]. Both showed that SMBG achieved a statistically significant reduction of 0.4 % in $HbA_{1c}$. However, it was acknowledged that the quality of the studies was limited and that a well designed RCT was needed to resolve this issue. Two accompanying point-of-view papers reached opposite conclusions about the value of SMBG [8,9].

There are many unresolved questions about SMBG, including frequency and timing of testing, its value in new users and ongoing users, and if and how users act on the results.

There are limited data on the impact of SMBG on quality of life and treatment satisfaction. From the two studies which reported on this [10,11], there was no difference compared with people who were not performing SMBG.

Also there are few data on self-monitoring using urine glucose testing. The meta-analysis by Welschen et al. [7] included two studies which compared SMBG and self-monitoring of urine glucose and reported a non-significant reduction in $HbA_{1c}$ of 0.17 % in favour of SMBG.

Two large cohort studies of self-monitoring of blood glucose in people with Type 2 diabetes, and including people not using insulin, have been submitted for publication at the time of writing (one presented at an ACE meeting in January 2005, and one presented as late-breaking data at the 2005 ADA Scientific Sessions). The data of these studies support the recommendations given above. However, a very recent publication addressing the same issue could not find such supportive evidence [12].

## Consideration

Self-monitoring of blood glucose is accepted as an integral part of self-management of people on insulin therapy. However, the data are less clear for people who are not being treated with insulin, and therefore the decision as to whether to recommend SMBG for this group will largely be determined by cost and individual and health-care system resources. Priority lists may be needed to decide which individuals should be offered SMBG on an ongoing basis. These might include people recently diagnosed with diabetes, with more erratic lifestyles, people having problems of hypoglycaemia, and those particularly keen to tighten their blood glucose control.

There is little evidence to support the use of urine testing. However, it should be noted that a recent IDF position statement has drawn attention to the fact that urine strips are cheap and that urine testing, although grossly inaccurate as a measure of blood glucose control, was used prior to the 1970s as the only means of self-monitoring, and could still be useful if its limitations are clearly understood [13].

## Implementation

Provision should be made for the supply of glucose strips on a continuing basis. When providing meters, education in their use and in interpretation of results from them should be given. Review of technique, data interpretation, and meter function should be a part of Annual Review (see *Care delivery*).

## Evaluation

Provision of self-monitoring education and equipment should be assessed, and protocols and a record of review as part of Annual Review should be available. There should be evidence of the results being made use of by the person with diabetes and in other clinical consultations with health-care professionals.

## References


1. McIntosh A, Hutchinson A, Home PD, Brown F, Bruce A, Damerell A, et al. Clinical guidelines and evidence review for Type 2 diabetes: management of blood glucose. Sheffield: ScHARR, University of Sheffield, 2001. http://www.nice.org.uk/pdf/NICE_full_blood_glucose.pdf
2. The National Collaborating Centre for Chronic Conditions. Type 1 Diabetes in Adults. National clinical guideline for diagnosis and management in primary and secondary care. http://www.rcplondon.ac.uk/pubs/books/DIA/index.asp
3. Canadian Diabetes Association Clinical Practice Guidelines Expert Committee. Canadian Diabetes Association 2003 Clinical Practice Guidelines for the Prevention and Management of Diabetes in Canada. Canadian Journal of Diabetes 2003; 27(Suppl 2): S18-S23. http://www.diabetes.ca
4. Coster S, Gulliford MC, Seed PT, Powrie JK, Swaminathan R. Self-monitoring in Type 2 diabetes: a meta-analysis. Diabet Med 2000; 17: 755-61.
5. Karter AJ, Ackerson LM, Darbinian JA, D'Agostino Jr RB, Ferrara A, Liu J, et al. Self-monitoring of blood glucose levels and glycemic control: the Northern California Kaiser Permanente Diabetes Registry. Am J Med 2001; 111: 1-9.
6. Sarol JN, Nicodemus NA, Tan KM, Grava MB. Self-monitoring of blood glucose as part of a multi-component therapy among non-insulin requiring type 2 diabetes patients: a meta-analysis (1966-2004). Current Medical Research and Opinion 2005; 21: 173-83.
7. Welschen LMC, Bloemendal E, Nijpels G, Dekker JM, Heine RJ, Stalman WAB, et al. Self-monitoring of blood glucose in patients with type 2 diabetes who are not using insulin: a systematic review. Diabetes Care 2005; 28: 1510-17.
8. Ipp E, Aquino RL, Christenson P. Point: Self-monitoring of blood glucose in type 2 diabetic patients not receiving insulin: the sanguine approach. Diabetes Care 2005; 28: 1528-30.
9. Davidson MB. Counterpoint: Self-monitoring of blood glucose in type 2 diabetic patients not receiving insulin: a waste of money. Diabetes Care 2005; 28: 1531-33.
10. Muchmore DB, Springer J, Miller M. Self-monitoring of blood glucose in overweight type 2 diabetic patients. Acta Diabetol 1994; 31: 215-19.
11. Schwedes U, Siebolds M, Mertes G for the SMBG Study Group. Meal-related structured self-monitoring of blood glucose. Effect on diabetes control in non-insulin-treated type 2 diabetic patients. Diabetes Care 2002; 25: 1928-32.
12. Franciosi M, Pellegrini F, De Berardis G, Belfiglio M, Di Nardo B, Greenfield S, et al. Self-monitoring of blood glucose in non-insulin-treated diabetic patients: a longitudinal evaluation of its impact on metabolic control. Diabet Med 2005; 22; 900-06.
13. IDF position statement. The role of urine glucose monitoring in diabetes. March 2005. http://www.idf.org

# Glucose control: oral therapy

09

## Recommendations

### ■ Standard care

OA1 Begin oral glucose-lowering drugs when lifestyle interventions alone are unable to maintain blood glucose control at target levels (see *Glucose control levels*).

Maintain support for lifestyle measures throughout the periods of use of these drugs.

Consider each initiation or dose increase of an oral glucose-lowering drug as a trial, monitoring the response in 2-6 months.

OA2 Begin with metformin unless evidence or risk of renal impairment, titrating the dose over early weeks to minimize discontinuation due to gastro-intestinal intolerance.

Monitor renal function and risk of significant renal impairment (eGFR <60 ml/min/ 1.73 $m^2$) in people taking metformin.

OA3 Use sulfonylureas when metformin fails to control glucose concentrations to target levels, or as a first-line option in the person who is not overweight.

Choose a drug of low cost, but exercise caution if hypoglycaemia may be a problem to the individual, including through renal impairment.

Provide education and, if appropriate, self-monitoring (see *Self-monitoring*) to guard against the consequences of hypoglycaemia.

Once-daily sulfonylureas should be an available option where drug concordance is problematic.

Rapid-acting insulin secretagogues may be useful as an alternative to sulfonylureas in some insulin-sensitive people with flexible lifestyles.

OA4 Use a PPAR-γ agonist (thiazolidinedione) when glucose concentrations are not controlled to target levels, adding it:

- to metformin as an alternative to a sulfonylurea, or
- to a sulfonylurea where metformin is not tolerated, or
- to the combination of metformin and a sulfonylurea.

Be alert to the contra-indication of cardiac failure, and warn the person with diabetes of the possibility of development of significant oedema.

OA5 Use α-glucosidase inhibitors as a further option. They may also have a role in some people intolerant of other therapies.

OA6 Step up doses, and add other oral glucose-lowering drugs, at frequent intervals until blood glucose control is at target levels. Consider whether the rate of deterioration suggests insulin therapy will be needed early despite such measures.

## Comprehensive care

$OA_C1$ The principles of use of oral glucose-lowering drugs are as for *Standard care*. Metformin remains the drug of choice for first-line therapy.

## Minimal care

$OA_M1$ Metformin and a generic sulfonylurea should be the basis of oral glucose-lowering therapy. Where the costs of thiazolidinedione therapy are lower than those of basic insulin therapy, use of these drugs may be considered before transfer to insulin.

$OA_M2$ Where renal function tests are not routinely available for people on metformin, such tests are nevertheless required where the likelihood of renal impairment is high.

## Rationale

The evidence that elevated blood glucose levels can result in various forms of vascular damage is discussed elsewhere in this guideline (see *Glucose control levels*). Lifestyle modification (see *Lifestyle management*) by itself can only provide control of blood glucose concentrations to safe target levels in a minority of people with diabetes, and then usually only for a limited period after diagnosis. Accordingly, supplementary pharmaceutical measures are needed, and these can be oral glucose-lowering drugs and insulin injection therapy, separately or in combination.

## Evidence-base

A number of systematic evidence-based reviews addressing oral glucose-lowering drugs have been published in recent years [1-4]. These nearly always use the UKPDS as the basis of a conclusion that glucose lowering with oral drugs is effective in protection against vascular complications [5]. They also conclude that the evidence on better prevention of arterial outcomes when using metformin in the overweight sub-study of UKPDS [6] supports the primary use of that drug in all overweight people with Type 2 diabetes, and indeed probably in all people with Type 2 diabetes.

The reviews note that UKPDS in particular confirms that hyperglycaemia in people with diabetes is a progressive condition due to progressive islet B-cell failure, and thus requires continued monitoring and stepping up of therapies to maintain glucose control targets. The NICE guideline [2] notes the problem of concordance with multiple therapies (particularly as people will often be on blood-pressure-lowering, lipid-lowering, and cardiovascular medications), and suggests once-daily drugs may have advantage in many circumstances.

Review of effectiveness of glucose lowering concludes that the drugs from different classes are generally similar, except that α-glucosidase inhibitors may be less efficacious than sulfonylureas [1,2,7]. Other evidence suggests that nateglinide, a rapid-acting insulin secretagogue, is also less efficacious in this regard.

The two available PPAR-γ agonists (thiazolidinediones), while as effective as metformin and sulfonylurea in lowering glucose levels, are found to have other positive effects on risk factors associated with cardiovascular disease, but mixed effects on lipoproteins [8-10]. The former include improvements in vascular inflammation, albumin excretion rate, blood pressure, endothelial and clotting factors, and insulin insensitivity. At the time of review, no studies have confirmed that these effects give beneficial health outcomes, but some of the effects are qualitatively similar in nature, but quantitatively greater, than are found with metformin. Systematic reviews of the α-glucosidase inhibitors have not found reason to recommend them over less expensive and better tolerated drugs [1,2,7].

Lactic acidosis is a rare complication (often fatal) of metformin therapy in people with renal impairment. Gastro-intestinal intolerance of this drug is very common, particularly at higher dose levels and with fast upward dose titration. Some sulfonylureas, notably glyburide, are known to be associated with severe hypoglycaemia and rarely death from this, again usually in association with renal impairment. Thiazolidinediones can cause fluid retention and are contra-indicated in the presence of higher grades of heart failure [11].

Generic metformin and sulfonylureas are available at very low cost. Proprietary oral glucose-lowering drugs are considerably more expensive, with limited evidence of extra benefit. Thiazolidinediones are relatively new drugs and are also usually expensive.

## Consideration

The outcome-based evidence from the UKPDS for the use of metformin in overweight people with Type 2 diabetes, exceeding that for any other drug, leads to its recommendation for first-line use, although the sulfonylureas also protected against vascular damage in that study. Cheap generic versions of these drugs are available, and their glucose-lowering capacity is not surpassed by any newer drug, at least on a population basis. However, tolerance and safety issues are of concern with metformin, the latter particularly if renal impairment is present. Concern over hypoglycaemia with some of the sulfonylureas is also felt to be of significance, especially with renal impairment. The evidence on the thiazolidinediones, effective in glucose-lowering and in having positive effects on some cardiovascular risk markers, would now seem to justify an early role for these drugs in combination oral agent therapy. However, they remain relatively expensive in most health-care markets.

Combination of oral glucose-lowering drugs with insulin therapy is discussed below (see *Insulin therapy*).

## Implementation

Contracts should be in place for uninterrupted availability of at least one sulfonylurea, metformin and (for standard/comprehensive care) at least one thiazolidinedione. Availability is needed of an $HbA_{1c}$ assay and visits to health-care professionals at a frequency (sometimes 3-monthly) sufficient to titrate therapy where glucose control is deteriorating. Lifestyle measures, self-monitoring where appropriate, and education, as discussed elsewhere in this guideline, are integral parts of maintaining glucose control to target, and will enhance the effectiveness of oral drugs. The recommendations should be a basis of local clinical protocols and structured records.

## Evaluation

Evaluation of achieved blood glucose control should be by reference to the documented use of oral therapies and insulin in different combinations to identify appropriately early use of these drugs, and in the appropriate order. Reference to measures of renal and cardiac failure may be used to identify use where contra-indications apply. Local protocols should be identifiable.

## References


1. Canadian Diabetes Association Clinical Practice Guidelines Expert Committee. Canadian Diabetes Association 2003 Clinical Practice Guidelines for the Prevention and Management of Diabetes in Canada. Canadian Journal of Diabetes 2003; 27(Suppl 2): S37-S42. http://www.diabetes.ca
2. McIntosh A, Hutchinson A, Home PD, Brown F, Bruce A, Damerell A, et al. Clinical guidelines and evidence review for Type 2 diabetes: management of blood glucose. Sheffield: ScHARR, University of Sheffield, 2001. http://www.nice.org.uk/pdf/NICE_full_blood_glucose.pdf
3. Häring HU, Joost HG, Laube H, Matthaei S, Meissner HP, Panten U, et al. Antihyperglykämische Therapie des Diabetes mellitus Typ 2. In: Scherbaum WA, Landgraf R (eds) Evidenzbasierte Diabetes-Leitlinie DDG. Diabetes und Stoffwechsel 2003; 12 (Suppl 2). http://www.deutsche-diabetes-gesellschaft.de (updated 2004)
4. Institute for Clinical Systems Improvement (Bloomington, MN, USA). Management of Type 2 Diabetes Mellitus, 2004. http://www.icsi.org/knowledge
5. UK Prospective Diabetes Study (UKPDS) Group. Intensive blood-glucose control with sulphonylureas or

insulin compared with conventional treatment and risk of complications in patients with type 2 diabetes (UKPDS 33). Lancet 1998; 352: 837-53.
6. UK Prospective Diabetes Study (UKPDS) Group. Effect of intensive blood-glucose control with metformin on complications in overweight patients with type 2 diabetes (UKPDS 34). Lancet 1998; 352: 854-65.
7. Van de Laar FA, Lucassen PL, Akkermans RP, Van de Lisdonk EH, Rutten GE, Van Weel C. α-Glucosidase inhibitors for patients with type 2 diabetes: results from a Cochrane systematic review and meta-analysis. Diabetes Care 2005; 28: 154-63.
8. Zimmet P. Addressing the insulin resistance syndrome. A role for the thiazolidinediones. Trends Cardiovasc Med 2002; 12: 354-62.
9. Viberti G. Rosiglitazone: potential beneficial impact on cardiovascular disease. Int J Clin Pract 2003; 57: 128-34.
10. Nesto R. C-reactive protein, its role in inflammation, Type 2 diabetes and cardiovascular disease, and the effects of insulin-sensitizing treatment with thiazolidinediones. Diabet Med 2004; 21: 810-17.
11. Nesto RW, Bell D, Bonow RO, Fonseca V, Grundy SM, Horton ES, et al. Thiazolidinedione use, fluid retention, and congestive heart failure. A consensus statement from the American Heart Association and American Diabetes Association. Diabetes Care 2004; 27: 256-63.

# Glucose control: insulin therapy

10

## Recommendations

### ■ Standard care

IN1 Begin insulin therapy when optimized oral glucose-lowering drugs and lifestyle interventions are unable to maintain blood glucose control at target levels (see *Glucose control levels*).

Maintain support for lifestyle measures after introduction of insulin.

Consider every initiation or dose increase of insulin as a trial, monitoring the response.

IN2 Explain to the person with diabetes from the time of diagnosis that insulin is one of the options available to aid management of their diabetes, and that it may turn out to be the best, and eventually necessary, way of maintaining blood glucose control, especially in the longer term.

IN3 Provide education, including on continuing lifestyle management (see *Education, Lifestyle management*), and appropriate self-monitoring (see *Self-monitoring*).

Explain that starting doses of insulin are low, for safety reasons, but that eventual dose requirement is expected to be 50-100 units/day.

Initiate insulin therapy before poor glucose control develops, generally when DCCT-aligned $HbA_{1c}$ has deteriorated to >7.5 % (confirmed) on maximal oral agents.

Continue metformin. Additionally continue sulfonylureas when starting basal insulin therapy. $\alpha$-Glucosidase inhibitors may also be continued.

IN4 Use:

- a basal insulin once daily such as insulin detemir, insulin glargine, or NPH insulin (risk of hypoglycaemia is higher with the last), or
- twice daily premix insulin (biphasic insulin) particularly with higher $HbA_{1c}$, or
- multiple daily injections (meal-time and basal insulin) where blood glucose control is sub-optimal on other regimens, or meal-time flexibility is desired.

IN5 Initiate insulin using a self-titration regimen (dose increases of 2 units every 3 days) or by weekly or more frequent contact with a health-care professional (using a scaled algorithm).

Aim for pre-breakfast *and* pre-main-evening-meal glucose levels of <6.0 mmol/l (<110 mg/dl); where these seem not to be achievable use monitoring at other times to identify the profile of poor glucose control.

IN6 Continue health-care professional support by telephone until target levels (see *Glucose control levels*) are achieved.

IN7 Use pen-injectors (prefilled or re-usable) or syringes/vials according to choice of the person using them.

IN8 Encourage subcutaneous insulin injection into the abdominal area (most rapid absorption) or thigh (slowest), with the gluteal area (or the arm) as other possible injection sites. Bear in mind that reluctance to use the abdominal region may relate to cultural background.

## Comprehensive care

$IN_C1$ The principles of insulin use are as for *Standard care*.

$IN_C2$ Insulin analogues would generally be used.

$IN_C3$ Where permitted and appropriate, combination use of insulin and a PPAR-γ agonist is an option, with cautions over cardiac failure.

$IN_C4$ Insulin pump therapy may be an additional option.

## Minimal care

$IN_M1$ The principles of insulin use, including professional support, are as for *Standard care*. Self-monitoring may be limited to pre-breakfast and pre-evening-meal.

$IN_M2$ Use a combination of an oral glucose-lowering drug (usually metformin) with NPH insulin twice daily (or once daily if initiated early), or twice-daily insulin mixes.

$IN_M3$ The supplied insulin should be of assured and consistent quality and type.

$IN_M4$ Use insulin syringes and vials.

## Rationale

The rationale for the use of glucose-lowering therapy titrated to blood glucose targets is given in the section on oral agents. The natural history of Type 2 diabetes is of progression of islet B-cell failure – insulin remains the only glucose-lowering therapy which can maintain blood glucose control despite such progression.

## Evidence-base

The evidence-based guidelines addressing insulin use in Type 2 diabetes [1-3] draw on the evidence from UKPDS that insulin was among the glucose-lowering therapies which, considered together, reduced vascular complications compared with 'conventional' therapy [4]. The options for insulin therapy (preparations, delivery) have expanded

considerably since the UKPDS. The NICE evidence review found that studies on older preparations tended to be less highly rated for quality, while evidence for the newer insulin analogues was still emerging [1]. The more recent Canadian guidelines found indications for use of analogues in relation to postprandial glucose excursions, risk of hypoglycaemia, and weight gain [2]. A recent meta-analysis found good evidence of less hypoglycaemia with insulin glargine compared with NPH insulin [5]. Insulin glargine was the subject of specific guidance from NICE [6] including a recommendation for use where once-daily injections would suffice or NPH insulin gave troublesome hypoglycaemia. Other studies with insulin analogues or comparing basal analogues and analogue premixes have since appeared [7,8]. These suggest that basal analogues have advantage over NPH insulin for combined endpoints ($HbA_{1c}$ + hypoglycaemia), while there is a balance of advantage between biphasic analogues and basal analogues when $HbA_{1c}$, hypoglycaemia and weight gain are considered together. Risk, and hence fear, of hypoglycaemia is greater with insulin than with any of the insulin secretagogues.

There is supporting evidence for insulin use in combination with metformin, insulin secretagogues (sulfonylureas), metformin plus sulfonylurea (no meta-analysis), α-glucosidase inhibitors, thiazolidinediones [2,9]. The NICE review found that for people on insulin therapy, glucose control was improved and body weight and hypoglycaemia risk reduced when metformin was used in combination; the evidence that blood glucose control was improved when sulfonylureas were taken concomitantly with insulin was not conclusive [1]. Uncontrolled observations since that review support the hypothesis, notably in combination with basal insulin therapy [10]. Major outcome studies are not yet available for the combination of insulin with rapid-acting insulin secretagogues or thiazolidinediones.

A 2005 Cochrane review including 45 RCTs with 2156 participants found no differences in metabolic control or hypoglycaemic episodes between human insulin and animal insulin [11], although patient-oriented outcomes like quality of life, diabetes complications and mortality were not suitably addressed by high-quality RCTs. Although cost-effectiveness currently favours non-human insulin, this situation is changing.

Rapid-acting insulin analogues were the subject of a recent Cochrane review, which had some methodological weaknesses [12]. Modest benefits were found for the analogues, which might be considered for patients using rather more intensified regimens or with more advanced insulin deficiency.

Intensified insulin therapy in Type 2 diabetes has been shown to improve metabolic control, improve clinical outcomes [13], and increase flexibility. Evidence on pump therapy in Type 2 diabetes is still insufficient to support a recommendation for use in general, although it is a potential option in highly selected patients or in very individual settings [14].

## Consideration

The evidence shows that a DCCT-aligned $HbA_{1c}$ level of around 7.0 % (population mean) is achievable with insulin therapy in combination with oral glucose-lowering drugs, provided insulin deficiency has not progressed too far. This suggests it is worthwhile starting when control has deteriorated to >7.5 %. Active titration of dosage by self-monitoring and continued educational support is needed to achieve this. It is well recognized that personal preferences have a major role to play in the use of insulin. Long-acting analogue studies show less hypoglycaemia compared with NPH insulin. However, the evidence suggests that active use of combination oral agents is necessary in many people to maintain glucose control throughout the day, and that meal-time insulin (as biphasic preparations or with meal-time supplements) becomes necessary with time.

Insulin analogues can be expensive. Where this is an issue, NPH insulin and human insulin mixes are still very useful alternatives. However, consistency of supply (quality, availability, insulin type) requires careful organization.

## Implementation

Contracts should be in place for uninterrupted availability of insulin and supporting materials (including for self-monitoring and education).

Availability of an $HbA_{1c}$ assay (except in *Minimal care*), and of health-care professionals for education and advice at high intensity when titrating doses, needs to be assured.

Avoiding delay in starting insulin therapy has been problematic in nearly all diabetes services. Structured guidelines and protocols and audit of glucose control of people on oral drugs appear to be an integral part of dealing with this problem.

## Evaluation

Evaluation should be of achieved blood glucose control of people on oral drugs and those started on insulin therapy, with reference to the documented use of those therapies once insulin has been started. Local protocols and resources should be identifiable.

## References

1. McIntosh A, Hutchinson A, Home PD, Brown F, Bruce A, Damerell A, et al. Clinical guidelines and evidence review for Type 2 diabetes: management of blood glucose. Sheffield: ScHARR, University of Sheffield, 2001. http://www.nice.org.uk/pdf/NICE_full_blood_glucose.pdf
2. Canadian Diabetes Association Clinical Practice Guidelines Expert Committee. Canadian Diabetes Association 2003 Clinical Practice Guidelines for the Prevention and Management of Diabetes in Canada. Canadian Journal of Diabetes 2003; 27(Suppl 2): S37-S42. http://www.diabetes.ca
3. Häring HU, Joost HG, Laube H, Matthaei S, Meissner HP, Panten U, et al. Antihyperglykämische Therapie des Diabetes mellitus Typ 2. In: Scherbaum WA, Landgraf R (eds) Evidenzbasierte Diabetes-Leitlinie DDG. Diabetes und Stoffwechsel 2003; 12 (Suppl 2). http://www.deutsche-diabetes-gesellschaft.de (updated 2004)
4. UK Prospective Diabetes Study (UKPDS) Group. Intensive blood-glucose control with sulphonylureas or insulin compared with conventional treatment and risk of complications in patients with type 2 diabetes (UKPDS 33). Lancet 1998; 352: 837-53.
5. Rosenstock J, Dailey G, Massi-Benedetti M, Fritsche A, Lin Z, Salzman A. Reduced hypoglycemia risk with insulin glargine. A meta-analysis comparing insulin glargine with human NPH insulin in type 2 diabetes. Diabetes Care 2005; 28: 950-55.
6. National Institute for Clinical Excellence. Guidance on the use of long-acting insulin analogues for the treatment of diabetes - insulin glargine. NICE Technology Appraisal Guidance No. 53. London: National Institute for Clinical Excellence, 2002. http://www.nice.org.uk
7. Riddle M, Rosenstock J, Gerich J. The treat-to-target trial. Randomized addition of glargine or human NPH insulin to oral therapy of type 2 diabetic patients. Diabetes Care 2003; 26: 3080-86.
8. Raskin P, Allen E, Hollander P, Lewin A, Gabbay RA, Hu P, et al. Initiating insulin therapy in type 2 diabetes: a comparison of biphasic and basal insulin analogs. Diabetes Care 2005; 28: 260-65.
9. Goudswaard AN, Furlong NJ, Valk GD, Stolk RP, Rutten GEHM. Insulin monotherapy versus combinations of insulin with oral hypoglycaemic agents in patients with type 2 diabetes mellitus. The Cochrane Database of Systematic Reviews 2004, Issue 4. Art. No.: CD003418.pub2. DOI: 10.1002/14651858.CD003418.pub2.
10. Janka HU, Plewe G, Riddle MC, Kliebe-Frisch C, Schweitzer MA, Yki-Järvinen H. Comparison of basal insulin added to oral agents versus twice-daily premixed insulin as initial insulin therapy for type 2 diabetes. Diabetes Care 2005; 28: 254-59.
11. Richter B, Neises G. 'Human' insulin versus animal insulin in people with diabetes mellitus. The Cochrane Database of Systematic Reviews 2005, Issue 1. Art. No.: CD003816.pub2. DOI: 10.1002/14651858.CD003816.pub2.
12. Siebenhofer A, Plank J, Berghold A, Narath M, Gfrerer R, Pieber TR. Short acting insulin analogues versus regular human insulin in patients with diabetes mellitus. The Cochrane Database of Systematic Reviews 2004, Issue 4. Art. No.: CD003287.pub3. DOI: 10.1002/14651858.CD003287.pub3.
13. Ohkubo Y, Kishikawa H, Araki E, Miyata T, Isami S, Motoyoshi S, et al. Intensive insulin therapy prevents the progression of diabetic microvascular complications in Japanese patients with non-insulin-dependent diabetes mellitus: a randomized prospective 6-year study. Diabetes Res Clin Pract 1995; 28: 103-17.
14. Raskin P, Bode BW, Marks JB, Hirsch IB, Weinstein RL, McGill JB, et al. Continuous subcutaneous insulin therapy and multiple daily injection therapy are equally effective in type 2 diabetes. Diabetes Care 2003; 26: 2598-603.

# Blood pressure control

11

## Recommendations

### Standard care

BP1 Measure blood pressure annually, and at every routine clinic visit if found to be above target levels (see below), or if on treatment:
- use a mercury sphygmomanometer or validated meter in good working order and an appropriately sized cuff (large or normal depending on arm size)
- measure after sitting for at least 5 min, with arm at heart level, using first and fifth phases of Korotkoff sounds
- record all values in a record card held by the person with diabetes
- use 24-hour ambulatory monitoring (ABPM) if 'white coat' hypertension suspected, but adjust targets down by 10/5 mmHg.

BP2 Consider secondary causes of raised blood pressure if there is evidence of renal disease, electrolyte disturbance or other features.

BP3 Aim to maintain blood pressure below 130/80 mmHg (for people with raised albumin excretion rate see *Kidney damage*).

Add further drugs if targets are not reached on maximal doses of current drugs, reviewing the preferences and beliefs of the individual concerned, and likely adherence problems as tablet numbers increase.

Accept that even 140/80 mmHg may not be achievable with 3 to 5 anti-hypertensive drugs in some people.

Revise individual targets upwards if there is significant risk of postural hypotension and falls.

BP4 Initiate a trial of lifestyle modification alone with appropriate education for 3 months (see *Lifestyle management*), aiming to reduce calorie intake, salt intake, alcohol intake, and inactivity.

BP5 Initiate medication for lowering blood pressure in diabetes not complicated by raised albumin excretion rate, using any agent except for $\alpha$-adrenergic blockers, with consideration of costs, and actively titrating dose according to response:
- ACE-inhibitors and A2RBs may offer some advantages over other agents in some situations (see *Kidney damage, Cardiovascular risk protection*), but are less effective in people of African extraction
- start with $\beta$-adrenergic blockers in people with angina, $\beta$-adrenergic blockers or ACE-inhibitors in people with previous myocardial infarction, ACE-inhibitors or diuretics in those with heart failure
- care should be taken with combined thiazide and $\beta$-adrenergic blockers because of risk of deterioration in metabolic control.

## Comprehensive care

$BP_C1$ This will in general be as for *Standard care*, but with the additional option of self-monitoring of blood pressure on validated semi-automatic devices to provide additional information and educational feedback.

## Minimal care

$BP_M1$ Measurement and targets will be as for *Standard care*.

$BP_M2$ Initiate a trial of lifestyle modification (as *Standard care*) with appropriate education (see *Lifestyle management*).

$BP_M3$ Initiate medication for lowering blood pressure in diabetes not complicated by proteinuria, using generic diuretics, β-adrenergic blockers, calcium channel blockers, or ACE-inhibitors as available, increasing the number of preparations used according to drug availability locally.

## Rationale

Blood pressure is elevated in many people with Type 2 diabetes. Increasing blood pressure levels are associated with a spectrum of later health problems in people with diabetes, notably cardiovascular disease (especially stroke), eye damage and kidney damage.

## Evidence-base

Review of the evidence-base on this topic is spread among guidelines primarily addressing diabetes [1-4] or hypertension [5,6], often embedded in consideration of cardiovascular disease [7] or kidney disease (see *Kidney damage*). The evidence may derive from trials involving primarily people with diabetes [8] or people with hypertension [9].

Recommendations on thresholds for intervention and targets of therapy vary narrowly across the guidelines. Some of this variation reflects concern at setting targets that are difficult to achieve in some people, and may appear unduly daunting, especially when many drugs are required. In the UKPDS, beneficial effects on complications, in particular stroke and retinopathy, were achieved at 144/82 mmHg in the tighter control group [8], consistent with results from the HOT study [9]. However, epidemiological analysis of UKPDS suggested benefits well below this level, supported by achievement of blood pressure down to 128/75 mmHg in other studies [1]. The recommended target of <130/80 mmHg for people with Type 2 diabetes uncomplicated by nephropathy is in line with the more recent guidelines [1-3,5,6].

Evidence on methods for measuring blood pressure was reviewed by the Australian guideline [1]. A meta-analysis of use of self-monitoring of blood pressure found it resulted in a small but statistically significant reduction [10]. Lifestyle modification (including weight reduction, reducing salt intake, increasing physical activity, reducing alcohol intake) can reduce systolic blood pressure by 4-10 mmHg (see *Lifestyle management*).

Many randomized trials have shown that blood-pressure-lowering therapy reduces cardiovascular disease morbidity and mortality in people with diabetes. Many agents (ACE-inhibitors, β-adrenergic blockers and low-dose thiazide diuretics) have proved effective. Choice of agent for a person with diabetes may be influenced by a number of factors including their risk profile (cardiovascular, renal, end-organ damage), preferences, and previous experience of therapy, as well as costs. Thiazide diuretics may adversely affect glucose, lipid and potassium levels, and β-adrenergic blockers may adversely affect glucose and lipid levels, but no RCTs have shown these drugs to increase cardiovascular mortality in Type 2 diabetes [1]. Avoidance of α-adrenergic blockers as first-line therapy is based on evidence from ALLHAT [2].

Cost issues, and particularly the data from UKPDS [11], were considered in the Australian guideline [1], which concluded that controlling blood pressure in people with Type 2 diabetes is cost-effective.

Achieving effective control of blood pressure, and consequent therapeutic benefits, is reported to depend

on adherence to therapy. Cultural health beliefs, complex therapeutic regimens, adverse effects, tablet number burden, and poor social support are reported predictors of poor concordance with therapy. These issues need to be discussed with the person concerned, where response to drugs is poor.

## Consideration

Blood pressure management appears to be among the most cost-effective methods of prevention of vascular complications in people with Type 2 diabetes. Lifestyle measures are generally preferred as a trial before therapeutic intervention, but alone are generally insufficient. Because individual therapies are not particularly effective even in full dosage, the experience of the need for multiple therapies found in UKPDS is reflected in the guideline recommendations. However, this also implies the need for frequent monitoring and dose titration until targets, or the limits of therapeutic effect, are reached.

## Implementation

There is need for equipment for measurement of blood pressure, maintenance of that equipment, and training of personnel in its use. Protocols using locally available drugs should be drawn up and followed to ensure drug prescription, and dose titration to target. Lifestyle education is described elsewhere (see *Lifestyle management*).

## Evaluation

A record of measurement of blood pressure within clinical records in the last 12 months should be found. Where that is elevated there should be evidence of action to lower it. The percentage of people in whom blood pressure achieves the target level 130/80 mmHg can be ascertained, and the percentage of those with blood pressure above target who are receiving treatment involving lifestyle modification and drug therapy. Availability of sphygmomanometers in working order, and appropriate cuffs can be ascertained, as can training and proficiency of staff measuring blood pressure.

## References


1. Jerums G, Colagiuri S, Panagiotopoulos S, Meng C, Colagiuri R. Evidence Based Guidelines for Type 2 Diabetes: Blood pressure control. Canberra: Diabetes Australia & NHMRC, 2004. http://www.diabetesaustralia.com.au
2. Canadian Diabetes Association Clinical Practice Guidelines Expert Committee. Canadian Diabetes Association 2003 Clinical Practice Guidelines for the Prevention and Management of Diabetes in Canada. Canadian Journal of Diabetes 2003; 27(Suppl 2): S113-S116. http://www.diabetes.ca
3. American Diabetes Association Position Statement. Standards of Medical Care in Diabetes. Diabetes Care 2005; 28(Suppl 1): S4-S36.
4. The National Collaborating Centre for Chronic Conditions. Type 1 Diabetes in Adults. National clinical guideline for diagnosis and management in primary and secondary care. http://www.rcplondon.ac.uk/pubs/books/DIA/index.asp
5. Chobanian AV, Bakris GL, Black HR, Cushman WC, Green LA, Izzo Jr JL, et al. National Heart, Lung, and Blood Institute Joint National Committee on Prevention, Detection, Evaluation, and Treatment of High Blood Pressure. National High Blood Pressure Education Program Coordinating Committee. The Seventh Report of the Joint National Committee on Prevention, Detection, Evaluation, and Treatment of High Blood Pressure: the JNC 7 report. JAMA 2003; 289: 2560-72.
6. European Society of Hypertension – European Society of Cardiology. 2003 European Society of Hypertension – European Society of Cardiology guidelines for the management of arterial hypertension. J Hypertens 2003; 21: 1011-53. http://www.eshonline.org/documents/2003_guidelines.pdf
7. Hutchinson A, McIntosh A, Griffiths CJ, Amiel S, Bilous R, Chaturvedi N, et al. Clinical guidelines and evidence review for Type 2 diabetes. Blood pressure management. Sheffield: ScHARR, University of Sheffield, 2002. http://www.nice.org.uk/pdf/bloodpressure_full_guideline.pdf
8. UK Prospective Diabetes Study Group. Tight blood pressure control and risk of macrovascular and microvascular complications in type 2 diabetes: UKPDS 38. BMJ 1998; 317: 703-13.
9. Hansson L, Zanchetti A, Carruthers SG, Dahlof D, Julius S, Menard J, et al. Effects of intensive blood-pressure lowering and low-dose aspirin in patients with hypertension: principal results of the Hypertension Optimal Treatment (HOT) randomised trial. Lancet 1998; 351: 1755-62.
10. Cappuccio FP, Kerry SM, Forbes L, Donald A. Blood pressure control by home monitoring: meta-analysis of randomised trials. BMJ 2004; 329: 145-48.
11. UK Prospective Diabetes Study Group. Cost effectiveness analysis of improved blood pressure control in hypertensive patients with type 2 diabetes: UKPDS 40. BMJ 1998; 317: 720-26.

# Cardiovascular risk protection

12

Cardiovascular risk protection through blood glucose control, blood pressure control, and lifestyle interventions is dealt with elsewhere in this guideline (see *Glucose control*, *Blood pressure control*, *Lifestyle management*). This section deals with cardiovascular risk assessment, lipid modifying therapy, and anti-platelet therapy.

## Recommendations

### ■ Standard care

CV1 Assess cardiovascular risk at diagnosis and at least annually thereafter:

- current or previous cardiovascular disease (CVD)
- age and BMI (abdominal adiposity)
- conventional cardiovascular (CV) risk factors including smoking and serum lipids, and family history of premature CVD
- other features of the metabolic syndrome and renal damage (including low HDL cholesterol, high triglycerides, raised albumin excretion rate)
- atrial fibrillation (for stroke).

Do not use risk equations developed for non-diabetic populations. The UKPDS risk engine may be used for assessment and communication of risk.

CV2 Ensure optimal management through lifestyle measures (see *Lifestyle management*), and measures directed at good blood glucose and blood pressure control (see *Glucose control, Blood pressure control*).

CV3 Arrange smoking cessation advice in smokers contemplative of reducing or stopping tobacco consumption.

CV4 Provide aspirin 75-100 mg daily (unless aspirin intolerant or blood pressure uncontrolled) in people with evidence of CVD or at high risk.

CV5 Provide active management of the blood lipid profile:

- a statin at standard dose for all >40 yr old (or all with declared CVD)
- a statin at standard dose for all >20 yr old with microalbuminuria or assessed as being at particularly high risk
- in addition to statin, fenofibrate where serum triglycerides are >2.3 mmol/l (>200 mg/dl), once LDL cholesterol is as optimally controlled as possible
- consideration of other lipid-lowering drugs (ezetimibe, sustained release nicotinic acid, concentrated omega 3 fatty acids) in those failing to reach lipid-lowering targets or intolerant of conventional drugs.

Reassess at all routine clinical contacts to review achievement of lipid targets: LDL cholesterol <2.5 mmol/l (<95 mg/dl), triglyceride <2.3 mmol/l (<200 mg/dl), and HDL cholesterol >1.0 mmol/l (>39 mg/dl).

CV6 Refer early for further investigation and consideration of revascularization those with problematic or symptomatic peripheral arterial disease, those with problems from coronary artery disease, and those with evidence of carotid disease.

## ■ Comprehensive care

$CV_C1$ Assessment will be as for *Standard care*, but with more aggressive investigation of asymptomatic peripheral arterial disease, coronary artery disease, and carotid disease. Lipid profiles may be investigated more extensively to give better direct assessments of LDL cholesterol and apolipoproteins. A specialist lipidologist may be consulted.

$CV_C2$ Interventions will be as for *Standard care* but with aggressive lipid lowering for all, using multiple therapies and more expensive/efficacious statins except where LDL cholesterol, triglycerides and HDL cholesterol are all within target ranges.

$CV_C3$ Antiplatelet agents to consider might include clopidogrel substituted for aspirin, in particular for those with multiple CVD events/problems, peripheral arterial disease, or previous coronary bypass grafting.

$CV_C4$ Renin-angiotensin system blockers are an option for added CV risk protection.

## ■ Minimal care

$CV_M1$ Assessment will be as for *Standard care*, with lipid profile measures if available.

$CV_M2$ Management will be as for *Standard care*, but using statins or fibrates only where these are available at reasonable cost from generics' manufacturers, and in particular for those with known CVD. Statins may be used even if the serum lipid profile cannot be measured.

$CV_M3$ Revascularization procedures will generally not be available, but where possible those limited by symptoms should be so referred.

## Rationale

Cardiovascular disease is the major cause of mortality and morbidity in people with Type 2 diabetes. Indeed some studies have suggested a risk similar to that of people without diabetes but with declared CVD. While others 'merely' show markedly increased risk, some cohorts with particular risk factors have shown extreme risk. Assessment, but more particularly aggressive management, of CV risk factors in Type 2 diabetes is then seen as a core part of care. Some of the risk relates to blood pressure control and blood glucose control and is addressed elsewhere in this guideline, as are the lifestyle interventions which generally benefit the whole spectrum of CV risk factors.

## Evidence-base

The epidemiological evidence that cardiovascular disease is the major cause of mortality in people with Type 2 diabetes is extensive, as is the evidence that the risk is considerably elevated above that of the background population, even where that population is itself prone to high levels of vascular disease. More controversy surrounds the extent of the increased risk. A much quoted paper by Haffner et al. [1] suggested that people with Type 2 diabetes have a CV risk equivalent to non-diabetic people with previous CVD, but this has not in general been supported by other data [2]. The evidence that people with Type 2 diabetes have an abnormal, atherogenic, lipid profile (high triglycerides, low HDL cholesterol, small dense LDL) is generally accepted, and leads all the major guidelines which have addressed the area to recommend assessment of a full serum lipid profile (total cholesterol, HDL cholesterol, LDL cholesterol (derived), triglycerides) as a guide to therapy [3-7].

Since people with Type 2 diabetes may or may not have a high LDL cholesterol (as in the general population), and may have triglyceride/HDL levels anywhere from normal to highly abnormal, decision paths to therapy are uncertain and do vary between evidence-based recommendations. A further problem is assessment of risk. The HPS study (of simvastatin) recruited people with diabetes even if they had no history of cardiovascular risk, and the results showed strong benefit [8]. CARDS similarly studied people with diabetes who had no overt evidence of CVD, and showed marked benefit with atorvastatin [9]. These studies suggest statin treatment for all people with Type 2 diabetes without assessment of risk, if over 40 yr of age. This view is not universally accepted.

The situation is complicated by the difficulty of assessing CV risk in people with diabetes, due to a two-to-three-fold underestimation of risk from tables, charts and engines derived from the Framingham study. This led the NICE group to suggest risk estimation based on a lower threshold than used generally in the UK at that time [6], but the advent of the validated risk engine based on the UKPDS study does now allow CV risk to be appropriately calculated [10]. Nevertheless, since the calculation almost inevitably suggests high risk in people with other risk factors, the universal application of statins in the middle-aged and older groups may be justified. The Canadian guideline states that there is a strong evidence-base for considering nearly everyone with Type 2 diabetes as high risk [5]. However, little evidence is available on people with younger-onset Type 2 diabetes, or their CV risk, although this would seem likely to be high relative to their peers.

Cost-effectiveness of statins is not generally addressed by the evidence-based guidelines, but rather is assumed. Lately simvastatin prices have collapsed in many parts of the world with expiry of patents. This is likely to make them cost-effective in most parts of the world.

The guidelines also address the issue of management of serum triglyceride and HDL cholesterol levels, an area where the evidence-base is softer, but all conclude that management with fibrates is indicated if serum triglyceride levels are raised (triglycerides and HDL cholesterol being inversely correlated). However, there is no easy consensus on the levels at which fibrates should be introduced, or on how they should be introduced in combination with statins. The results of the FIELD trial may help to resolve this in late 2005.

While there are safety concerns with lipid-lowering drugs, and notably even rare life-threatening problems related to muscle necrosis, the drugs are life-saving to a degree many times exceeding the safety risk (with appropriate therapeutic cautions), even when fibrates (except gemfibrozil) are used in combination with statins in people with higher risk.

The evidence-base for other lipid-lowering drugs (extended-acting nicotinic acid, concentrated omega 3 fatty acids, ezetimibe) is weaker – indeed these are barely addressed by published evidence-based guidelines, except the Australian lipid control document [4]. These drugs are also expensive for the degree of lipid-lowering gained and, as noted in the Australian guideline, some may lead to minor deterioration of blood glucose control. It would seem, therefore, that their use should be reserved for uncontrolled hyperlipidaemia on the first-line agents, or intolerance of these.

The use of anti-platelet agents is also addressed by some of the major guidelines (most extensively by the Australian macrovascular prevention guideline and the NICE lipid-lowering guideline [3,6]), with a general recommendation of endorsement for the widespread use of low-dose aspirin, the most specific evidence coming from within the ETDRS and HOT studies [11,12], and the most complete review that of Eccles and colleagues [13]. The Canadian guideline [5] notes a more recent meta-analysis of anti-platelet therapy showing a significant 22±2 % (±SE) reduction in vascular events among all high-risk patients in 195 trials but only a non-significant 7±8 % reduction in people with diabetes (9 trials) [14]. Nevertheless, efficacy is accepted, although the risk of bleeding results in advice in the NICE [6] and SIGN guidelines [7] restricting use to people at calculated risk (which would, however, be most people with

Type 2 diabetes) and with some caution over uncontrolled hypertension. The use of clopidogrel (at least as effective but much more expensive), where considered, is only recommended for people with aspirin intolerance.

Most other aspects of CV risk protection, notably blood glucose and blood pressure control, physical activity, and body weight control, are addressed elsewhere in this and other guidelines. However, there is also an evidence-base for integrated multiple risk factor intervention in particularly high-risk people (with microalbuminuria), showing very powerful absolute and relative risk reductions [15]. Evidence on smoking and CVD is not generally addressed, the advice given simply being in line with general medical practice, based on consideration of evidence for the general population.

## Consideration

Cardiovascular risk protection for people with Type 2 diabetes is an area which is found to be of high need, but with good and often strong evidence of ability to meet that need. One obvious problem is the need to extrapolate evidence in some areas from groups of people who do not have diabetes, for example as regards aspirin therapy. However, because event rates are much higher in people with diabetes (particularly with regard to 'primary' prevention) the gains and cost-effectiveness are also potentially much better, so that the risks of extrapolation of evidence are relatively low. This is especially true because the processes of arterial damage in people with Type 2 diabetes are similar pathologically to those occurring in the general population, though usually present (as in the case of platelet abnormalities) to a more abnormal degree.

Accordingly, the recommendations are for very active management. Statins and aspirin use are given prominence, as best founded in evidence, but the associations of hypertriglyceridaemia and low HDL cholesterol with poor outcomes, together with the limited trial evidence, lead also to strong recommendations over use of fibrates. In these circumstances assessment of risk has a relatively minor role, but is found useful educationally, and clearly can only be done formally using a risk engine properly validated for cohorts of people with diabetes in continuing care.

## Implementation

The recommendations require access to measurement of a full lipid profile and supporting biochemistry, and to aspirin and statins and fibrate drugs as a minimum. Structured annual assessment and record-keeping should be instituted.

## Evaluation

Evaluation is by achieved lipid levels, especially LDL cholesterol and triglycerides, and numbers of people treated (and in particular with elevated levels or existing cardiovascular disease) with statins, fibrates, and aspirin. In general, cardiovascular outcome rates are difficult to assess except in very large populations.

## References


1. Haffner SM, Lehto S, Rönnemaa T, Pyorala K, Laakso M. Mortality from coronary heart disease in subjects with type 2 diabetes and in nondiabetic subjects with and without prior myocardial infarction. N Engl J Med 1998; 339: 229-34.
2. Evans JMM, Wang J, Morris AD. Comparison of cardiovascular risk between patients with type 2 diabetes and those who had had a myocardial infarction: cross sectional and cohort studies. BMJ 2002; 324: 939-42.
3. Newman H, Colagiuri S, Chen M, Colagiuri R. Evidence Based Guidelines for Type 2 Diabetes: Macrovascular disease. Canberra: Diabetes Australia & NHMRC, 2004. http://www.diabetesaustralia.com.au
4. Best J, Colagiuri S, Chen M, Colagiuri R. Evidence Based Guidelines for Type 2 Diabetes: Lipid Control. Canberra: Diabetes Australia & NHMRC, 2004. http://www.diabetesaustralia.com.au
5. Canadian Diabetes Association Clinical Practice Guidelines Expert Committee. Canadian Diabetes Association 2003 Clinical Practice Guidelines for the Prevention and Management of Diabetes in Canada. Canadian Journal of Diabetes 2003; 27(Suppl 2): S58-S65. http://www.diabetes.ca
6. McIntosh A, Hutchinson A, Feder G, Durrington P, Elkeles R, Hitman GA, et al. Clinical guidelines and evidence review for Type 2 diabetes: Lipids Management. Sheffield: ScHARR, University of Sheffield, 2002. http://www.nice.org.uk/pdf/lipidsfull_guideline.pdf
7. Scottish Intercollegiate Guidelines Network. SIGN 55. Management of Diabetes, 2001. http://www.sign.ac.uk
8. Heart Protection Study Collaborative Group. MRC/BHF Heart Protection Study of cholesterol-lowering with simvastatin in 5963 people with diabetes: a randomised placebo-controlled trial. Lancet 2003; 361: 2005-16.
9. Colhoun HM, Betteridge DJ, Durrington PN, Hitman GA, Neil HAW, Livingston SJ, et al., on behalf of the CARDS investigators. Primary prevention of cardiovascular disease with atorvastatin in Type 2 diabetes in the Collaborative Atorvastatin Diabetes Study (CARDS): a multicentre randomized controlled trial. Lancet 2004; 364: 685-96.

10. Stevens R, Kothari V, Adler AI, Stratton IM, Holman RR. UKPDS 56: The UKPDS Risk Engine: a model for the risk of coronary heart disease in type 2 diabetes. Clin Sci 2001; 101: 671-79.
11. ETDRS Investigators. Aspirin effects on mortality and morbidity in patients with diabetes mellitus. Early Treatment Diabetic Retinopathy Study report 14. JAMA 1992; 268: 1292-300.
12. Hansson L, Zanchetti A, Carruthers SG, Dahlof B, Elmfeldt D, Julius S, et al. Effects of intensive blood-pressure lowering and low-dose aspirin in patients with hypertension: principal results of the Hypertension Optimal Treatment (HOT) randomised trial. Lancet 1998; 351: 1755-62.
13. Eccles M, Freemantle N, Mason J. North of England evidence based guidelines development project: Evidence based clinical practice guideline: aspirin for the secondary prophylaxis of vascular disease in primary care. BMJ 1998; 316: 1303-09.
14. Antithrombotic Trialists' Collaboration. Collaborative meta-analysis of randomised trials of antiplatelet therapy for prevention of death, myocardial infarction, and stroke in high risk patients. BMJ 2002; 324: 71-86.
15. Gaede P, Vedel P, Larsen N, Jensen GVH, Parving H-H, Pedersen O. Multifactorial intervention and cardiovascular disease in patients with Type 2 diabetes. N Engl J Med 2003; 348: 383-93.

# Eye screening

13

These guidelines are concerned with preventative *diabetes* care. No advice is given on the further investigation of retinopathy by an ophthalmic specialist, or the subsequent use of laser or other retinal therapy, of vitrectomy, or other tertiary care. It is noted that a substantial evidence-base does exist for these techniques in the prevention of visual loss.

## Recommendations

### ■ Standard care

ES1 Ensure that examination of the eyes of people with Type 2 diabetes is performed around the time of diagnosis and then annually as part of a formal recall process:

- measure and document visual acuity, corrected with glasses or pinhole
- assess retinopathy:
  - using retinal photography through dilated pupils, performed by an appropriately trained health-care professional, or
  - by examination by an ophthalmic specialist.

ES2 Discuss the reasons for eye examination with the person with diabetes.

ES3 Use tropicamide to dilate pupils, unless contra-indicated, after discussing the implications and obtaining agreement of the person with diabetes.

ES4 Classify the findings of eye examination as requiring: routine annual review, earlier review, or referral to an ophthalmologist (if not making the examination).

The following frequency of screening is suggested:

- 12 months if no or minimal unchanged retinopathy
- 3 to 6 months if worsening since last examination
- more often during pregnancy.

ES5 The following situations require specialist referral:

- the same day:
  - sudden loss of vision
  - evidence of retinal detachment
- within 1 week:
  - evidence of pre-retinal and/or vitreous haemorrhage
  - new vessel formation or rubeosis iridis

- within 1-2 months:
  - advanced retinal lesions
  - unexplained deterioration of visual acuity
  - macular oedema
  - unexplained retinal findings
  - cataract
  - inability to visualize fundus.

ES6 Advise that good control of blood glucose, blood pressure, and blood lipids (see relevant sections of this guideline) can help to reduce the risk of eye damage developing or worsening.

ES7 Advise that diabetic retinopathy is not a contra-indication for use of aspirin if this is indicated for prevention of cardiovascular disease.

ES8 Advise that tests of intra-ocular pressure should be made periodically.

## Comprehensive care

$ES_C1$ Retinal screening will be as for *Standard care* in most respects, but could use seven-field stereoscopic colour fundus photography interpreted by a trained reader (where a retinal ophthalmological specialist is not anyway performing the eye check).

## Minimal care

$ES_M1$ Use direct fundoscopy through dilated pupils, performed by a member of the health-care team who is properly trained and has appropriate experience to assess retinopathy.

$ES_M2$ Check visual acuity.

$ES_M3$ Repeat review, referral, and preventative therapy are as for *Standard care*.

## Rationale

Diabetic retinopathy is the most common complication of diabetes and a major cause of visual loss. Damage (maculopathy) to the area of the retina used for fine and central vision (the macular area around the fovea) is the largest problem in people with Type 2 diabetes, though classical retinopathy with new vessels and consequent problems is also important. Measures to control blood glucose and blood pressure (discussed elsewhere) can help to prevent onset and delay worsening of retinopathy, but most people with retinopathy will be asymptomatic until the damage is far advanced. Early detection by regular surveillance is thus essential if people with sight-threatening retinopathy are to be identified in time to offer them the laser treatment which can prevent visual loss.

## Evidence-base

General diabetes guidelines which address the subject of eye screening [1-4] draw on an evidence-base going back to the 1970s, including the findings of the American studies WESDR, DRS and ETDRS which provide the framework for retinal screening and laser treatment [5-7]. The 'gold standard' screening test of seven-standard field stereoscopic colour fundus photography and associated grading scheme were established by these studies.
In recent years technological developments in digital photography have offered expanding opportunities for recording and transmitting images, with potential for automated grading, reviewed in the NICE Type 1 diabetes guideline [8].

The importance of screening people with Type 2 diabetes at diagnosis relates to the finding that between 21 and 39 % of them already have some retinopathy (which may already be sight-threatening) by this time [3]. In the WESDR 1.6 % of people with Type 2 diabetes were legally blind [5]. For people who have no retinopathy at diagnosis of Type 2 diabetes, the chance of developing sight-threatening retinopathy within 2 years is less than 1 % [1]. Although there is some argument as to whether such people need to receive screening as often as annually, and the Canadian guideline recommends every 1 to 2 years [3], the other three favoured annual systematic review [1,2,4] pending further information identifying sub-groups which might safely have longer review periods [2]. Cataract is another important cause of visual loss in people with diabetes, being twice as common as in people without diabetes [1].

Support for optimized glucose control and tighter blood pressure control (see elsewhere) derives from the reduction in risk of microvascular complications found in the UKPDS [9,10]. The effects of aspirin were investigated in the ETDRS (reported in reference 3). High levels of LDL cholesterol were associated with hard exudates in the ETDRS [11].

Recent review of screening methods found that digital photography best met the needs of appropriate sensitivity/selectivity, feasibility and opportunities for quality assurance [8]. SIGN found that direct ophthalmoscopy only rarely achieved 80 % sensitivity even when carried out by properly trained operators [1]. Where cost issues were considered [2], attention was drawn to the dependence of cost-effectiveness on features such as sensitivity and specificity of screening tests, attendance and prevalence.

## Consideration

The core issue is how to provide regular structured review using either ophthalmological expertise or camera technologies. With regard to the latter, use of digital cameras with eyes dilated to reduce the incidence of screen failures is found to be desirable and cost-effective. However, camera technologies cannot detect macular oedema, so visual acuity testing must accompany photography. Where neither camera technologies nor ophthalmologists can be made available, ophthalmoscopy by a trained observer can detect many problems (though with significantly poorer sensitivity) and is thus recommended in these circumstances.

The availability of laser therapy is currently limited in many parts of the world due to cost and lack of trained expertise. It is noted that raising awareness of eye problems by examination and recording of detected problems can both help individual preventative care (blood glucose and blood pressure control) and provide the necessary evidence for establishment of a laser service.

## Implementation

Staff requirements are sufficient numbers of experienced ophthalmologists, optometrists and other health-care professionals to perform the screening, and sufficient ophthalmologists to perform laser therapy, and training of such staff. Equipment for screening and treatment will be required, as will a structured recall system and record. All screening modalities require quality assurance checks; for retinal photography it has been suggested this should happen for around 1 % of photographs [1].

A national or regional advisory group, including representation of ophthalmologists, optometrists, internists and people with diabetes, can work with health funders to define such issues as: criteria for screening and treatment; training and education programmes; provision of accessible facilities; awareness programmes; strategies for programme implementation and guideline dissemination; information systems (for monitoring diabetic eye disease, follow-up and recall, collection of baseline and annual data); annual reports based on defined indicators.

## Evaluation

The percentage of records containing the results of eye examination within a 12-month period is easily evaluated. Where such records are of sight-threatening retinopathy or decrease of visual acuity, evidence of review by (or referral to) an ophthalmological specialist should be present. Eye screening services can be checked for appropriately trained personnel, and facilities sufficient to ensure diabetes population coverage. Evidence of quality checks should be assessed. Evidence of control of rates of visual loss is more difficult to gather unless the records of ophthalmological services can be linked to those of diabetes services.

## References

1. Scottish Intercollegiate Guidelines Network. SIGN 55. Management of Diabetes, 2001. http://www.sign.ac.uk
2. Hutchinson A, McIntosh A, Peters J, Home P, Feder G, Baker R, et al. Clinical guidelines and evidence review for Type 2 diabetes: Diabetic retinopathy: early management and screening. Sheffield: ScHARR, University of Sheffield, 2001. http://www.nice.org.uk/pdf/diabetesretinopathyfullreport.pdf

3. Canadian Diabetes Association Clinical Practice Guidelines Expert Committee. Canadian Diabetes Association 2003 Clinical Practice Guidelines for the Prevention and Management of Diabetes in Canada. Canadian Journal of Diabetes 2003; 27(Suppl 2): S76-S80. http://www.diabetes.ca
4. Hammes HP, Bertram B, Bornfeld N, Gandjour A, Parandeh-Shab F, Danne D, et al. Diagnostik, Therapie und Verlaufskontrolle der diabetischen Retinopathie und Makulopathie. In: Scherbaum WA, Lauterbach KW, Renner R (eds) Evidenzbasierte Diabetes-Leitlinien DDG, 1st edn. Deutsche Diabetes Gesellschaft 2000. http://www.deutsche-diabetes-gesellschaft.de (updated 2004)
5. Klein R, Klein BEK, Moss SE, Davis MD, DeMets DL. The Wisconsin epidemiologic study of diabetic retinopathy III. Prevalence and risk of diabetic retinopathy when age at diagnosis is 30 or more years. Arch Ophthalmol 1984; 182: 527-32.
6. Diabetic Retinopathy Study Research Group. Photocoagulation treatment of proliferative diabetic retinopathy. Clinical application of DRS findings. DRS Report No. 8. Ophthalmology 1981; 88: 583-600.
7. Early Treatment Diabetic Retinopathy Study Research Group. Early photocoagulation for diabetic retinopathy. ETDRS report number 9. Ophthalmology 1991; 98: 741-56.
8. The National Collaborating Centre for Chronic Conditions. Type 1 Diabetes in Adults. National clinical guideline for diagnosis and management in primary and secondary care. http://www.rcplondon.ac.uk/pubs/books/DIA/index.asp
9. UK Prospective Diabetes Study (UKPDS) Group. Intensive blood-glucose control with sulphonylureas or insulin compared with conventional treatment and risk of complications in patients with type 2 diabetes (UKPDS 33). Lancet 1998; 352: 837-53.
10. UK Prospective Diabetes Study Group. Tight blood pressure control and risk of macrovascular and microvascular complications in type 2 diabetes: UKPDS 38. BMJ 1998; 317: 703-13.
11. Chew EY, Klein ML, Ferris 3rd FL, Remaley NA, Murphy RP, Chantry K, et al. Association of elevated serum lipid levels with retinal hard exudate in diabetic retinopathy. Early Treatment Diabetic Retinopathy Study (ETDRS) Report 22. Arch Ophthalmol 1996; 114: 1079-84.

# Kidney damage

14

These guidelines are concerned with preventative *diabetes* care. No advice is given on further investigation of kidney disease by a renal specialist, or subsequent tertiary care.

## Recommendations

### Standard care

KD1 Check annually for proteinuria in an early morning urine sample (or a random sample otherwise) using a dipstick.

- if dipstick test positive,
  - check for urinary tract infection
  - obtain a laboratory urine protein:creatinine ratio (PCR)
- if dipstick test negative, check urine albumin using:
  - laboratory or site-of-care urine albumin:creatinine ratio (ACR), or
  - a semi-quantitative reagent strip if ACR test is unavailable.

Measure serum creatinine annually, and calculate GFR ('eGFR').

KD2 If PCR or ACR is raised (microalbuminuria ACR >2.5 mg/mmol in men, >3.5 mg/mmol in women; or 30 mg/g), repeat twice over the following 4 months.

- confirm as positive if proteinuria or raised urine albumin on two of three occasions
- if both repeat tests are not raised, check again annually.

KD3 Manage those with raised urine albumin or proteinuria or reduced eGFR (<90 ml/min/1.73 $m^2$ and falling) as follows:

- use ACE-inhibitor or A2RB titrated to maximum tolerated dose
- intensify management of blood pressure (actively target <130/80 mmHg) using drugs and dietary modification (low salt intake)
- intensify management of blood glucose (target DCCT-aligned $HbA_{1c}$ <6.5 %)
- monitor progression by ACR or PCR, serum creatinine and potassium; calculate eGFR; discuss results
- advise limiting protein intake to 0.8 g/kg daily if proteinuric
- intensify other renal and cardiovascular protection measures (not smoking, aspirin therapy, lipid-lowering therapy).

KD4 Measure Hb/ferritin every 6 months if eGFR <90 ml/min/1.73 $m^2$, give iron or other haematinics if indicated, and refer to nephrologist if still anaemic despite supplements (Hb <11 g/dl in pre-menopausal women, <12 g/dl in others).

KD5 Refer to a nephrologist when eGFR <60 ml/min/1.73 $m^2$, or earlier if symptomatic or biochemical or fluid retention problems occur.

## ■ Comprehensive care

$KD_C1$ This is in general as for *Standard care*, but assessment of albuminuria would always be by a laboratory quantitative method (ACR).

$KD_C2$ Investigations to exclude other possible causes of renal disease for all with raised ACR or PCR might include auto-antibodies, ultrasound, biopsy.

## ■ Minimal care

$KD_M1$ Check annually for proteinuria in an early morning urine sample (or a random sample otherwise) using dipstick or sulfosalicylic acid method.

- if test positive,
  - exclude urinary tract infection by microscopy (and culture if possible)
  - if possible, obtain a laboratory protein:creatinine ratio (PCR) and repeat on two occasions over the following 6 months (proteinuria confirmed if positive on two of three occasions)
- if test negative, check again annually.

If available measure serum creatinine (or urea) annually.

$KD_M2$ Manage those with proteinuria as follows:

- advise to avoid risk factors (analgesic use, alcohol consumption, illicit drug use), to limit protein intake (to 0.8 g/kg daily), and not to smoke
- aim for blood pressure <130/80 mmHg using any anti-hypertensive drug and control of salt intake
- consider use of ACE-inhibitors if available
- aim to achieve targets for blood glucose control
- aim to improve lipid profile using available drugs
- check proteinuric status/progression annually
- measure serum creatinine or urea every 6 months.

## Rationale

Diabetic renal disease has only received less attention in people with Type 2 diabetes in the past because their life expectancy was limited by cardiovascular disease. However, because of the higher incidence of Type 2 than Type 1 diabetes, renal failure in the former group has always been a significant cause of morbidity and mortality. With increasing numbers of people with Type 2 diabetes, younger age of onset, and better cardiovascular protection measures, the health impact of renal impairment in this population and in individuals is growing. While the major effort of management must go to primary prevention (good blood glucose and blood pressure control from early diagnosis), the success of interventions at a later stage (see below) suggests that detection of developing kidney damage would be useful.

## Evidence-base

The evidence-based diabetes guidelines which address the subject of nephropathy describe the early stages of kidney damage in terms of albumin excretion rate (AER) increasing through 'microalbuminuria' to 'macroalbuminuria' (at which point it equates with proteinuria, 'overt nephropathy') [1-6]. There is general agreement on annual screening, and on the albumin:creatinine ratio (which corrects for urine concentration) as the preferred method of detection, but cut-off values differ somewhat, microalbuminuria being defined as 30 mg/g in the USA [1], 2.0/2.8 mg/mmol (men/women) in Canada [2], and 2.5/3.5 mg/mmol in Europe [3-6], and macroalbuminuria as 300 mg/g, 20/28 mg/mmol, and 30 mg/mmol respectively. Issues surrounding screening tests are reviewed in detail by the NICE Type 2 guideline [4],

with attention drawn to the day-to-day variation in albumin excretion which underlines the need for confirmatory testing. Monitoring of changes in glomerular filtration rate (which are not necessarily in line with changes in albumin excretion) is emphasized in all the guidelines, which recommend serum creatinine measurement, and more recently emphasize the need for calculation of estimated GFR [1,2].

UKPDS provided clear evidence for the benefits of blood glucose control and blood pressure control in delaying the development of kidney disease [7,8]. Other evidence for the importance of blood pressure control in prevention comes from trials of various anti-hypertensive drugs, and evidence continues to emerge in this area (although there will be no more placebo-controlled trials). Choice of agent stems from evidence on the additional benefits of agents which target the renin-angiotensin system in offering renal and cardiovascular (see *Cardiovascular risk protection*) protection, over and above the blood pressure-lowering effect. Both ACE-inhibitors and the newer A2RBs delay progression from micro- to macro-albuminuria in people with Type 2 diabetes and hypertension [1,2,9]. A2RBs have been shown to delay progression of nephropathy in those who have macroalbuminuria and renal insufficiency (serum creatinine >1.5 mg/dl (>130 μmol/l)) [1]. Of the other anti-hypertensive agents which might be used, the ADA cites evidence that dihydropyridine CCBs do not slow progression of nephropathy so should not be used as first-line therapy in nephropathy [1].

Targets for blood pressure have been tightening in diabetes care generally and the advice to treat to tighter targets for those with albuminuria, 130/75 mmHg as against 140/80 mmHg in people with Type 2 diabetes [4], is perhaps now a minority view, with general advice converging towards 130/80 mmHg for all irrespective of AER [1,2,5]. NICE found that reduction of blood pressure to less than 135/75 mmHg reduced the rate of progression of renal disease, with lowest achieved mean blood pressure being 134/75 mmHg in studies showing benefit in people with Type 2 diabetes and albuminuria [4].

The recommendation on treatment of anaemia once GFR starts to decline is supported by the finding in the RENAAL study that mild anaemia is associated with risk of renal disease progression [10].

Cardiovascular risk is increased in people with microalbuminuria, and further increased in those with proteinuria and/or reduced GFR. The issue of cardiovascular risk is addressed elsewhere in this guideline (see *Cardiovascular risk protection*).

## Consideration

Although it is possible to treat kidney failure by dialysis or transplantation, availability of these very expensive treatments is severely limited in a global context. This makes efforts at prevention all the more important. It has been estimated that, once a dipstick test is positive, time to kidney failure is about 9 years, but that this time-interval can be doubled through appropriate treatment of blood pressure. The issue of targets can be a particular problem in people with Type 2 diabetes who are often more elderly, and in whom attainment of 140/80 mmHg or less can seem impossible even with multiple drugs and reasonable lifestyle intervention. Nevertheless control around this level has been achieved in a number of studies, implying that around half the population can get to (and thus benefit from) lower levels.

## Implementation

Management of blood pressure overlaps with the advice given in *Blood pressure control*. Recurrent measurement and drug dose titration need good access for people with evidence of renal damage, where repeated measurements of potassium and creatinine are particularly important. Additionally the current section requires access to laboratory microalbumin estimation (or availability of semi-quantitative reagent strips), and availability of multiple blood-pressure-lowering drugs and in particular renin-angiotensin system blockers.

## Evaluation

The percentage of people with appropriate urine albumin and serum creatinine measurements should be ascertained. Where abnormalities are detected, evidence of action to ensure tight blood pressure control is required, together with achieved blood pressure. Level of eGFR at which referral to nephrologists occurred may also be determined.

## References


1. American Diabetes Association Position Statement. Standards of Medical Care in Diabetes. Diabetes Care 2005; 28 (Suppl 1): S4-S36.
2. Canadian Diabetes Association Clinical Practice Guidelines Expert Committee. Canadian Diabetes Association 2003 Clinical Practice Guidelines for the Prevention and Management of Diabetes in Canada. Canadian Journal of Diabetes 2003; 27(Suppl 2): S66-S71. http://www.diabetes.ca
3. Scottish Intercollegiate Guidelines Network. SIGN 55.

Management of Diabetes, 2001. http://www.sign.ac.uk
4. McIntosh A, Hutchinson A, Marshall S, Barnes D, Brown V, Hopper S, et al. Clinical Guidelines and Evidence Review for Type 2 Diabetes. Renal Disease: Prevention and Early Management. Sheffield: ScHARR, University of Sheffield, 2002. http://www.nice.org.uk
5. Hasslacher C, Gandjour A, Redaelli M, Bretzel RG, Danne D, Ritz E, et al. Diagnose, Therapie und Verlaufskontrolle der Diabetischen Nephropathie. In: Scherbaum WA, Lauterbach KW, Renner R (eds) Evidenzbasierte Diabetes-Leitlinien DDG, 1st edn. Deutsche Diabetes Gesellschaft 2000. http://www.deutsche-diabetes-gesellschaft.de (updated 2002)
6. The National Collaborating Centre for Chronic Conditions. Type 1 Diabetes in Adults. National clinical guideline for diagnosis and management in primary and secondary care. http://www.rcplondon.ac.uk/pubs/books/DIA/index.asp
7. UK Prospective Diabetes Study (UKPDS) Group. Intensive blood-glucose control with sulphonylureas or insulin compared with conventional treatment and risk of complications in patients with type 2 diabetes (UKPDS 33). Lancet 1998; 352: 837-53.
8. UK Prospective Diabetes Study Group. Tight blood pressure control and risk of macrovascular and microvascular complications in type 2 diabetes: UKPDS 38. BMJ 1998; 317: 703-13.
9. Barnett AH, Bain SC, Bouter P, Karlberg B, Madsbad S, Jervell J, et al. Angiotensin-receptor blockade versus converting-enzyme inhibition in type 2 diabetes and nephropathy. N Engl J Med 2004; 351: 1952-57.
10. Mohanram A, Zhang Z, Shahinfar S, Keane WF, Brenner BM, Toto RD. Anemia and end-stage renal disease in patients with type 2 diabetes and nephropathy. Kidney Int 2004; 66: 1131-38.

# Foot care

15

## Recommendations

### ■ Standard care

FT1 Assess feet of people with diabetes as part of an annual review:

1. history of previous foot ulceration or amputation, symptoms of peripheral arterial disease, physical or visual difficulty in self-foot-care
2. foot deformity (hammer or clawed toes, bone prominences) and footwear; visual evidence of neuropathy (dry skin, callus, dilated veins) or incipient ischaemia; nail deformity or damage
3. detection of neuropathy by 10-g monofilament (or 128-Hz tuning fork); a biothesiometer is an option for quantitative assessment (cut-off point for ulcer risk >25 volts); non-traumatic pin-prick
4. palpation of foot pulses (dorsalis pedis and posterior tibial) and capillary return time; Doppler ankle:brachial pressure ratio (<0.9 for occlusive vascular disease) may be used where pulses are diminished to quantify the abnormality.

FT2 Discuss the reasons for foot review with each person with diabetes as part of the foot-care educational process.

FT3 Agree a foot-care plan based on the findings of annual foot review with each person with diabetes.

Assess and provide necessary foot-care education according to individual need and risks of ulcer and amputation.

FT4 Classify according to findings:

***No added risk***: if no loss of sensation, no signs of peripheral arterial disease, and no other risk factor.

***At risk***: if neuropathy or other single risk factor.

***High risk***:

- diminished sensation plus foot deformities or evidence of peripheral arterial disease
- previous ulceration or amputation (very high risk).

***Foot ulceration or infection***: foot ulcer present.

FT5 Manage according to classification level:

***No added risk***: agree a management plan including foot-care education with each person.

***At risk***: arrange regular review, approximately 6-monthly, by foot-care team. At each review:
1. inspect both feet – ensure provision of local management as indicated
2. evaluate footwear – provide appropriate advice
3. enhance foot-care education.

***High risk***: arrange frequent review every 3-6 months by foot-care team. At each review:
1. inspect both feet – ensure provision of local management as indicated
2. evaluate footwear – provide advice and specialist insoles and shoes if indicated
3. consider need for vascular assessment or referral
4. evaluate and ensure the appropriate provision of intensified foot-care education.

***Foot ulceration or infection*** (including foot-care emergencies): refer to multidisciplinary foot-care team within 24 hours for:
1. appropriate wound management, dressings and debridement as indicated
2. consideration of systemic antibiotic therapy (often longer term) for cellulitis or bone infection as indicated; generic penicillins, macrolides, clindamycin, and/or metronidazole as indicated as first-line, with ciprofloxacin or co-amoxiclav as examples of second-line drugs
3. optimal pressure distribution (casting if indicated and not contra-indicated), investigation and treatment (referral) for vascular insufficiency
4. probing to bone, radiology and scans, MRI imaging, and biopsy where indicated for suspected osteomyelitis
5. optimal blood glucose control
6. specialist footwear and orthotic care (e.g. insoles), and individualized discussion of prevention of recurrence, when ulcer has healed.

FT6 Do not amputate unless:
1. a detailed vascular evaluation has been performed by the vascular staff
2. ischaemic rest pain cannot be managed by analgesia or revascularization
3. a life-threatening foot infection cannot be treated by other measures
4. a non-healing ulcer is accompanied by a higher burden of disease than would result from amputation.

A specialist foot-care team will include doctors with a special interest in diabetes foot care, people with educational skills, and people with formal training in foot care (usually podiatrists or trained nurses).

## Comprehensive care

$FT_C1$ In general this will be as *Standard care*, but the multidisciplinary foot-care team can be enhanced by on-site inclusion of vascular surgeons, orthopaedic surgeons, orthotists, social workers, and psychologists.

$FT_C2$ Foot pressure distribution measurements might be made. Sophisticated vascular scanning and angiography could be available to the foot-care team.

## Minimal care

$FT_M1$ Sensory assessment would be by 10-g monofilament or tuning fork, with or without non-traumatic disposable pin-prick only.

$FT_M2$ Antibiotic therapy would be with generic penicillins, macrolides, and/or metronidazole, intravenously for deep tissue infections, and adjusted by response or culture results.

$FT_M3$ Vascular assessment would be by peripheral pulses and capillary return times only.

$FT_M4$ Vascular referral would be according to findings and local revascularization facilities.

## Rationale

Foot ulceration and limb amputation are among the major drivers of impaired health and of health-care costs in diabetes care. While primary prevention of the underlying damage to nerves and vessels is addressed elsewhere in this guideline, secondary intervention in those developing such risk factors can reduce this burden and cost on both the person with diabetes and society.

## Evidence-base

Because of the potential for improvement of health and reduction of health-care costs, the evidence surrounding diabetes foot-care has been extensively and formally reviewed many times in recent years [1-10].

The output from these documents is very consistent in suggesting that formal regular review to detect people at risk, more regular review of those found to be at risk, and intensive management of those developing foot ulceration and infection can produce major returns in avoiding the health and monetary costs of amputation. Providing foot-care education for all patients, with increased intensity for those at higher risk [11], and vascular interventions where critical ischaemia is identified (or is contributing to ulceration), are also common recommendations arising from the evidence-base.

## Consideration

There is little controversy over the system and needs of diabetes foot-care provision. Most of the recommendations of formal evidence-based guidelines can be implemented with little modification in situations where minimal health-care funding resources are available, as simply removing shoes and examining feet can usefully save people from becoming disabled and unproductive members of their communities.

## Implementation

Appropriate protocols, structured records, and recall systems need to be supported by appropriate training for professionals providing screening and management services. In particular the training and provision of non-medically qualified foot-care assistants (podiatrists or people fulfilling that role) need to be assured. Liaison needs to be established with orthotists and footwear suppliers, and cast

technicians. Facilities for vascular scanning and vascular interventions will be by agreement with vascular surgical staff. Policymakers should be approached to consider the socio-economic burden of diabetes foot problems and assure structural and financial support for preventative strategies.

## Evaluation

Evaluation is by annual incidence of foot ulceration, foot hospitalization, foot ulceration healing rates within defined time-periods, and amputation rates at different levels of the limb.

## References


1. Scottish Intercollegiate Guidelines Network. SIGN 55. Management of Diabetes, 2001. http://www.sign.ac.uk
2. Morbach S, Müller E, Reike H, Risse A, Spraul M. Diagnostik, Therapie, Verlaufskontrolle und Prävention des diabetischen Fußsyndroms. In: Scherbaum WA, Kiess W, Landgraf R (eds) Evidenzbasierte Diabetes-Leitlinien DDG. Diabetes und Stoffwechsel 2004; 13 (Suppl 2). http://www.deutsche-diabetes-gesellschaft.de
3. Canadian Diabetes Association Clinical Practice Guidelines Expert Committee. Canadian Diabetes Association 2003 Clinical Practice Guidelines for the Prevention and Management of Diabetes in Canada. Canadian Journal of Diabetes 2003; 27(Suppl 2): S74-S75. http://www.diabetes.ca
4. National Institute for Clinical Excellence. Type 2 diabetes – footcare. London: National Institute for Clinical Excellence, 2004. http://www.nice.org.uk/page.aspx?o=101518
5. Institute for Clinical Systems Improvement (Bloomington, MN, USA). Management of Type 2 Diabetes Mellitus, 2004. http://www.icsi.org/knowledge
6. Campbell L, Colagiuri S, O'Rourke S, Chen M, Colagiuri R. Evidence Based Guidelines for Type 2 Diabetes. Detection and Prevention of Foot Problems. Canberra: Diabetes Australia & NHMRC, 2005. http://www.diabetesaustralia.com.au
7. International Working Group on the Diabetic Foot. Apelqvist J, Bakker K, Van Houtum WH, Nabuurs-Franssen MH, Shaper NC (eds) International Consensus on the Diabetic Foot. Maastricht, The Netherlands, 1999.
8. Lipsky BA. A report from the international consensus on diagnosing and treating the infected diabetic foot. Diabetes Metab Res Rev 2004; 20 (Suppl 1): S68-S77.
9. Eldor R, Raz I, Ben Yehuda A, Boulton AJM. New and experimental approaches to treatment of diabetic foot ulcers: a comprehensive review of emerging treatment strategies. Diabet Med 2004; 21: 1161-73.
10. Singh N, Armstrong DG, Lipsky BA. Preventing foot ulcers in patients with diabetes. JAMA 2005; 293: 217-28.
11. Valk GD, Kriegsman DMW, Assendelft WJJ. Patient education for preventing diabetic foot ulceration. A systematic review. Endocrinol Metab Clin North Am 2002; 31: 633-58.

# Nerve damage

16

## Recommendations

### ■ Standard care

NU1 Diagnose sensorimotor nerve damage by history and examination (monofilament with or without temperature, non-traumatic pin-prick, vibration (tuning fork), ankle reflexes), and/or simple quantitative testing (e.g. vibration perception).

Use serum $B_{12}$, thyroid function tests, creatinine/urea, and drug history to exclude other causes.

NU2 Diagnose symptomatic (painful) diabetic neuropathy by excluding other possible causes of the symptoms.

Manage by stabilizing blood glucose control, and treatment with tricyclic drugs if simple analgesia is not successful.

Further treatment options include pregabalin/gabapentin and valproate, then tramadol, duloxetine, and oxycodone. Further management normally requires referral to a pain control team.

Be aware of the psychological impact of continuing symptoms, particularly if sleep is disturbed.

NU3 Diagnose erectile dysfunction by history (including drug history), exclusion of endocrine conditions (measure prolactin and testosterone), and a trial of a PDE5 inhibitor (where not contra-indicated by nitrate therapy).

Consider other approaches such as intra-urethral or intracavernosal drugs and sexual and relationship counselling, where PDE5 inhibitors fail or cannot be used.

NU4 Diagnose gastroparesis by history, trial of a prokinetic drug (metoclopramide, domperidone), and if troublesome by gastric emptying studies.

NU5 Diagnose cardiovascular autonomic neuropathy by resting heart rate and heart rate response to provocation tests (lying-standing, Valsalva, deep breathing), and by lying and standing blood pressure.

Advise anaesthetists when relevant where this is present.

## Comprehensive care

$NU_C1$ This would be as for *Standard care*, but screening and diagnostic testing could also include a programme of quantitative sensory testing (vibration and temperature), electrophysiology, and autonomic function tests.

## Minimal care

$NU_M1$ Screen and diagnose sensorimotor nerve damage by history of symptoms, and sensory assessment by 10-g monofilament or tuning fork with/without non-traumatic disposable pin-prick (as *Foot care*), and ankle reflexes.

$NU_M2$ Manage symptomatic (painful) diabetic neuropathy by excluding other causes, stabilizing glycaemic control, and treatment with tricyclic drugs if simple analgesia is not successful. Opiate analgesia may be necessary as locally available.

$NU_M3$ Assess erectile dysfunction by history and examination, to consider possible contributions of other medication or disease.

## Rationale

Neuropathy (nerve damage) is a common late complication of Type 2 diabetes. It contributes not only to foot problems (see *Foot care*) but also to a range of troublesome symptoms including pain/paraesthesiae and (where the autonomic nervous system is involved) gastro-intestinal, bladder and sexual problems. New therapeutic options have emerged in recent years.

## Evidence-base

Aspects of neuropathy which do not relate directly to foot care have received less attention in evidence-based guidelines [1-4], and some divergence in recommendations can be accounted for by recently emerging evidence on treatment options for painful neuropathy [5,6]. There is general agreement that stabilizing glycaemic control is important in the medium and longer term, and that tricyclic drugs should be used as first-line therapy for painful neuropathy, although side-effects are common.

Exclusion of non-diabetic causes of neuropathy is important because these may account for 10 % of cases of neuropathy in people with diabetes [7]. The range of tests available in clinical and research settings is detailed in two technical reviews [8,9].

Erectile dysfunction is addressed by three of the guidelines, which draw on evidence from Type 1 as well as Type 2 diabetes [1-3]. They conclude that the condition is rarely of simple causation, that it is important to consider the possible contribution of other medications and medical conditions, but that the expensive PDE5 inhibitors are worth a trial.

The evidence-base on some of the rarer aspects of autonomic neuropathy is weak, including that for gastroparesis, and cardiovascular parasympathetic autonomic neuropathy. In general, other guidelines have relied on conventional wisdom in making recommendations over the management of gastroparesis, orthostatic hypotension, bladder dysfunction, and nocturnal diarrhoea.

## Consideration

The costs of newer therapies were felt to argue against their use in situations where resources could be better directed to prevention by measures aimed at improving and stabilizing glycaemic control. A limited number of tests were felt to be appropriate in the clinical setting, but the practice generally recommended in this area simply follows established medical lines.

## Implementation

Appropriate protocols should be developed for sensory testing. Recommended drugs should be available according to level of resources. Medical teams need to remain trained in the diverse manifestations of autonomic neuropathy.

## Evaluation

Evidence should be available of records of regular surveillance for neuropathic symptoms, usually as part of direct questioning in programmed annual review. Where appropriate, record should also be available of direct questioning for erectile dysfunction. The availability of simple equipment for surveillance, and of drug supplies, can be evaluated.

## References


1. Canadian Diabetes Association Clinical Practice Guidelines Expert Committee. Canadian Diabetes Association 2003 Clinical Practice Guidelines for the Prevention and Management of Diabetes in Canada. Canadian Journal of Diabetes 2003; 27(Suppl 2): S72-S73, S81-S82. http://www.diabetes.ca
2. The National Collaborating Centre for Chronic Conditions. Type 1 Diabetes in Adults. National clinical guideline for diagnosis and management in primary and secondary care. http://www.rcplondon.ac.uk/pubs/books/DIA/index.asp
3. Haslbeck M, Luft D, Neundörfer B, Redaelli M, Stracke H, Ziegler D, et al. Diagnose, Therapie und Verlaufskontrolle der diabetischen Neuropathie. In: Scherbaum WA, Landgraf R (eds) Evidenzbasierte Diabetes-Leitlinien DDG, 2nd edn. Deutsche Diabetes-Gesellschaft 2004. http://www.deutsche-diabetes-gesellschaft.de
4. Scottish Intercollegiate Guidelines Network. SIGN 55. Management of Diabetes, 2001. http://www.sign.ac.uk
5. Boulton AJM, Vinik AI, Arezzo JC, Bril V, Feldman EL, Freeman R, et al. Diabetic neuropathies: a statement by the American Diabetes Association. Diabetes Care 2005; 28: 956-62.
6. Backonja M, Glanzman RL. Gabapentin dosing for neuropathic pain: evidence from randomized placebo-controlled clinical trials. Clin Ther 2003; 25: 81-104.
7. Dyck PJ, Kratz KM, Karnes JL, Litchy WJ, Klein R, Pach JM, et al. The prevalence by staged severity of various types of diabetic neuropathy, retinopathy, and nephropathy in a population-based cohort: The Rochester Diabetic Neuropathy Study. Neurology 1993: 43: 817-24.
8. Boulton AJM, Malik RA, Arezzo JC, Sosenko JM. Diabetic somatic neuropathies (Technical Review). Diabetes Care 2004; 27: 1458-86.
9. Vinik AI, Maser RE, Mitchell B, Freeman R. Diabetic autonomic neuropathy: a technical review. Diabetes Care 2003; 26: 1553-79.

# Pregnancy

17

Whenever pregnancy is complicated by diabetes, close liaison between health-care professionals involved in diabetes, obstetric and neonatal care will help to achieve the desired outcome of a healthy mother and baby.
This guideline only addresses areas of pregnancy care commonly affected by the co-existence of diabetes, and not routine obstetric care such as fetal scanning and monitoring.

## Recommendations

### Standard care

*Pre-pregnancy counselling*

PR1 Identify possibility of pregnancy annually by direct questioning in all fertile women of child-bearing age with diabetes. Provide contraceptive advice where appropriate.

PR2 Offer pre-pregnancy advice to all women so identified, including as appropriate:

- education on the management of pregnancy with diabetes
- optimization of blood glucose control (pre-conception target DCCT-aligned $HbA_{1c}$ <6.1 %)
- stopping oral glucose-lowering drugs (metformin may still be indicated), and starting insulin where appropriate
- optimization of blood pressure control (to <130/80 mmHg)
- stopping ACE-inhibitors and A2RBs (use methyldopa, nifedipine MR, labetalol)
- stopping statins and fibrates
- assessment of eye and kidney damage (see *Eye screening, Kidney damage*); discuss and manage identified problems
- assessment of thyroid function
- advice on alcohol and smoking
- folic acid therapy.

*Screening for undiagnosed or new (gestational) diabetes in pregnancy*

PR3 In women at high risk of diabetes (previous gestational diabetes, obesity – especially abdominal obesity, population with high prevalence of diabetes) provide healthy lifestyle advice (nutrition and physical activity) from first pre-natal visit; check for hyperglycaemia at first pre-natal visit; perform 75-g OGTT [1] if indicated.

PR4 In all women, measure plasma glucose at first visit after week 20 (24-28 weeks in low risk women); perform 75-g OGTT if abnormal.

PR5 Manage as diabetes if fasting plasma glucose ≥7.0 mmol/l (>125 mg/dl) and/or 2-h plasma glucose ≥7.8 mmol/l (≥140 mg/dl).

### *Management during pregnancy*

PR6 Review understanding of management of diabetes in pregnancy, current drug therapy (see *PR2*), blood glucose control, diabetes complications, and presence of other medical conditions. Advise as appropriate.

PR7 Examine eyes at first pre-natal visit and each trimester.

PR8 Offer medical nutrition therapy and education. If overweight, advise a diet suitable for someone of optimal weight. Encourage moderate exercise such as walking.

PR9 Review frequently, depending on achievement of blood glucose control targets, and management of other diabetes-associated and obstetric problems.

PR10 Aim for DCCT-aligned $HbA_{1c}$ <6.0 %, or lower if safely achievable, using self-monitoring of blood glucose to 3.3-6.7 mmol/l (60-120 mg/dl), four times daily (pre-breakfast and 1-2 h after each meal), and insulin therapy if indicated.

PR11 Manage insulin therapy through careful and intensive self-monitoring and dose adjustment, expecting a rise in insulin requirements as pregnancy proceeds. Insulin requirements may be further disturbed by hyperemesis or use of steroid therapy, and in-patient care may be needed.

PR12 Monitor weight gain and blood pressure and advise/treat accordingly. Blood pressure should be <130/80 mmHg, avoiding the use of renin-angiotensin system blocking drugs.

### *Labour and delivery*

PR13 Use intravenous insulin (if on insulin or if needed) during labour.

PR14 Anticipate changed insulin requirements, and thus need for more frequent glucose monitoring, if continuing insulin postpartum and during lactation.

PR15 Provide appropriate care and facilities for the newborn.

PR16 At 45 to 60 days after pregnancy, check for diabetes in women who had developed new diabetes in pregnancy. If then non-diabetic, advise on the high risk of future diabetes, and preventative lifestyle measures. Advise check for diabetes annually.

## Comprehensive care

$PR_C1$ This would be as *Standard care* for screening, except that screening for new diabetes after week 20 might go direct to OGTT in situations with high prevalence and where health facilities are available.

$PR_C2$ Specialist ophthalmological review can be offered throughout pregnancy.

$PR_C3$ Personal dietetic support and fitness training can be offered throughout pregnancy.

$PR_C4$ Self-monitoring of capillary blood glucose during pregnancy would be performed more frequently, at times of likely peak and trough plasma glucose concentrations. Continuous glucose monitoring would be a further possibility.

$PR_C5$ $HbA_{1c}$ will be performed at each clinical contact.

$PR_C6$ Insulin delivery might be optimized by the use of continuous subcutaneous insulin infusion.

## Minimal care

$PR_M1$ Most of the procedures under *Standard care* can be offered by a specially trained health-care worker.

$PR_M2$ If laboratory glucose testing is not easily available, capillary blood glucose measurement for fasting and 2-h OGTT estimation can be substituted, using a trained operator and a regularly validated meter system.

$PR_M3$ Where resources allow only very limited access to self-monitoring of blood glucose, use in pregnant women should be a priority.

$PR_M4$ If insulin availability is problematic, consider oral glucose-lowering drugs (not PPAR-$\gamma$ agonists), with the proviso that safety in pregnancy is not fully established.

## Rationale

With increasing numbers of women around the world developing Type 2 diabetes, and doing so at a younger age, and with women in many cultures tending to delay starting a family, the issue of diabetes complicating pregnancy has become increasingly important. These guidelines do not address prevention of Type 2 diabetes, so the increased risk of later development of diabetes in those who experience gestational diabetes (GDM) is not our principal concern here. We focus rather on the care of women with new diabetes in pregnancy, as well as the care of those who already have Type 2 diabetes. Although management of diabetes in pregnancy has been improving, women and their infants remain at higher risk for a number of complications compared with non-diabetic pregnancy. The frequency of congenital anomalies is still high among infants of women with diabetes.

## Evidence-base

The evidence-base for much diabetes pregnancy management is poor, and relies on some cohort studies, an occasional RCT, some retrospective analysis, and considerable clinical experience. Much of the data pertaining to Type 2 diabetes derives from people with Type 1 diabetes or studies of mixed populations. The only guideline formally addressing the area (Type 2 diabetes) is the Canadian guideline (in which most of the recommendations are consensus) [2], though consensus guidelines based on non-formal evidence review were also

prepared by IDF (Europe) [3]. The SIGN guideline [4] includes pregnancy, focusing on Type 1 diabetes, while ADA standards of care include pre-conception care and screening for diabetes in pregnancy [5].

Screening for GDM (defined as glucose intolerance of variable severity with onset or first recognition during pregnancy – which will include undiagnosed Type 2 diabetes) is a controversial issue (and the ongoing HAPO study may help here) [2]. Whether or not, and whom, to screen is likely to depend on prevalence of Type 2 diabetes in women of child-bearing age in the population under consideration; it is difficult therefore to develop universally appropriate recommendations. GDM is an asymptomatic condition most of the time, and there has been no RCT to test the effectiveness of its detection. Diagnostic and management levels remain uncertain. Cohort studies have shown increased risk of adverse outcomes according to levels of plasma glucose, independently of age, obesity and other risk factors. RCTs have shown that treatment of hyperglycaemia in pregnancy reduces macrosomia. The Canadian guideline recommends all pregnant women be screened for GDM between weeks 24 and 28 [2], while different strategies are outlined in other guidelines which address this [4,5,6]. A recent paper has supported the utility of detecting and managing GDM [7].

The guidelines present a confusing picture as regards screening tests for GDM. The use of a 4.7 mmol/l (85 mg/dl) cut-off for fasting plasma glucose is suggested from two analyses [8,9], but other studies have suggested higher cut-offs. Fasting glucose may not be the most appropriate measure, however, and the 75-g OGTT (fasting and 2-h values) advocated by WHO [1] is increasingly used internationally, as noted in a Brazilian study [10].

The Canadian and IDF (Europe) guidelines note the importance of blood glucose control in the first trimester for avoidance of fetal malformation, and the adverse effects of hyperglycaemia throughout pregnancy. Insulin is regarded as the natural means of improving blood glucose control in pregnancy where lifestyle measures fail, although metformin is increasingly regarded as safe. Frequent self-monitoring of blood glucose is a normal part of insulin therapy, in particular where insulin requirements are changing as in pregnancy, and where stricter targets may lead to increased risk of serious hypoglycaemia [2]. Special considerations surrounding labour and delivery have been reviewed recently [11].

Experience with rapid-acting insulin analogues has been reassuring for insulin lispro although no formal trials are available [12]. Experience with long-acting insulin analogues is still very thin [2], and unless other clear advantage is apparent (previous major gain in blood glucose control over NPH insulin-based regimens) they are not generally used in pregnancy. Use of oral glucose-lowering drugs is still controversial, and mostly derives from experience of widespread use in some developing countries and in polycystic ovarian syndrome (see Canadian guideline [2]), but this mostly applies to glyburide and to metformin. Newer drugs are therefore assumed to be contra-indicated.

## Consideration

Despite the poor evidence-base, it is clear that the consequences of poor management of diabetes in pregnancy (high risk of maternal and neonatal complications, dead and deformed babies) are such that this is a prime area where investment of health-care resources is appropriate. Furthermore, considerable consensus exists over the need for continued monitoring of complications for acceleration of diabetes-induced damage, and the early use of insulin therapy to tight targets backed by self-monitoring. While the issue of methods and schedules for screening for new-onset diabetes in pregnancy is diverse and confused, the need for detection is not in dispute, and again there is clear consensus that the OGTT in some form has an important role, and that tight blood glucose management in those testing positive is indicated. Some other areas of care, such as the need for folic acid supplementation, and the high risk of future diabetes in those remitting from diabetes after delivery, also seem secure. A particularly difficult issue relates to the use of oral glucose-lowering drugs during pregnancy in places where insulin supply is tenuous, and Type 2 diabetes in pregnancy common. However, while it is nearly impossible to exclude a low incidence of adverse effects (<1 in 100), the potential gain – if this (glyburide/metformin) is the only means of improving glucose control – would seem to be higher.

## Implementation

Liaison with obstetric colleagues is a first step in implementation of these recommendations, such that joint protocols can be devised for screening for diabetes, and for pregnancy and post-pregnancy management. Health-care professionals need to be trained on pregnancy-specific lifestyle adaptation, insulin use, and complications screening. Availability of such staff needs to be assured. Where resources are scarce, the availability of insulin and self-monitoring equipment may need to be prioritized to this area, and supplies assured. Laboratory resources for clinical monitoring of glucose control and assessment of renal damage should be provided. Pre-pregnancy services may need to be organized separately.

## Evaluation

Monitoring of outcome of diabetic pregnancy (healthy and unhealthy neonates) may seem logical, but because of small number problems is not a powerful tool of quality assurance. Investigation of each neonatal death may be more useful. Delivery weight of the infant and achieved maternal $HbA_{1c}$ in each trimester are useful surrogate outcomes. Structural review should be of the existence of joint management protocols addressing the above recommendations, and appropriate availability of staff.

## References

1. WHO. Definition, Diagnosis and Classification of Diabetes Mellitus and its Complications. Report of a WHO Consultation. Part 1: Diagnosis and Classification of Diabetes Mellitus. Geneva: WHO Department of Noncommunicable Disease Surveillance, 1999: 1-59. http://www.who.int
2. Canadian Diabetes Association Clinical Practice Guidelines Expert Committee. Canadian Diabetes Association 2003 Clinical Practice Guidelines for the Prevention and Management of Diabetes in Canada. Canadian Journal of Diabetes 2003; 27(Suppl 2): S94-S105. http://www.diabetes.ca
3. European Diabetes Policy Group 1999. A desktop guide to Type 2 diabetes mellitus. Diabet Med 1999; 16: 716-30. http://www.staff.ncl.ac.uk/philip.home/guidelines
4. Scottish Intercollegiate Guidelines Network. SIGN 55. Management of Diabetes, 2001. http://www.sign.ac.uk
5. American Diabetes Association Position Statement. Standards of Medical Care in Diabetes. Diabetes Care 2005; 28 (Suppl 1): S4-S36.
6. American Diabetes Association. Gestational Diabetes Mellitus. Diabetes Care 2004; 27 (Suppl 1): S88-S90.
7. Crowther CA, Hiller JE, Moss JR, McPhee AJ, Jeffries WS, Robinson JS, for the Australian Carbohydrate Intolerance Study in Pregnant Women (ACHOIS) Trial Group. Effect of treatment of gestational diabetes mellitus on pregnancy outcomes. N Engl J Med 2005; 352: 2477-86.
8. Reichelt, AJ, Spichler ER, Branchtein L, Nucci LB, Franco LJ, Schmidt MI, for the Brazilian Study of Gestational Diabetes (EBDG) Working Group. Fasting plasma glucose is a useful test for the detection of gestational diabetes. Diabetes Care 1998; 21: 1246-49.
9. Perucchini D, Fischer U, Spinas GA, Huch R, Huch A, Lehman R. Using fasting plasma glucose concentrations to screen for gestational diabetes mellitus: prospective population based study. BMJ 1999; 319: 812-15.
10. Schmidt MI, Matos MC, Reichelt AJ, Costa Forti A, de Lima L, Duncan BB, for the Brazilian Gestational Diabetes Study Group. Prevalence of gestational diabetes – do the new WHO criteria make a difference? Diabet Med 2000; 17: 376-80.
11. Jovanovic L. Glucose and insulin requirements during labor and delivery: the case for normoglycemia in pregnancies complicated by diabetes. Endocr Pract 2004; 10(Suppl 2): 40-45.
12. Wyatt JW, Frias JL, Hoyme HE, Jovanovic L, Kaaja R, Brown F, et al. Congenital anomaly rate in offspring of mothers with diabetes treated with insulin lispro during pregnancy. Diabet Med 2005; 22: 803-07.

# Children

18

## Recommendations

### ■ Standard care

CH1 Diagnose symptomatic children using plasma glucose and WHO 1999 criteria [1].

CH2 Attempt to assign type of diabetes, using history and physical examination, including weight, BMI, urine ketones, pH, electrolytes.

When the diabetes appears to be Type 2 diabetes, remain alert to the possibility and associated risks of Type 1 diabetes or MODY.

Where differentiation is uncertain, islet-cell related antibodies and C-peptide estimation may add further information.

CH3 Provide initial care appropriate to age and developmental stage, including lifestyle counselling, diabetes education with the family, blood glucose monitoring, management with insulin or oral agents (metformin) according to clinical features, and psychological assessment.

CH4 Provide continuing care and support including:

- lifestyle measures in the context of the family
- self-monitoring of blood glucose, with attention to continuity from the management team, and to ensure care for diabetes at school
- $HbA_{1c}$ every 2-6 months (see *Clinical monitoring*).

CH5 Arrange annual surveillance including weight and height, BMI, blood pressure, urine protein and albumin, eye review.

### ■ Comprehensive care

$CH_C1$ Screening might also be extended to asymptomatic children who are at high risk in the particular population (criteria might include BMI, family history, age, race/ethnicity, insulin resistance as evidenced by acanthosis nigricans).

$CH_C2$ Attempts to assign the type of diabetes after diagnosis could also include more routine testing for islet-cell related antibodies and C-peptide, and HNF and glucokinase genotyping.

$CH_C3$ Initial care will be as for *Standard care*, while continuing care may also include routine psychosocial support; ongoing surveillance may include lipid profile.

## ■ Minimal care

$CH_M1$ Diagnose symptomatic children by urine glucose or capillary plasma glucose.

$CH_M2$ Attempt to assign type of diabetes by history and physical examination assessing weight, BMI, blood pressure, and urine ketones.

$CH_M3$ Initial care should include lifestyle information, diabetes education with the family, monitoring of blood glucose, and management with insulin and/or metformin according to clinical features.

$CH_M4$ Provide continuing care including:
- lifestyle measures in the context of the family
- advice to the school on dealing with emergencies and avoiding discrimination.

$CH_M5$ Surveillance will include weight, height, BMI, blood pressure, urine protein, and eye review.

## Rationale

Type 2 diabetes in children is increasing in many populations around the world. Affected children may have a positive family history of Type 2 diabetes, and in most cases the BMI is above the 85th percentile for gender and age, defined as overweight. However, this is not universal, notably in some Asian and Oriental populations. Overweight in childhood is associated with poverty in relatively developed areas but with affluence in developing areas of the world. Type 2 diabetes in children is a severe disease with very poor outcomes over 10-20 years. It is associated with significant islet B-cell failure as well as insulin resistance, and is at least as demanding to manage as Type 1 diabetes in children. Children with Type 2 diabetes are more at risk of hypertension, dyslipidaemia and polycystic ovarian syndrome than those with Type 1 diabetes.

## Evidence-base

It is only relatively recently that the emergence of Type 2 diabetes in children has been recognized. In Europid populations Type 1 diabetes remains the predominant form in children, but in Japanese populations 80 % of childhood diabetes is Type 2 diabetes, and the condition is increasing in incidence and prevalence in many parts of the world. It is usually diagnosed after the age of 10 yr, in mid- to late-puberty, with the reduced insulin sensitivity of puberty apparently playing a role [2]. The evidence-base remains limited, and only the Canadian guideline deals specifically with the condition [3]. There is a NICE guideline on Type 1 diabetes in children, and this refers briefly to the need to distinguish children with Type 2 diabetes [4]. Many of the global issues, and the paucity of evidence, were considered at an IDF meeting in 2003 [5], while the topic has been addressed in a number of US publications [6-10].

Use of adult diagnostic criteria [1] reflects lack of other evidence and the problems of staging and normative values in the 10- to 13-year age group. The Canadian guideline states that insulin is required when there is severe metabolic decompensation at diagnosis (ketoacidosis, $HbA_{1c}$ ≥9.0 %, symptoms of severe hyperglycaemia); otherwise the recommended initial treatment is intensive lifestyle intervention, adding metformin as first-line therapy if glycaemic targets are not achieved [3]. An algorithm devised by Silverstein and Rosenbloom in a review of North American practice [6] suggests that in those started on insulin (plus lifestyle) achievement of a DCCT-aligned $HbA_{1c}$ <7.0 % allows tapering of insulin dose with addition of metformin, and attempts to 'wean off' insulin. However, the evidence-base for treatment is very limited, with data on insulin use mainly from Type 1 diabetes. The Canadian guideline cites evidence for efficacy and safety of metformin (over 16 weeks) in adolescents with Type 2 diabetes, and draws attention to the contra-indications in the case of kidney or liver disease [3].

The gastro-intestinal side-effects of metformin are poorly tolerated by children and adolescents, yet other oral glucose-lowering options have barely been explored.

Recommendations on surveillance for complications reflect evidence on microvascular complications in Pima Indian and Japanese populations, cited in the Canadian guideline [3]. The risks of pregnancy in this age-group need to be borne in mind in relation to drug therapy.

## Consideration

Health-care professionals dealing with children need to be alert to the possibility of Type 2 diabetes, and aware of the seriousness of the condition. Most of these children are overweight at diagnosis, and most are in families with others who are overweight and at risk of Type 2 diabetes, so advice on lifestyle modification can usefully involve the whole family.

## Implementation

A continuing integrated package of care should be offered by a multidisciplinary paediatric diabetes team, trained in the difficult area of distinguishing Type 2 diabetes in children, outlining the pathways of care, and dealing with the possibility of multiple medication. Structured records and recall systems are essential, as is the need to address the transition to adult diabetes care services.

## Evaluation

Systematic evaluation of an emerging epidemic will include, at all levels, numbers of patients, medications given, and complications at diagnosis. Standard care should also include documentation of BMI, glycaemic control, and complications on follow-up, while comprehensive care should additionally evaluate efficacy of treatment, cost, and criteria used for diagnosis.

## References


1. WHO. Definition, Diagnosis and Classification of Diabetes Mellitus and its Complications. Report of a WHO Consultation. Part 1: Diagnosis and Classification of Diabetes Mellitus. Geneva: WHO Department of Noncommunicable Disease Surveillance, 1999: 1-59. http://www.who.int
2. Caprio S. Insulin: the other anabolic hormone of puberty. Acta Paediatr Suppl 1999; 88: 84-87.
3. Canadian Diabetes Association Clinical Practice Guidelines Expert Committee. Canadian Diabetes Association 2003 Clinical Practice Guidelines for the Prevention and Management of Diabetes in Canada. Canadian Journal of Diabetes 2003; 27(Suppl 2): S91-S93. http://www.diabetes.ca
4. National Institute for Clinical Excellence. Type 1 diabetes: diagnosis and management of type 1 diabetes in children, young people and adults. London: National Institute for Clinical Excellence, 2004. http://www.nice.org.uk/page.aspx?o=213575
5. The International Diabetes Federation Consensus Workshop. Type 2 diabetes in the young: the evolving epidemic. Diabetes Care 2004; 27: 1798-1811.
6. Silverstein JH, Rosenbloom AL. Treatment of type 2 diabetes mellitus in children and adolescents. J Pediatr Endocrinol Metab 2000; 13 (Suppl 6): 1403-09.
7. American Diabetes Association consensus statement. Type 2 diabetes in children and adolescents. Diabetes Care 2000; 23: 381-89.
8. American Diabetes Association consensus statement. Management of dyslipidaemia in children and adolescents with diabetes. Diabetes Care 2003; 26: 2194-97.
9. Diabetes in Children, Adolescents Work Group of the National Diabetes Education Program: An Update on Type 2 Diabetes in Youth from the National Diabetes Education Program. Pediatrics 2004; 114: 259-63.
10. Rosenbloom A, Silverstein J. Type 2 Diabetes in Children and Adolescents. Alexandria, VA, American Diabetes Association, 2003.

# In-patient care

19

## Recommendations

### ■ Standard care

***In-patient care organization***

HO1 Designate a diabetes-trained health-care professional to:
- manage and co-ordinate systems of care related to diabetes management of in-patients
- co-ordinate training of hospital staff in awareness of the needs of people with diabetes
- implement strategies to prevent disempowerment of those who could self-manage their diabetes
- plan for discharge and follow-up.

HO2 Provide access for people with diabetes and hospital staff to a multidisciplinary diabetes team.

HO3 Ensure laboratory/service support for:
- assays including plasma glucose, $HbA_{1c}$, basic haematology and biochemistry, lipid profile and hormone assays
- microbiological investigation
- radiology and other imaging.

***General ward care***

HO4 Encourage self-management of diabetes (food choice, self-monitoring, insulin dose adjustment where appropriate) integrated into usual ward care.

***Management during in-patient procedures***

HO5 Evaluate blood glucose control, and metabolic and vascular complications (in particular renal and cardiac status) prior to planned procedures; provide advice on the management of diabetes on the day or days prior to the procedure.

HO6 Ensure the provision and use of an agreed protocol for in-patient procedures and surgical operations.

HO7 Aim to maintain near-normoglycaemia without hypoglycaemia by regular quality-assured blood glucose testing and intravenous insulin delivery where needed, generally using a glucose/insulin/potassium infusion.

HO8 Ensure awareness of special risks to people with diabetes during hospital procedures, including risks from:

- neuropathy (heel ulceration, cardiac arrest)
- intra-ocular bleeding from new vessels (vascular and other surgery requiring anticoagulation)
- drug therapy (risks of acute renal failure causing lactic acidosis in people on metformin, for example with radiological contrast media).

***Critical care situations***

HO9 Provide access to intensive care units (ICU) for life-threatening illness, ensuring that strict blood glucose control, usually with intravenous insulin therapy, is a routine part of system support for anyone with hyperglycaemia.

HO10 Provide protocol-driven care to ensure detection and immediate control of hyperglycaemia for anyone with a presumed acute coronary event or stroke, normally using intravenous insulin therapy with transfer to subcutaneous insulin therapy once stable and eating.

## ■ Comprehensive care

$HO_C1$ General principles are as for *Standard care*, but would include repeated review by a diabetes specialist where general health state is changing or glucose control is problematic.

$HO_C2$ Use telematic review of blood glucose control to a specialist's office for people in critical situations.

$HO_C3$ Maintain staff trained in aspects of diabetes management on any ward or procedure area with a significant throughput of people with diabetes.

## ■ Minimal care

$HO_M1$ General principles are as for *Standard care*, but hospitals should designate an individual in charge of matters relating to in-patient diabetes, to co-ordinate training in awareness of the needs of, and provision of in-patient care to, people with diabetes, and the provision and use of guidelines and protocols.

$HO_M2$ Laboratory assays should include plasma glucose and basic biochemistry; basic radiology should be available.

$HO_M3$ Management of plasma glucose levels during in-patient procedures will generally be as for *Standard care*. Where this is impossible or carries special risk, frequent intramuscular insulin with frequent monitoring may be useful in emergency situations, or frequently monitored subcutaneous insulin therapy (e.g. with NPH insulin) for minor procedures or more stable health states.

## Rationale

Hyperglycaemia is found, and requires management, in hospital settings not only in people with known diabetes but also in people with previously unrecognized diabetes and in people with hospital-related hyperglycaemia which reverts to normal after discharge. Prevalence of diabetes in hospitalized adult patients is 12-25 % or more [1]. Hospital care for people with diabetes may be required for metabolic emergencies, in-patient stabilization of diabetes, diabetes-related complications, intercurrent illnesses, surgical procedures, and labour and delivery (see *Pregnancy*).

## Evidence-base

Recent growth in the literature on hospital hyperglycaemia is reflected in the inclusion of sections on in-patient management in diabetes guidelines. The 2005 ADA standards have added a section on diabetes care in the hospital [1], drawing on a technical review [2] and the position statement of the American College of Endocrinology (ACE) [3]. The Canadian guidelines include separate sections on peri-operative and peri-acute coronary syndrome glycaemic control [4]. NICE reviewed evidence from people with Type 2 diabetes when developing recommendations for in-patient care in Type 1 diabetes [5].

The recent ACE position statement was based on a review of the literature on in-hospital hyperglycaemia [3]. They found multiple studies confirming that hospitalized patients with hyperglycaemia suffer significant excess mortality and morbidity, prolonged length of stay, unfavourable post-discharge outcomes, and significant excess health-care costs. They found RCTs as well as prospective observational and retrospective studies demonstrating improved outcomes (mortality, infection, intubation time, length of hospital stay) resulting from more aggressive treatment of hyperglycaemia. They strongly support the need for early detection of hyperglycaemia in the hospital and an aggressive management approach to improve outcomes.

ACE propose upper limits for blood glucose targets (ICU 6.1 mmol/l (110 mg/dl); non-ICU 6.1 mmol/l pre-prandial, 10.0 mmol/l (180 mg/dl) maximum), with the proviso that those for non-intensive care patients are less well supported by the evidence. They list indications for intravenous insulin infusion therapy (critical illness, prolonged nil-by-mouth status in insulin-deficient patients, peri-operative period, post transplantation, total parenteral nutrition therapy, elevated glucose exacerbated by high-dose glucocorticoid therapy, stroke, dose-finding prior to subcutaneous (SC) insulin injections, other illnesses requiring prompt glucose control). For SC insulin they discourage the use of sliding scales. They found some evidence for a diabetes team approach (reduced length of stay, fewer re-admissions).

The Canadian guidelines also make recommendations on blood glucose levels, emphasizing tight control (4.5-6.0 mmol/l, 80-110 mg/dl) for post-operative ICU patients if random plasma glucose >6.1 mmol/l (>110 mg/dl) [4]. They found strong evidence for recommending that all patients with acute MI and blood glucose >12.0 mmol/l (>215 mg/dl) should receive insulin-glucose infusion therapy to maintain blood glucose between 7.0 and 10.0 mmol/l (125-180 mg/dl) for at least 24 h, followed by multi-dose SC insulin for at least 3 months.

Neither ACE nor the Canadian guideline addresses the issue of oral glucose-lowering drugs in the hospital setting, but the ADA [1] draws attention to limitations for in-patient use (especially with regard to flexibility) of the major classes. For metformin, the fact that many specific contra-indications (related to risks of renal impairment) to its use are found in the hospital setting was seen as limiting its use. For thiazolidinediones haemodynamic changes were felt to be an issue, and for sulfonylureas risk of hypoglycaemia.

One cost study, cited by ACE, found cost per QALY for intravenous insulin therapy in patients with acute myocardial infarction to be comparable to that for other well-accepted medical interventions.

NICE additionally notes the utility and importance of a holistic approach, using the skills and knowledge of a person with diabetes developed over years or decades [5].

## Consideration

It was considered important that hospitals should designate a 'diabetes lead' individual, who would be in charge of matters relating to diabetes, and could co-ordinate training of staff in awareness of the needs of those with diabetes, and develop strategies to prevent disempowerment of those who could self-manage their diabetes. Major considerations were that diabetes should not complicate the management of whatever condition resulted in admission to hospital, and that a person's diabetes should not emerge from hospital worse than when they were admitted. While the evidence over use of protocol-driven intravenous insulin regimens is not conclusive, the widespread and general adoption of these regimens globally appears telling (for more detail of methods see references 6, 7).

## Implementation

Systems of care and protocols need to be put in place and staff trained to ensure their effectiveness. Standardized protocols, developed by multidisciplinary teams, should specify insulin dose, include guidelines for identifying patients at risk for hypoglycaemia, and actions to be taken to prevent and treat hypoglycaemia. Bedside glucose monitoring requires defined administrative responsibility, a procedure manual, training, policies regarding frequency (hourly to twice-daily) and procedures for alert values, quality control, and regular maintenance of equipment.

## Evaluation

Evaluation should consider evidence of the availability of trained staff (and training courses) and of protocols as above. Audit can be made of ward blood glucose control, and blood glucose control during surgery, after myocardial infarction and in intensive care. Admissions to coronary care can be reviewed to ensure measurement of blood glucose is occurring, and appropriate actions are then taken while in the unit and during follow-up.

## References

1. American Diabetes Association Position Statement. Standards of Medical Care in Diabetes. Diabetes Care 2005; 28 (Suppl 1): S4-S36.
2. Clement S, Braithwaite SS, Magee MF, Ahmann A, Smith EP, Schafer RG, et al. Management of diabetes and hyperglycemia in hospitals. Diabetes Care 2004; 27: 553-97.
3. Garber AJ, Moghissi ES, Bransome ED Jr, Clark NG, Clement S, Cobin RH, et al. American College of Endocrinology position statement on inpatient diabetes and metabolic control. Endocr Pract 2004; 10: 77-82.
4. Canadian Diabetes Association Clinical Practice Guidelines Expert Committee. Canadian Diabetes Association 2003 Clinical Practice Guidelines for the Prevention and Management of Diabetes in Canada. Canadian Journal of Diabetes 2003; 27(Suppl 2): S113-S116. http://www.diabetes.ca
5. The National Collaborating Centre for Chronic Conditions. Type 1 Diabetes in Adults. National clinical guideline for diagnosis and management in primary and secondary care. http://www.rcplondon.ac.uk/pubs/books/DIA/index.asp
6. Gill GV, Alberti KGMM. The care of the diabetic patient during surgery. In: DeFronzo RA, Ferrannini E, Keen H, Zimmet P (eds) International Textbook of Diabetes Mellitus, 3rd edn. Chichester: Wiley, 2004: pp 1741-51.
7. European Diabetes Policy Group 1999. A desktop guide to Type 2 diabetes: management of diabetes during surgery. Diabet Med 1999; 16: 729-30. http://www.staff.ncl.ac.uk/philip.home/t2dgch6a.htm

# Acronyms and abbreviations

| | |
|---|---|
| A2RB | angiotensin-II receptor blocker |
| ACE | American College of Endocrinology |
| ACE-inhibitor | angiotensin converting enzyme inhibitor |
| ACR | albumin:creatinine ratio |
| ADA | American Diabetes Association |
| AER | albumin excretion rate |
| BMI | body mass index |
| BP | blood pressure |
| CCB | calcium-channel blocker |
| CCT | controlled clinical trial |
| CDA | Canadian Diabetes Association |
| CV | cardiovascular |
| CVD | cardiovascular disease |
| DCCT | Diabetes Control and Complications Trial |
| DSME | diabetes self-management education |
| eGFR | estimated glomerular filtration rate |
| FPG | fasting plasma glucose |
| GDM | gestational diabetes |
| Hb | haemoglobin |
| HDL | high density lipoprotein |
| HNF | hepatocyte nuclear factor |
| HPLC | high-performance liquid chromatography |
| ICSI | Institute for Clinical Systems Improvement |
| ICU | intensive care unit |
| LDL | low density lipoprotein |
| MI | myocardial infarction |
| MNT | medical nutrition therapy |
| MODY | maturity-onset diabetes of the young |
| MRI | magnetic resonance imaging |
| NICE | National Institute for Clinical Excellence (England and Wales) |
| NPH | neutral protamine Hagedorn |
| OGTT | oral glucose tolerance test |
| PCR | protein:creatinine ratio |
| PDE5 | phosphodiesterase type-5 |
| QALY | quality-adjusted life year |
| RCT | randomized controlled trial |
| SC | subcutaneous |
| SIGN | Scottish Intercollegiate Guidelines Network |
| SMBG | self-monitoring of blood glucose |
| UKPDS | United Kingdom Prospective Diabetes Study |
| WHO | World Health Organization |

## Disclaimer

The International Diabetes Federation (IDF) does not provide individualized medical diagnosis, treatment or advice, nor does it recommend specific therapies or prescribe medication for anyone using or consulting the *Global Guideline for Type 2 Diabetes*. IDF is not engaged in the practice of medicine and nothing contained in the *Global Guideline for Type 2 Diabetes* is intended to constitute professional advice for medical diagnosis or treatment for specific persons. The information contained in the *Global Guideline for Type 2 Diabetes* is intended and may be used for general educational and informational purposes only.

Medical information changes rapidly; therefore, some of the information contained in the *Global Guideline for Type 2 Diabetes* may be out of date and/or may contain inaccuracies. IDF assumes no responsibility for how readers use the information contained in the *Global Guideline for Type 2 Diabetes*. Readers, in search of personal medical advice and direction, should seek advice from and consult with appropriately qualified medical and health-care professionals on specific situations and conditions of concern.

Reliance on information contained in the *Global Guideline for Type 2 Diabetes* is solely at the reader's own risk. Readers should exercise independent judgement and scepticism before applying any information contained in the *Global Guideline for Type 2 Diabetes* to their own health needs, or relying on any information in any other way. No one person's medical needs are the same as another person's, and therefore, information provided may be incorrect or misleading.

Reasonable endeavours have been made to ensure the accuracy of the information presented. However, IDF assumes no legal liability or responsibility for the accuracy, currency or completeness of the information provided herein. Any views, opinions, and/or recommendations contained in this publication are not those of IDF or endorsed by IDF, unless otherwise specifically indicated by IDF. The International Diabetes Federation assumes no responsibility or liability for personal or other injury, loss, or damage that may result from the information contained within this publication.

Printed in August 2005
by Paperland printers, Belgium

Production manager: Luc Vandensteene
Cover design and layout: Ex Nihilo

© International Diabetes Federation 2005
Avenue Emile de Mot, 19
B-1000 Brussels

International Diabetes Federation

**International Diabetes Federation (IDF)**
Avenue Emile de Mot 19 • B-1000 Brussels • Belgium • Phone: +32-2-5385511 • Fax: +32-2-5385114
www.idf.org • communications@idf.org
